# A planarian nidovirus expands the limits of RNA genome size

**DOI:** 10.1371/journal.ppat.1007314

**Published:** 2018-11-01

**Authors:** Amir Saberi, Anastasia A. Gulyaeva, John L. Brubacher, Phillip A. Newmark, Alexander E. Gorbalenya

**Affiliations:** 1 Howard Hughes Medical Institute, Department of Cell and Developmental Biology, University of Illinois at Urbana-Champaign, Urbana, IL, United States of America; 2 Department of Medical Microbiology, Leiden University Medical Center, Leiden, The Netherlands; 3 Department of Biology, Canadian Mennonite University, Winnipeg, Canada; 4 Faculty of Bioengineering and Bioinformatics, Lomonosov Moscow State University, Moscow, Russia; University of Iowa, UNITED STATES

## Abstract

RNA viruses are the only known RNA-protein (RNP) entities capable of autonomous replication (albeit within a permissive host environment). A 33.5 kilobase (kb) nidovirus has been considered close to the upper size limit for such entities; conversely, the minimal cellular DNA genome is in the 100–300 kb range. This large difference presents a daunting gap for the transition from primordial RNP to contemporary DNA-RNP-based life. Whether or not RNA viruses represent transitional steps towards DNA-based life, studies of larger RNA viruses advance our understanding of the size constraints on RNP entities and the role of genome size in virus adaptation. For example, emergence of the largest previously known RNA genomes (20–34 kb in positive-stranded nidoviruses, including coronaviruses) is associated with the acquisition of a proofreading exoribonuclease (ExoN) encoded in the open reading frame 1b (ORF1b) in a monophyletic subset of nidoviruses. However, apparent constraints on the size of ORF1b, which encodes this and other key replicative enzymes, have been hypothesized to limit further expansion of these viral RNA genomes. Here, we characterize a novel nidovirus (planarian secretory cell nidovirus; PSCNV) whose disproportionately large ORF1b-like region including unannotated domains, and overall 41.1-kb genome, substantially extend the presumed limits on RNA genome size. This genome encodes a predicted 13,556-aa polyprotein in an unconventional single ORF, yet retains canonical nidoviral genome organization and expression, as well as key replicative domains. These domains may include functionally relevant substitutions rarely or never before observed in highly conserved sites of RdRp, NiRAN, ExoN and 3CLpro. Our evolutionary analysis suggests that PSCNV diverged early from multi-ORF nidoviruses, and acquired additional genes, including those typical of large DNA viruses or hosts, e.g. Ankyrin and Fibronectin type II, which might modulate virus-host interactions. PSCNV's greatly expanded genome, proteomic complexity, and unique features–impressive in themselves–attest to the likelihood of still-larger RNA genomes awaiting discovery.

## Author summary

RNA viruses are the only known RNA-protein (RNP) entities capable of autonomous replication. The upper genome size for such entities was assumed to be <35 kb; conversely, the minimal cellular DNA genome is in the 100–300 kilobase (kb) range. This large difference presents a daunting gap for the proposed evolution of contemporary DNA-RNP-based life from primordial RNP entities. Here, we describe a nidovirus from planarians, named planarian secretory cell nidovirus (PSCNV), whose 41.1 kb genome is 23% larger than any riboviral genome yet discovered. This increase is nearly equivalent in size to the entire poliovirus genome, and it equips PSCNV with an unprecedented extra coding capacity to adapt. PSCNV has broken apparent constraints on the size of the genomic subregion that encodes core replication machinery in other nidoviruses, including coronaviruses, and has acquired genes not previously observed in RNA viruses. This virus challenges and advances our understanding of the limits to RNA genome size.

## Introduction

Radiation of primitive life as it took hold on earth was likely accompanied by genome expansion, which was associated with increased complexity and a proposed progression from RNA-based through RNA-protein to DNA-based life [1]. The feasibility of an autonomous ancient RNA genome, and the mechanisms underlying such fateful transitions, are challenging to reconstruct. It is especially unclear whether RNA entities ever evolved genomes close to the 100–300 kilobase (kb) range [2, 3] of the “minimal” reconstructed cellular DNA genome [4]. This range overlaps with the upper size limit of nuclear pre-mRNAs [5], which is likely the upper size limit for functional RNAs due to the relative chemical lability of RNA compared to DNA. However, pre-mRNAs are incapable of self-replication, the defining property of primordial genomic RNAs.

RNA viruses may uniquely illuminate the evolutionary constraints on RNA genome size [6–9], whether or not they descended directly from primitive RNA-based entities [10–13]. The same constraints may also inform research on the biology and pathogenesis of RNA virus infections, because they shape the diversity of viral proteomes and RNA elements. The causes and consequences of changes in genome size can be understood in the context of a relationship that locks replication fidelity, genome size, and complexity within a unidirectional triangle [14]. RNA viruses appear to be trapped in the low state of this relationship (Eigen trap) [15], which is characterized by low fidelity (high mutation rate), small genome size (10 kb average), and low complexity (few protein/RNA elements). Specifically, low-fidelity replication without proofreading constrains genome expansion [16], since accumulation of mutations [17] would lead to the meltdown of larger genomes during replication (error catastrophe hypothesis) [18, 19].

**Fig 1:**
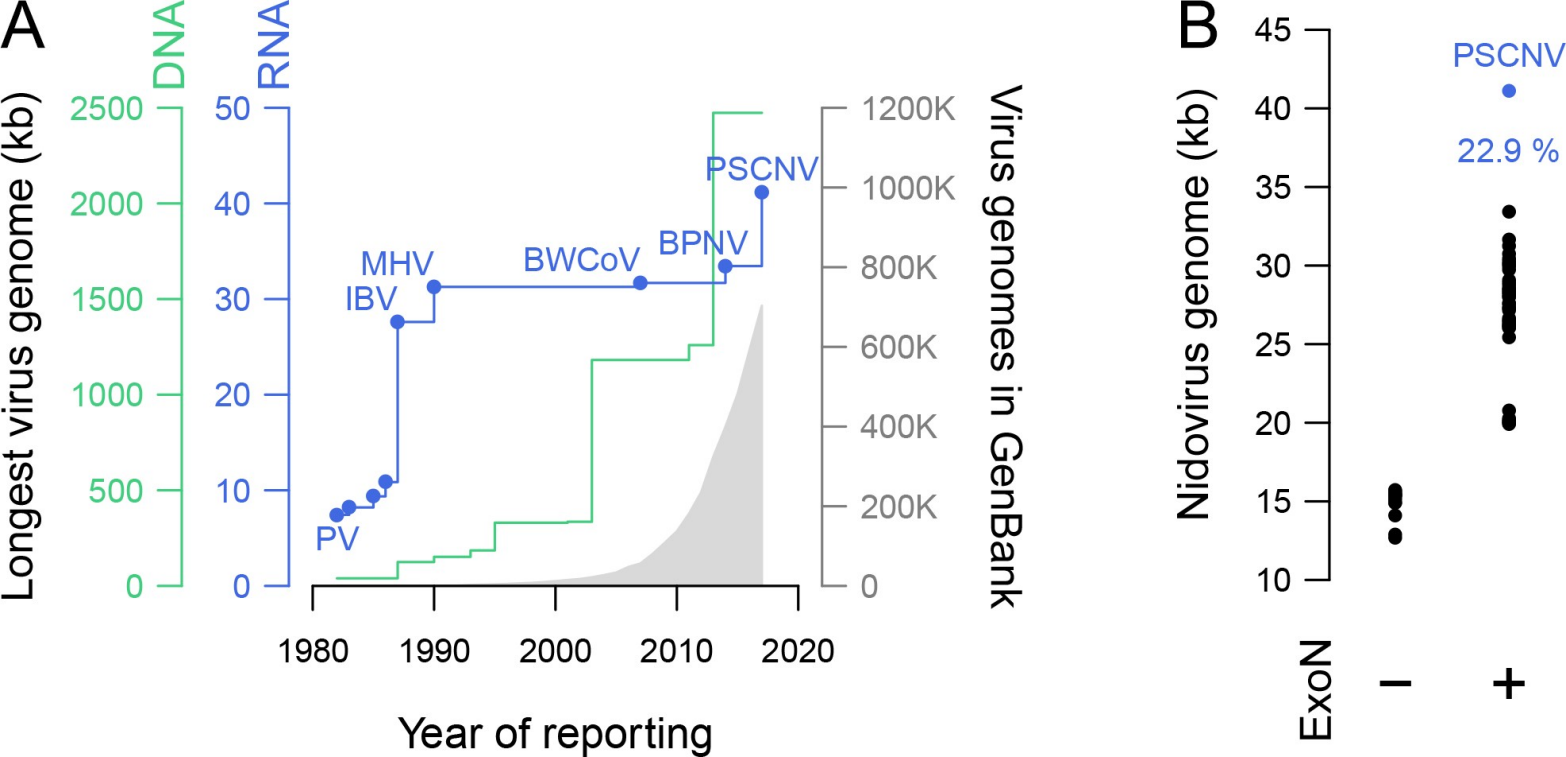
Genome sizes of nidoviruses. (***A***) Timeline of discovery of largest RNA and DNA virus genomes versus accumulation of virus genome sequences in GenBank (1982–2017). PV, poliovirus; and nidoviruses: IBV, avian infectious bronchitis virus, MHV, mouse hepatitis virus, BWCoV, beluga whale coronavirus SW1, BPNV, ball python nidovirus and PSCNV, planarian secretory cell nidovirus. (***B***) Comparison of genome sizes between nidoviruses that do not encode an ExoN domain, and those that do. Percentage indicates the difference between sizes of PSCNV and the next-largest entity.

This constraining relationship is supported by evidence from nidoviruses (order *Nidovirales*): enveloped viruses with positive-stranded RNA genomes in the range of 12.7 to 33.5 kb–the largest known RNA genomes [20–23] (Fig 1A and 1B, S1 Table). The *Nidovirales* is composed of two vertebrate families, *Arteriviridae* and *Coronaviridae* (subfamilies *Coronavirinae* and *Torovirinae*), and two invertebrate families, *Mesoniviridae* and *Roniviridae* [24, 25], and includes important pathogens of humans (Severe acute respiratory syndrome coronavirus, SARS-CoV; Middle East respiratory syndrome coronavirus, MERS-CoV) and livestock (different arteriviruses, coronaviruses and roniviruses) [26–30]. All known nidoviruses with genomes larger than 20 kb also encode a proofreading exoribonuclease (ExoN) [14, 31–34] (Fig 1B), which, once acquired by an ancestral nidovirus, may have relieved the constraints on all three elements of the triangular relationship *simultaneously*, providing a solution to the Eigen trap [14].

In the last 20 years of virus discovery, however, despite the application of unbiased metagenomics to RNA virus discovery [35, 36], the largest-known RNA viral genome has only increased ~10% in size–a mere fraction of the nearly ten-fold increase observed for DNA viruses [37–39] (Fig 1A). Thus, other constraints have apparently limited genome size, even in RNA viruses equipped with proofreading capability. Further characterization of nidovirus molecular biology, variation, and evolution may provide insight into these other factors.

**Fig 2:**
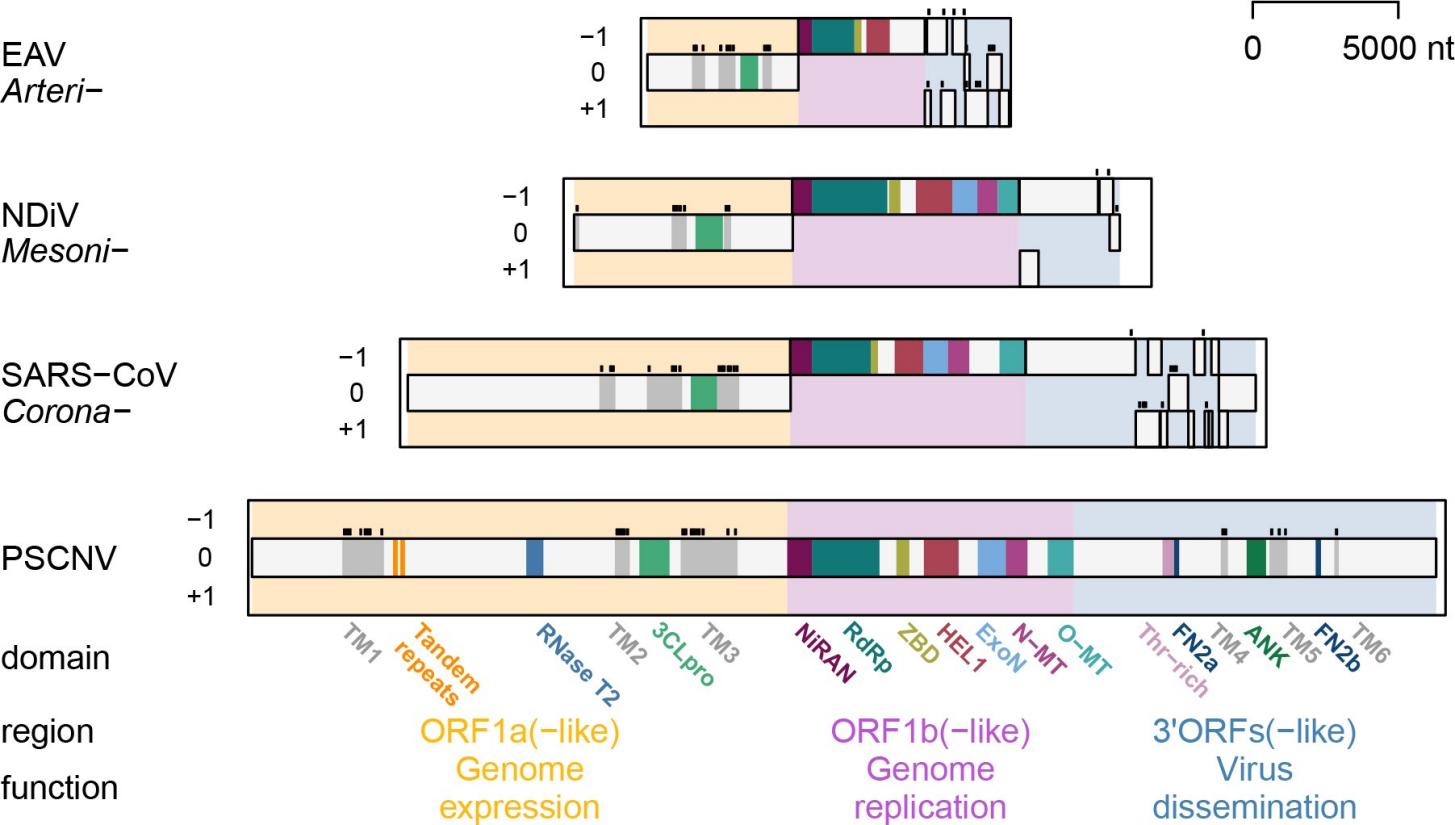
Genomes and proteomes of nidoviruses. ORFs and encoded protein domains in genomes of viruses representing three nidovirus families and PSCNV. The protein-encoding part of the genomes is split in three adjacent regions, which are colored and labelled accordingly. EAV, equine arteritis virus; NDiV, Nam Dinh virus; SARS-CoV (see S1 Table for details on these viruses). ORF1a frame is set as zero. Protein domains conserved between these nidoviruses and PSCNV, and those specific to PSCNV are shown. TM, transmembrane domain (TM helices are shown by black bars above TM domains); Tandem repeats, two adjacent homologous regions of unknown function; RNase T2, ribonuclease T2 homolog; 3CLpro, 3C-like protease; NiRAN, nidovirus RdRp-associated nucleotidyltransferase; RdRp, RNA-dependent RNA polymerase; HEL1, superfamily 1 helicase with upstream Zn-binding domain (ZBD); ExoN, DEDDh subfamily exoribonuclease; N-MT and O-MT, SAM-dependent N7- and 2’-O-methyltransferases, respectively; Thr-rich, region enriched with Thr residue; FN2a/b, fibronectin type 2 domains; ANK, ankyrin domain.

Nidovirus genomes are typically organized into many open reading frames (ORFs), which occupy >90% of genome and can be divided into three regions: overlapping ORF1a and ORF1b, and multiple ORFs at the 3’-end (3’ORFs) [14] (Fig 2). The products of these regions predominantly control genome expression/replication, and virus assembly/dissemination, respectively.

ORF1a and ORF1b are expressed by translation of the genomic RNA that involves a -1 programmed ribosomal frameshifting (PRF) at the ORF1a/ORF1b overlap [40, 41]. The two polyproteins produced without or with frameshifting, pp1a (ORF1a-encoded) and pp1ab (ORF1a/ORF1b-encoded), vary in size from 1,727 to 8,108 aa. They are processed to a dozen or more proteins by the virus’ main protease (3CLpro, encoded in ORF1a; Fig 2) with possible involvement of other protease(s) [42]. These and other proteins form a membrane-bound replication-transcription complex (RTC) [43, 44] that invariably includes two key ORF1b-encoded subunits: the Nidovirus RdRp-Associated Nucleotidyltransferase (NiRAN) fused to an RNA-dependent RNA polymerase (RdRp) [45, 46], and a zinc-binding domain (ZBD) fused to a superfamily 1 helicase (HEL1), respectively [47–50]. The RTC catalyzes the synthesis of genomic and 3’-coterminal subgenomic RNAs, the latter via discontinuous transcription that is regulated by leader and body transcription-regulating sequences (lTRS and bTRS) [51–53]. Subgenomic RNAs are translated to express virion and, in ExoN-positive viruses, accessory proteins encoded in the 3’ORFs [23, 54–59]. Most nidovirus proteins are multifunctional, but some released from the N-terminus of pp1a/pp1ab and/or encoded in the 3’ORFs are specialized in the modulation of virus-host interactions [26, 60–65].

Intriguingly, despite the large variation in genome size among extant nidoviruses, the size of ORF1b varies extremely little within either the ExoN-negative (12.7–15.7 kb genome range) or ExoN-positive (19.9–33.5 kb genome range) nidoviruses [66]. There is no overlap between these two groups of viruses in the size range of ORF1b: the smallest ORF1b of an ExoN-positive nidovirus is almost double the length of the largest ExoN-negative ORF1b. In contrast, the ORF1a and 3’ORFs regions exhibit considerable size variation, and their sizes overlap between the ExoN-positive and ExoN-negative clades.

A current theoretical model of nidoviral genome dynamics, the three-wave model, proposes that a genome expansion cycle is initiated by a bottleneck increase of ORF1b (the first wave) in a common ancestor of ExoN-positive nidoviruses, which then permits parallel expansion of ORF1a and, often, 3’ORFs in subsequent overlapping waves in separate lineages [66]. Extant nidovirus genomes of different sizes have reached particular points on this trajectory of genome size, apparently due to the lineage-specific interplay of poorly understood genetic and host-specific factors. A single cycle of this process can account for genome expansion from the lower end of genome sizes (12.7 kb) to the upper end (31.7 kb); expansion of genomes far beyond that size range has been hypothesized to require a second cycle, beginning with a new wave of ORF1b expansion [66]. In the absence of newly discovered RNA viruses with significantly larger genomes since the time of that analysis, and due to the unknown nature of the ORF1b size constraint(s), however, the feasibility of a second cycle has remained uncertain, and the notion that ~34 kb is close to the actual limit of RNA virus genome size [35] has seemed plausible.

To examine whether this limit applies beyond the currently recognized ~3000 RNA virus species (isolated from only a few hundred host species), further sampling of virus diversity is required, particularly from host species in which viruses have thus far remained virtually unknown. To this end, we analyzed *de novo* transcriptomes from both major reproductive biotypes (strains) of the planarian *Schmidtea mediterranea* [67]: a hermaphroditic sexual strain, and an asexual strain whose members reproduce via transverse fission [68]. We report the discovery and characterization of the first known planarian RNA virus, dubbed the planarian secretory cell nidovirus. PSCNV has the largest RNA genome by a considerable margin–a feat made more remarkable by the fact that its genome is organized as a single ORF. Concomitantly, it has adapted the nidoviral regulatory toolkit in novel ways, and acquired many features that revise the known limits of viral genomic and proteomic variation–some of these features being unique among nidoviruses, others among RNA viruses, and still others among all known viruses. Our results imply that viruses with the nidoviral genetic plan have the potential to expand RNA genomes further along the trajectory envisioned by the multi-cycle, three-wave model.

## Results

### Identification and genomic assembly of a large RNA virus from planarians

To identify potential nidovirus-like sequences in the planarian transcriptome, we queried two in-house *de novo-*assembled *Schmidtea mediterranea* transcriptomes [67] for sequences that significantly resembled a reference coronavirus genome. Two nearly identical (99.97%) nested transcripts, txv3.2-contig_1447 (originating from the sexual strain) and txv3.1-contig_12746 (from the asexual strain), showed a statistically significant similarity to known nidoviruses as reciprocal BLAST top hits. We hypothesized that these transcripts are genomic fragments of a new nidovirus species. We further identified several overlapping EST clones with >99% nucleotide identity to the transcriptome contigs, and assembled these into a putative partial genome (S1 Fig). Finally, with additional transcriptome search iterations and Sanger sequencing of the transcript 5’-end, we assembled a 41,103-nt transcript (excluding the polyA tail). Based on several criteria (see below), we assigned this RNA sequence to the genome of a virus we dubbed Planarian Secretory Cell Nidovirus (PSCNV) (S1 Fig). This sequence was the reference genome used for further analyses (see Materials and Methods for more detail).

The complete PSCNV genome encodes a single 40,671-nt ORF that is flanked by a 128-nt 5’-UTR and a 304-nt 3’-UTR (Figs 1B and 2). In addition, we found the main ORF overlapping multiple small ORFs in other reading frames, whose lengths exceeded 150 nt: 8 ORFs in the same strand as the large ORF (plus-strand), lengths ranging from 156 to 267 nt, 5 of which mapped to the 3’-terminal quarter of the genome; and 24 ORFs in the reverse complement strand (minus-strand), distributed throughout the genome, with lengths ranging from 153 to 681 nt. To further verify the presence of the viral genome *in vivo*, we amplified large overlapping genomic subregions by RT-PCR (S2 Table, S1 Fig) [69]. These sequences could not be amplified from *S*. *mediterranea* genomic DNA, nor could they be found in the reference planarian genome [70]; thus, they appear to derive from an exogenous source.

### PSCNV variants in worldwide planarian laboratories imply recent virus transmission

A survey of 16 *S*. *mediterranea* RNA-seq datasets from nine laboratories worldwide uncovered PSCNV reads in five datasets from three American locations. Of the positive datasets, three originated from the sexual strain, and two from the asexual strain. Overall, viral sequences were much more abundant in transcriptomes obtained from sexual strains (S3 Table).

The PSCNV sequences detected in these studies vary little from one another. The three most complete sequences (tentatively reconstructed from PRJNA319973, PRJNA79031, and PRJNA421285) are characterized by >99.9% identity across a nearly 13 kb span of the genome, where at least 2 reads (and at least 10 reads for >95% of positions) from each dataset mapped to each position of the reference genome. Indeed, sequences from PRJNA319973 and PRJNA79031 –the two datasets from the Newmark laboratory–exhibit only a single mutation relative to the reference genome, and the sequence from PRJNA421285 –from the Sanchez Alvarado laboratory–differs at only 9 positions (S4 Table). This low variation is notable, as two of the datasets analyzed (PRJNA79031 and PRJNA421285) are derived from sexual *S*. *mediterranea*, and the other one (PRJNA319973) from an asexual *S*. *mediterranea* lab strain. The source populations of these two (freshwater) strains are separated from each other by about 500 km of the Mediterranean Sea: the asexual laboratory strain was established from a population in Barcelona [71], and the sexual strain originates from a Sardinian population. A recent study of the evolutionary history of *S*. *mediterranea* suggests that these populations diverged from each other at least 4 million years ago [72].

Given the long-separate history of these two planarian strains prior to becoming research subjects and the relatively high mutation rate in characterized nidoviruses, the detection of nearly identical viral transcripts in both strains is strong evidence that the virus is transmissible. The absence of viral sequences from asexual strains in most labs, and their presence in all labs that have reported RNA-seq data from the sexual strain, strongly suggest that the virus first infected (or was endemic to) the sexual strain, and has subsequently spread to asexual laboratory stocks.

### PSCNV infects the secretory cells of planarians

**Fig 3:**
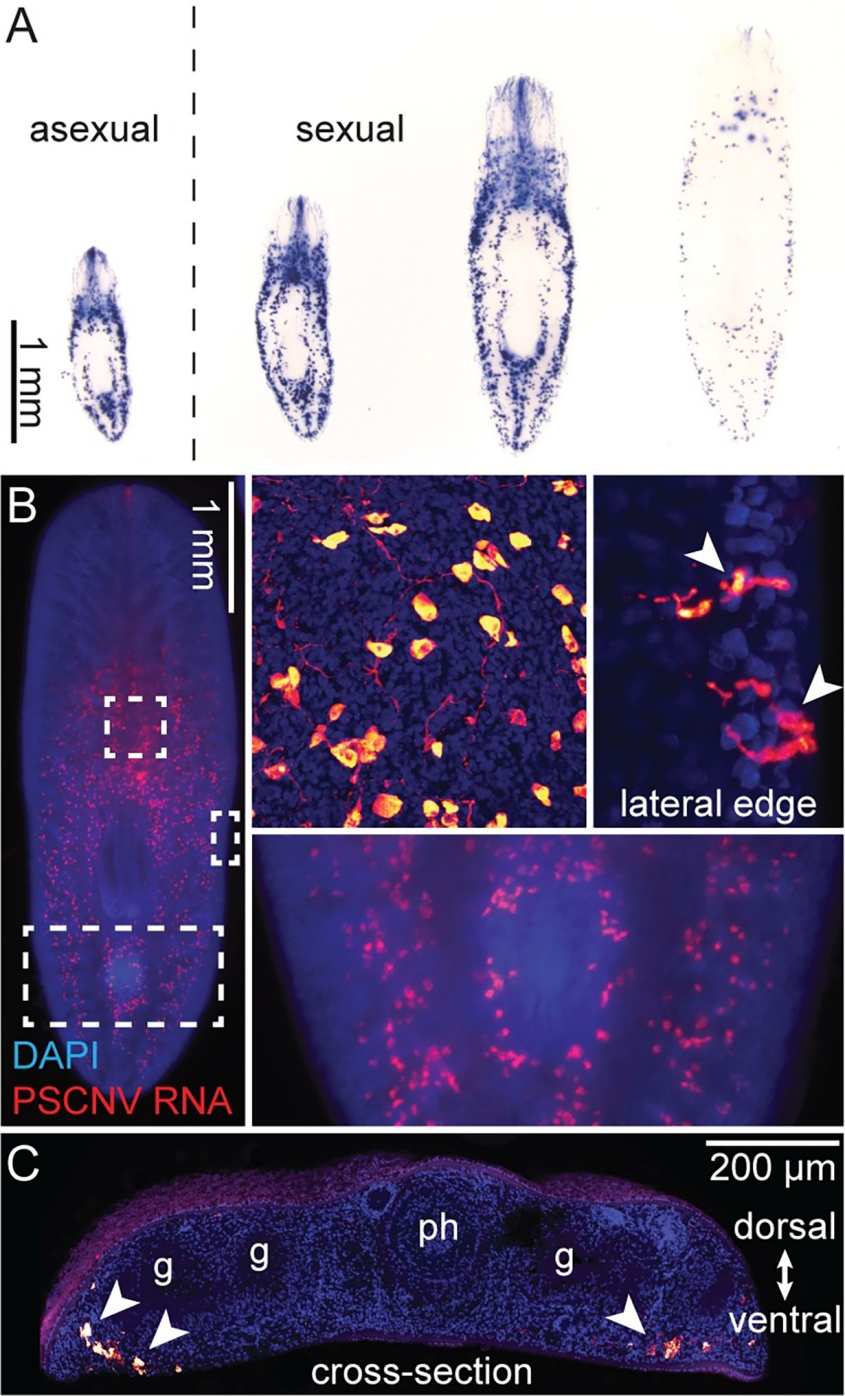
Expression of PSCNV RNA in planarians. (***A***) PSCNV RNA (blue) detected in asexual (left) and sexual *S*. *mediterranea* by whole-mount ISH. (***B***) Fluorescent ISH showing PSCNV expression in a sexual planarian. Insets show higher magnification of areas indicated by boxes. Top two insets are confocal projections. Secretory cell projections to lateral body edges are indicated by arrowheads. (***C***) Tiled confocal projections of PSCNV expression in a cross-section. Cells expressing PSCNV are ventrally located (arrowheads). Gut (“g”) and pharynx (“ph”) are indicated. DAPI (blue) labels nuclei.

We examined PSCNV infection in planarian tissues by whole-mount in situ hybridization (ISH). PSCNV RNA was detected abundantly in cells of the secretory system in both sexuals and asexuals (Fig 3A). Fluorescent ISH revealed viral RNA in gland cell projections that form secretory canals (Fig 3B). Notably, viral RNA was detected largely in ventral cells (Fig 3C) whose localization corresponds to mucus-secreting cells that produce the slime planarians use for gliding locomotion, and to immobilize prey [73].

**Fig 4:**
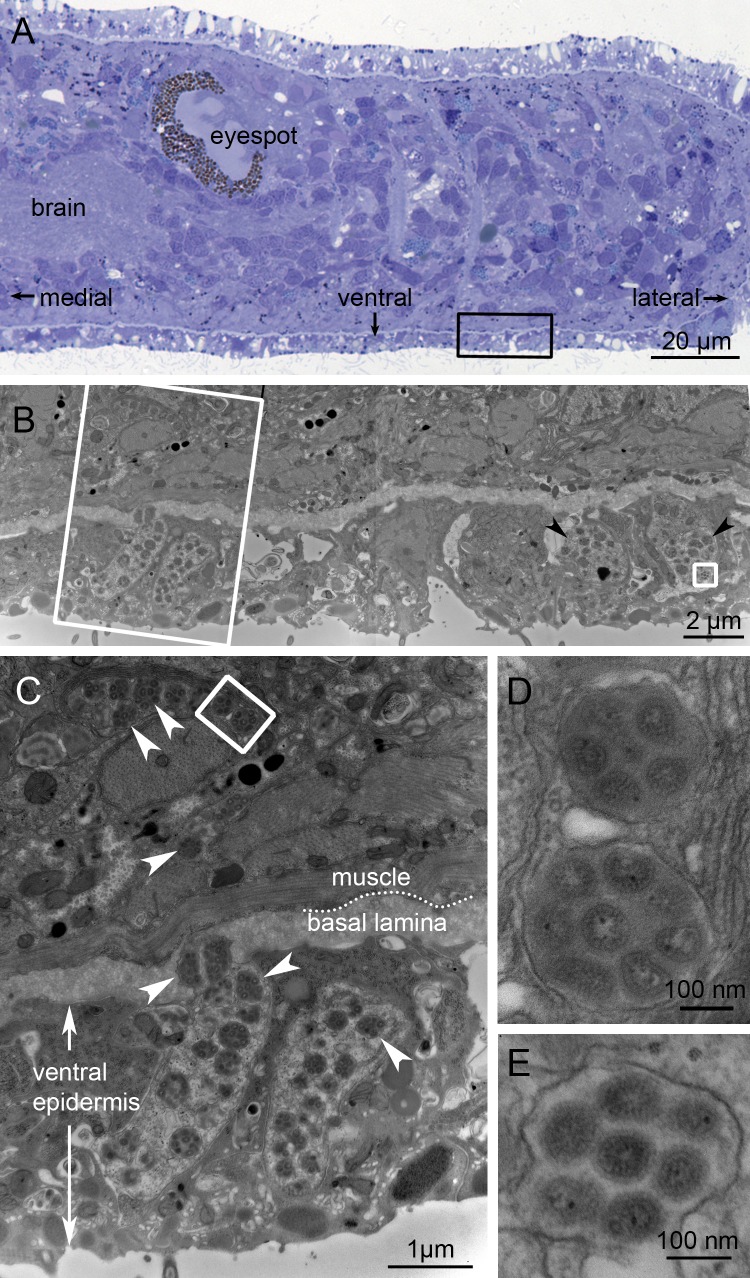
Putative PSCNV particles revealed by electron microscopy. (***A***) Adjacent histological transverse section, to orient EM images. Black rectangle corresponds to location of (***B***), a low-magnification EM view to provide context. White rectangle corresponds to location of (***C***), in which putative viral particles enclosed within membrane sacs are indicated by arrowheads. The white rectangle in (*C*) and square in (*B*) indicate positions of higher-magnification views shown in (***D***) and (***E***), respectively, each illustrating several viral particles within a membrane sac. In top-left of (*C*), note the mucus granules adjacent to virus-laden sacs (see also S2 Fig). Scale bars as indicated.

We then analyzed planarians by electron microscopy (EM) for the presence of viral structures. In one specimen, membrane-bound compartments containing 90–150 nm spherical-to-oblong particles resembling nidoviral nucleocapsids [74, 75] were found in the cytoplasm of mucus-secreting cells. These sub-epidermal gland cells are notable for their abundant rough endoplasmic reticulum and long projections into the ventral epithelium, through which they secrete mucus (S2 Fig). These cells provide an ideal environment for nidoviral replication, which co-opts host membranes to produce viral replication complexes [76, 77]. Putative viral particles were found both in deep regions of these cells, and in their trans-epidermal projections (Fig 4A–4C). The latter location suggests a route for viral transmission. Notably, particles in sub-epidermal layers have a “hazy” appearance and are embedded in a relatively electron-dense matrix (Fig 4D). In contrast, particles closer to the apical surface of the epidermis appear as relatively discrete structures, standing out against electron-lucent surrounding material (Fig 4E). The size, ultrastructure, and host-cell locations are all consistent with these structures being nidoviral nucleocapsids [74, 75].

In 280 images from the positive specimen, all other ultrastructural features were normal. Importantly, typical mucus vesicles were evident in this specimen, often immediately adjacent to vesicles containing putative virions (Fig 4C, see also S2 Fig). As such, we determined that these structures do not represent artefacts caused by atypical fixation of this specimen.

### Overview of the PSCNV proteome reveals a unique nidovirus

**Fig 5:**
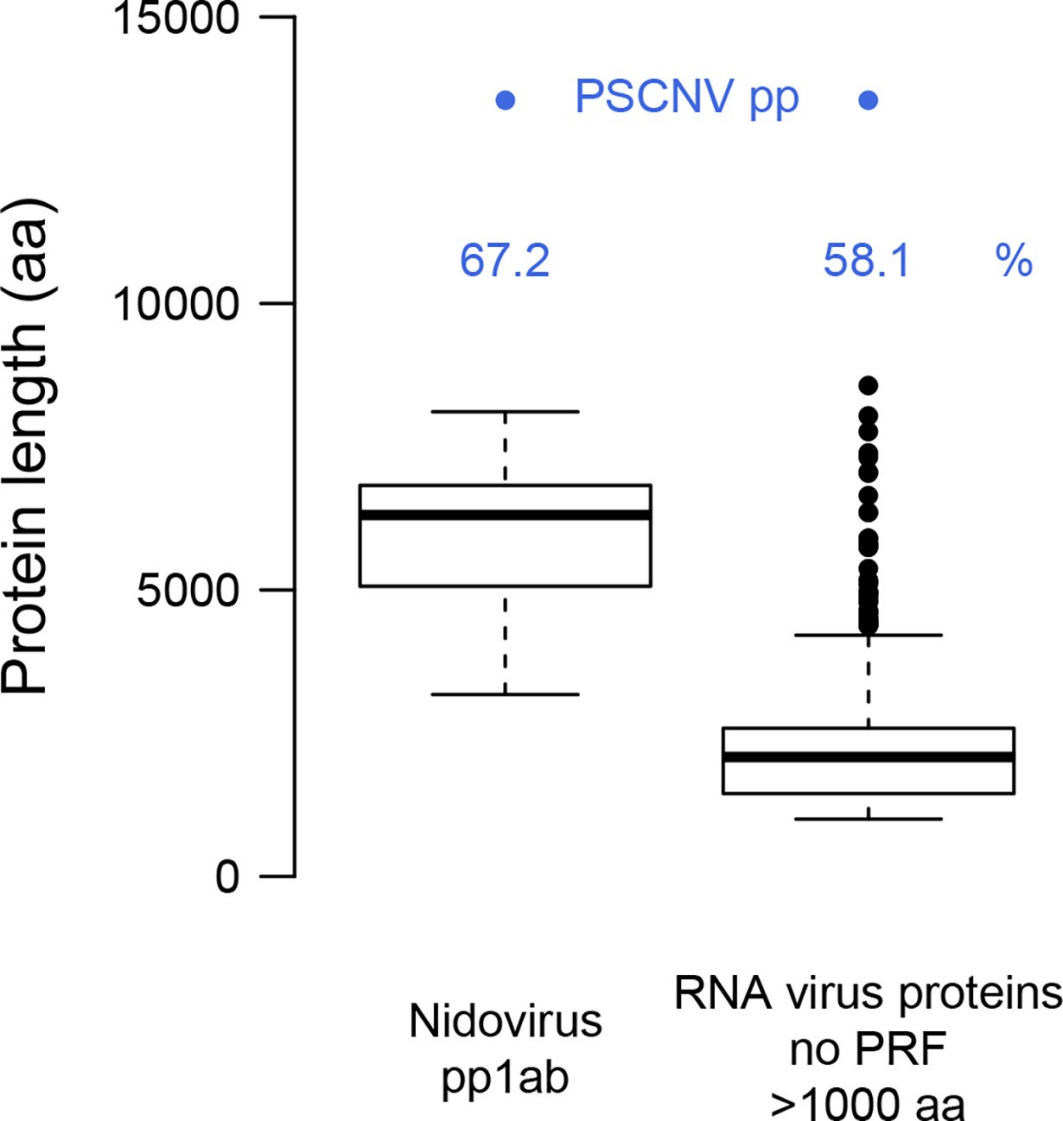
Largest proteins of nidoviruses and other RNA viruses in comparison with PSCNV polyprotein. Percentage indicates the difference between sizes of the PSCNV polyprotein (pp) and that of the next-largest entity. For details, see S1 Materials and Methods.

The genome and proteome of PSCNV are by far the largest yet reported for an RNA virus. Its RNA genome is ~25% larger than that of the next-largest known RNA virus (BPNV, [21]), which is separated by a comparable margin from the first nidovirus genome sequenced 30 years ago (IBV, [78]) (Fig 1A). The size of the predicted PSCNV polyprotein (13,556 amino acids, aa) is 58–67% larger than the largest known RNA virus proteins produced from a single ORF (8,572 aa; Gamboa mosquito virus, [79]) or multiple ORFs through frameshifting (8,108 aa; BPNV, [21]) (Fig 5).

Functional annotation of the PSCNV polyprotein by comparative genomics [14, 31, 80, 81] presented a distinct bioinformatics challenge, due to its weak similarity to other proteins and its extremely large size, which exceeds the average size of protein domains by approximately 75-fold. We delineated at least twenty domains in the PSCNV polyprotein, including twelve domains conserved in nidoviruses or other entities, using a multistage computational procedure that combined different analyses within a probabilistic framework (Fig 2; S3–S16 Fig; S5 Table; see Materials and Methods). We initially identified six regions highly enriched in hydrophobic residues characteristic of transmembrane domains, named TM1 to TM6 accordingly (Fig 2). The number and relative location of the TM domains resemble those found in the proteomes of nidoviruses, which commonly have five or more TM domains in non-structural and structural proteins [82–85]. We then identified fourteen regions enriched in individual amino acid residues (S4 Fig), with the strongest signal observed for Thr-rich region (residues 10429–10559, 44.3% Thr residues, up to 13.4 SD above the mean). Notably, the Thr-rich region overlaps with a Ser-rich region (10461–10501 aa, 19.5% Ser residues, up to 5.5 SD above the mean). Subsequently, two tandem repeats were identified toward the N-terminus of the polyprotein (residues 1616–1682 and 1686–1751, Probability 96.6%, S5 Fig), which showed no significant similarity to other proteins in the databases using HHsearch.

We used the domains described above to split the polyprotein into nine regions, which were analyzed by an iterative HHsearch-based procedure (outlined in S3 Fig and S1 Materials and Methods). Our approach identified eight domains that, together with TM2 and TM3, form a canonical synteny of replicative domains in the central part of the polyprotein (genome), which is characteristic of known invertebrate nidoviruses (Fig 2): 3CLpro, NiRAN, RdRp, ZBD, HEL1, ExoN, and S-adenosylmethionine (SAM)-dependent N7- and 2’-O-methyltransferases (N-MT and O-MT, respectively). Five of these domains (3CLpro, NiRAN, RdRp, HEL1, and O-MT) were identified by hits exceeding the 95% Probability threshold, while three others were based on weaker hits: 35.0% for ZBD, 39.1% for ExoN, and 80.8% for N-MT. Despite the lower Probability values obtained for the latter three domains, synteny and conservation of essential functional residues strongly suggest that they encode true homologs of canonical nidoviral proteins. Overall, the analysis demonstrates the existence of the three definitive nidoviral genomic subregions in the PSCNV single-ORF genome: ORF1a-, ORF1b-, and 3’ORFs-like. Within these regions, TM2, 3CLpro, and TM3 map to the ORF1a-like region, while NiRAN, RdRp, ZBD, HEL1, ExoN, N-MT, and O-MT map to the ORF1b-like region.

In addition to the canonical replicative domains present in the canonical order and location, we found four domains that are novel for nidoviruses: one upstream and three downstream of the array of the conserved replicative domains (S5 Table). These include a homolog of ribonuclease T2 (RNase T2, Probability 80.0%) upstream of the TM2, two fibronectin type II domains (FN2a and FN2b, 91.3% and 78.5%, respectively), and an ankyrin repeats domain (ANK, 98.9%) downstream of the O-MT. For the three domains identified with the under-threshold hits, additional support came from conservation of functionally important residues (see below).

We subsequently generated multiple sequence alignments (MSAs) of these domains for a representative set of established nidovirus species, followed by phylogenetic reconstruction to characterize PSCNV by revealing common and unique features of its conserved domains. The next three sections summarize the salient features of the replicative, novel, and structural domains of the polyprotein.

### Conserved and distinctive features in PSCNV’s replicative and regulatory proteins

#### 3CL protease (main protease of polyprotein processing)

Nidoviruses employ an ORF1a-encoded protease, 3CLpro, with a narrow substrate specificity that controls expression of ORF1a and ORF1b by releasing itself and downstream domains comprising replicative machinery, up to and including the most C-terminal domain encoded by ORF1b [42]. This protease includes a catalytic domain composed of a two-barrel chymotrypsin-like fold and a C-terminal accessory domain whose fold varies among nidoviruses [86, 87]. It is flanked by two TM domains in the polyprotein (TM2 and TM3), which anchor the RTC to the membrane [43] (Fig 2). The catalytic domain of PSCNV 3CLpro was identified in the canonical position between TM2 and TM3 (S3 Fig) through hits to hidden Markov model (HMM) profiles of cellular serine proteases with chymotrypsin-like folds, while its similarity to the HMM profile of the nidovirus 3CLpro was extremely low (Probability 2.8%; see S5 Table), indicating unique properties. The long distance (~250 aa) between the C-terminus of the putative catalytic domain of PSCNV 3CLpro and the N-terminus of TM3, suggests that PSCNV 3CLpro possesses a highly divergent C-terminal domain. Unlike other characterized invertebrate nidoviruses, which all employ cysteine as the catalytic nucleophile [88, 89], PSCNV 3CLpro appears to use the Ser-His-Asp catalytic triad typical of cellular chymotrypsin-like proteases (S7 Fig). PSCNV 3CLpro was also found to have a residue variation that has never been observed in 3CLpro-encoding viruses before: it encodes a Val residue in the position commonly occupied by a His residue in the putative substrate-binding pocket (GX**V** vs G/YX**H**, highlighted in bold) [42, 88–91].

#### NiRAN, RdRp, ZBD, HEL1 (RNA replicative enzyme domains)

Consistent with the essential enzymatic activities of RdRp (the catalytic domain of RNA polymerase) and HEL1 (helicase), the PSCNV polyprotein hits to HMM profiles of these domains were ranked as the top two by two measures of statistical significance (S5 Table). Mutiple sequence alignments confirm the high conservation of canonical motifs and residues in these domains (S9 and S11 Figs). The only exception concerns the RdRp C motif: a Ser residue of the nidovirus-specific SDD signature [23] is replaced by Gly in PSCNV. As in previously described nidoviruses, PSCNV’s HEL1-associated ZBD includes 12 Cys or His residues that are homologous to putative Zn-binding residues (S10 Fig). The PSCNV RdRp-associated NiRAN retains six out of the seven invariant residues observed in all known nidoviruses [45] (S8 Fig). The outlier is in motif B_N_, in which Thr takes the place of an invariant Asp as the distal residue. In addition, the B_N_ motif in PSCNV also contains an Asn at a highly conserved Ser/Thr position. These substitutions might represent the “swapping” of the two residues, assuming that the chemically similar Asp and Asn residues play an equivalent role in the respective proteins. This hypothesis is plausible, given that the two affected residues are expected to be in close proximity to each other, separated only by an incomplete turn of the putative alpha-helix of the motif B_N_ (S8 Fig). Another notable feature of the PSCNV NiRAN is the large distance between invariant Lys and Glu residues of the motif A_N_: 20 aa in PSCNV compared to 5–9 aa in other nidoviruses. The conservation of NiRAN and ZBD in PSCNV is significant for assignment of this virus to the nidoviruses, since both domains are the only known genetic markers of the order *Nidovirales*.

#### ExoN, N-MT, O-MT (proofreading and RNA-modifying enzyme domains)

ExoN is a 3’-5’ exoribonuclease that improves the fidelity of replication and transcription by excision of a 3’ mismatched nucleotide in characterized nidoviruses [31–34, 92–94]. Like its orthologs, the PSCNV ExoN contains the characteristic D-E-D-H-D pentad, which includes counterparts of catalytic and other active site residues. The H-D subset is embedded within a highly conserved domain, whose structure is maintained by two Cys and two His residues coordinating a Zn^2+^ in characterized nidoviruses. However, these residues are substituted in PSCNV (H-C-H-C by E-S-Q-Q), which may therefore lack this Zn-finger (S12 Fig). In this respect, PSCNV ExoN is more similar to its cellular homologs than to those of nidoviruses (S5 Table). In contrast, the ExoNs of all ExoN-positive nidoviruses, including PSCNV, include another (upstream) Zn-finger, which distinguishes them from related enzymes of other origins. The N-MT and O-MT are implicated in viral RNA capping machinery [31, 92, 95–100]. In both transferases, a number of residues crucial for substrate and ligand binding are conserved in PSCNV homologs, including Zn-binding residues of N-MT (S13 Fig), and the catalytic K-D-K-E tetrad of O-MT (S14 Fig). Notably, like ExoN, O-MT is conserved in all nidoviruses with genomes >20 kb.

### PSCNV encodes protein domains that are novel to nidoviruses

#### RNase T2

The PSCNV RNase T2 homolog was identified upstream of the TM2 domain. It conserves both active-site motifs typical of such RNases, CASI and CASII, including catalytic His, Glu, and Lys residues, (S6 Fig) suggesting an enzymatically active protein [101].

#### Fibronectin type II (FN2) domains

We identified two FN2 domains, FN2a and FN2b, with only 21.7% pairwise identity to each other, including few residues aside from the most conserved Cys and aromatic residues (S15 Fig). According to the *Schmidtea mediterranea* genome database (SmedGD; [102]), several proteins of *S*. *mediterranea* include putative FN2 domains, but neither these nor FN2 domains of other origins show particular sequence affinity to those of PSCNV. Thus, the historical acquisition and subsequent evolution of these domains is unclear at this time.

#### Ankyrins

**Fig 6:**
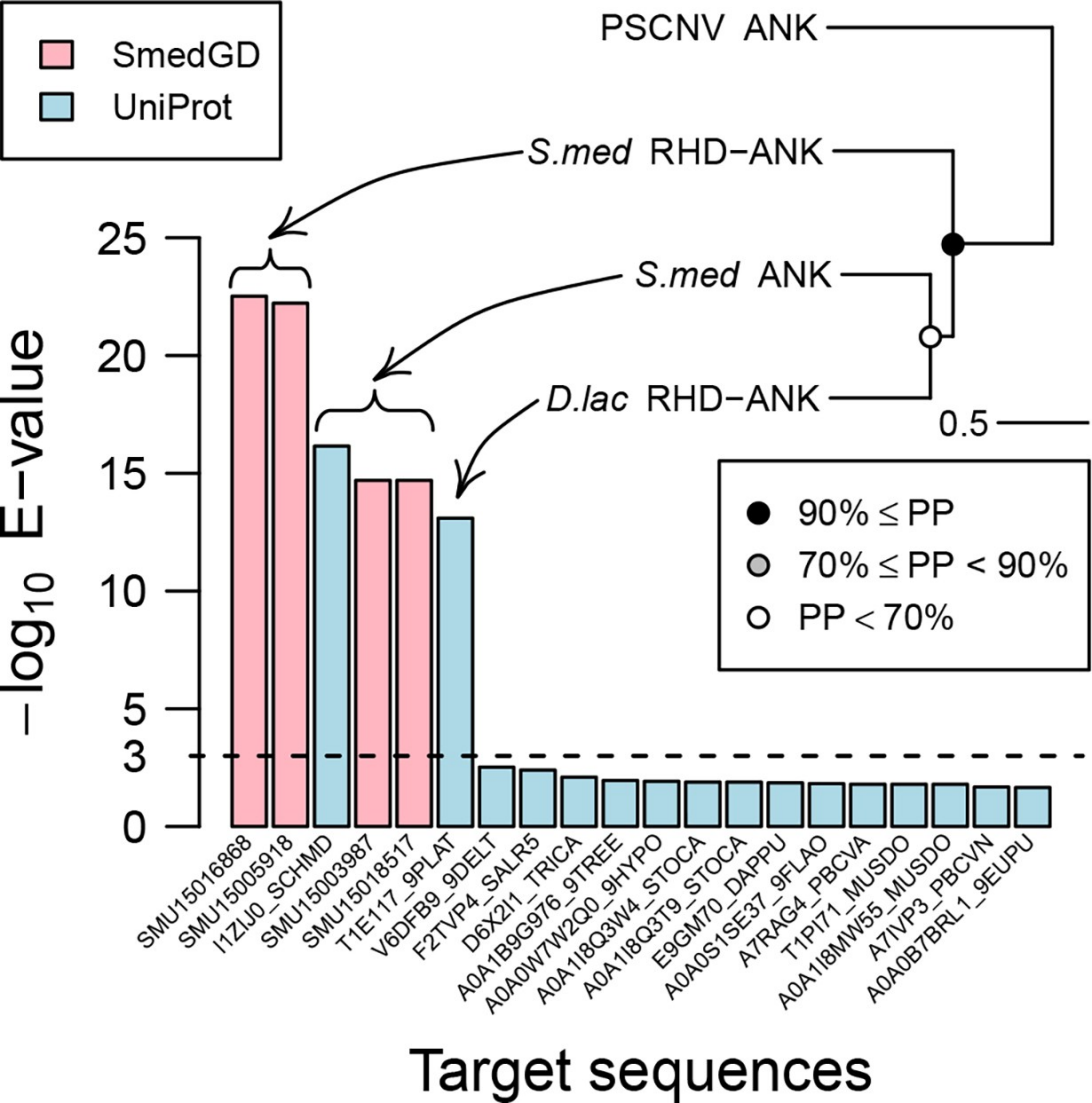
ANK domain of PSCNV and its homologs. The closest cellular homologs of PSCNV ANK are ranked by similarity (left, above the broken baseline) and depicted through phylogeny (right; reconstructed and rooted by BEAST, summarized as maximum clade credibility tree; PP, posterior probability of clades) along with protein domain architecture: *S*. *med*, *Schmidtea mediterranea*; *D*. *lac*, *Dendrocoelum lacteum*; RHD, Rel homology DNA-binding domain.

We identified three divergent ankyrin repeats in a PSCNV polyprotein region of ~100 aa (S16 Fig). In searches of Uniprot and the host proteome (Smed Unigene) using BLAST, the PSCNV ANK domain yielded highly significant hits (E-values ranging from 3E-23 to 8E-14, Fig 6) to proteins from *S*. *mediterranea* and another free-living planarian, *Dendrocoelum lacteum* [103]. The cellular domains clustered together in a phylogenetic reconstruction of the evolutionary relationship between these proteins and the PSCNV ANK using BEAST software (LG+G4 model, relaxed clock with uncorrelated log-normal rate distribution) (Fig 6). The topology of this tree implies that an ancestor of PSCNV acquired a host ANK domain prior to the divergence of the *S*. *mediterranea* and *D*. *lacteum* lineages, but we cannot exclude an alternative explanation in which viral ANK repeats experienced accelerated evolution compared to host sequences that was not evident in our analysis.

### Putative structural proteins of PSCNV

The 3’ORFs region of nidoviruses encodes components of the enveloped virion [23, 54], which define receptor specificity [55–57] and typically include the nucleocapsid protein (N), characterized by biased amino acid composition and structurally disordered region(s) [104, 105], spike glycoprotein(s) (S protein in corona- and toroviruses) and transmembrane matrix protein (M in corona- and toroviruses) enriched with TM regions [58, 59, 106]. As expected from the weak sequence conservation of this region in other nidoviruses [14, 107] and its weak similarity with other viruses [108], we were unable to find statistically significant similarity between the PSCNV polyprotein and structural proteins of the known nidoviruses. Nevertheless, important nidoviral themes are evident.

First we noted that the genome distribution of the TM-encoding regions in PSCNV conformed to that observed in other nidoviruses, with TM1 and TM2 located upstream of 3CLpro, TM3 C-terminal to 3CLpro, and TM4–TM6 downstream, in the 3’ORFs-like region (Fig 2). In nidoviruses, the TM domains encoded in the 3’-genome region are known to be part of the S and M proteins or their equivalents, and occasionally additional accessory proteins [14, 58, 59, 106, 109]. The extracellular portion of the S protein is supported by multiple disulfide bridges between conserved Cys residues [56]. In PSCNV, a Cys-rich region was observed downstream of TM5 (S4 Fig). In an approximately 650 aa region surrounding the TM6 domain (4.7% of the polyprotein length), we identified six areas enriched in Pro, Leu, Gly, Gln, Asn, or Arg, in close proximity to each other (S4 Fig). This region accounted for 43% of all residue-enriched areas in the polyprotein; such an exceptionally high concentration of sequences enriched with specific amino acids is indicative of unusual properties. Accordingly, this area was predicted to include the longest stretch of disordered regions. In nidoviruses, disordered hydrophilic-rich areas are characteristic of N proteins.

In PSCNV, the polyprotein region downstream of O-MT is ~4000 aa, more than twice as large as the largest known structural protein of nidoviruses [106]. We reasoned that this part of its polyprotein might be processed by cellular signal peptidase (SPase) and/or furin to produce several proteins, as documented for maturation of the structural proteins of many RNA viruses, including nidoviruses [110–114]. Indeed, our analysis of potential cleavage sites of these proteases revealed highly uneven distributions (S4 Fig), with sites predicted only in the N- and C-terminal parts of the polyprotein: 1400–3100 aa (one SPase and four furin sites) and 10200–13200 aa (three SPase and five furin sites). All of these are outside of the region that must be processed by 3CLpro. With the exception of the most C-terminal furin site, all predicted sites are in close vicinity to provisional borders of the domains described above, as would be expected if these domains function as distinct proteins. Specifically, if the predicted SPase and furin sites are cleaved, TM1, TM4, TM5, and TM6 would end up in separate proteins, with one protein including the TM4 and ANK domains. With predicted cleavage sites flanking it from both sides, TM5 may be released as a separate protein, most similar to M proteins in size and hydrophobicity. We also note that two putative proteins may combine a FN2 module with a disordered region: FN2a with a Thr/Ser-rich region and FN2b with the Pro/Leu/Gly/Gln/Asn/Arg-rich region, respectively. Based on the reasoning outlined above, the latter combination may constitute a region of the N protein.

Overall, our analysis of the predicted PSCNV proteins suggests that its genome is functionally organized in much the same manner as in the multi-ORF nidoviruses: with the non-structural and structural proteins encoded in the 5’- and 3’- regions, respectively.

### PSCNV clusters with invertebrate nidoviruses in phylogenetic analyses

Next we sought to determine when PSCNV’s lineage emerged, relative to other nidoviruses. The proteome analysis described above indicates that PSCNV shares the main features characteristic of invertebrate nidoviruses, although it also exhibits distinctive properties indicative of a distant relationship with previously characterized nidoviruses. To resolve very deep branching, we used an outgroup in our analysis, and selected astroviruses for this purpose [23]. Astroviruses [115] and nidoviruses share multi-ORF genome organization, a central role for 3CLpro in polyprotein processing, and similarities in the RdRp domain. Conversely, astroviruses do not encode a HEL1, NiRAN or ZBD, and their 3CLpro is highly divergent. Given the divergent 3CLpro of PSCNV, RdRp remained as the only domain most suitable for phylogeny reconstruction; this domain has been used in many studies on macroevolution of nidoviruses [21, 23, 35, 116].

**Fig 7:**
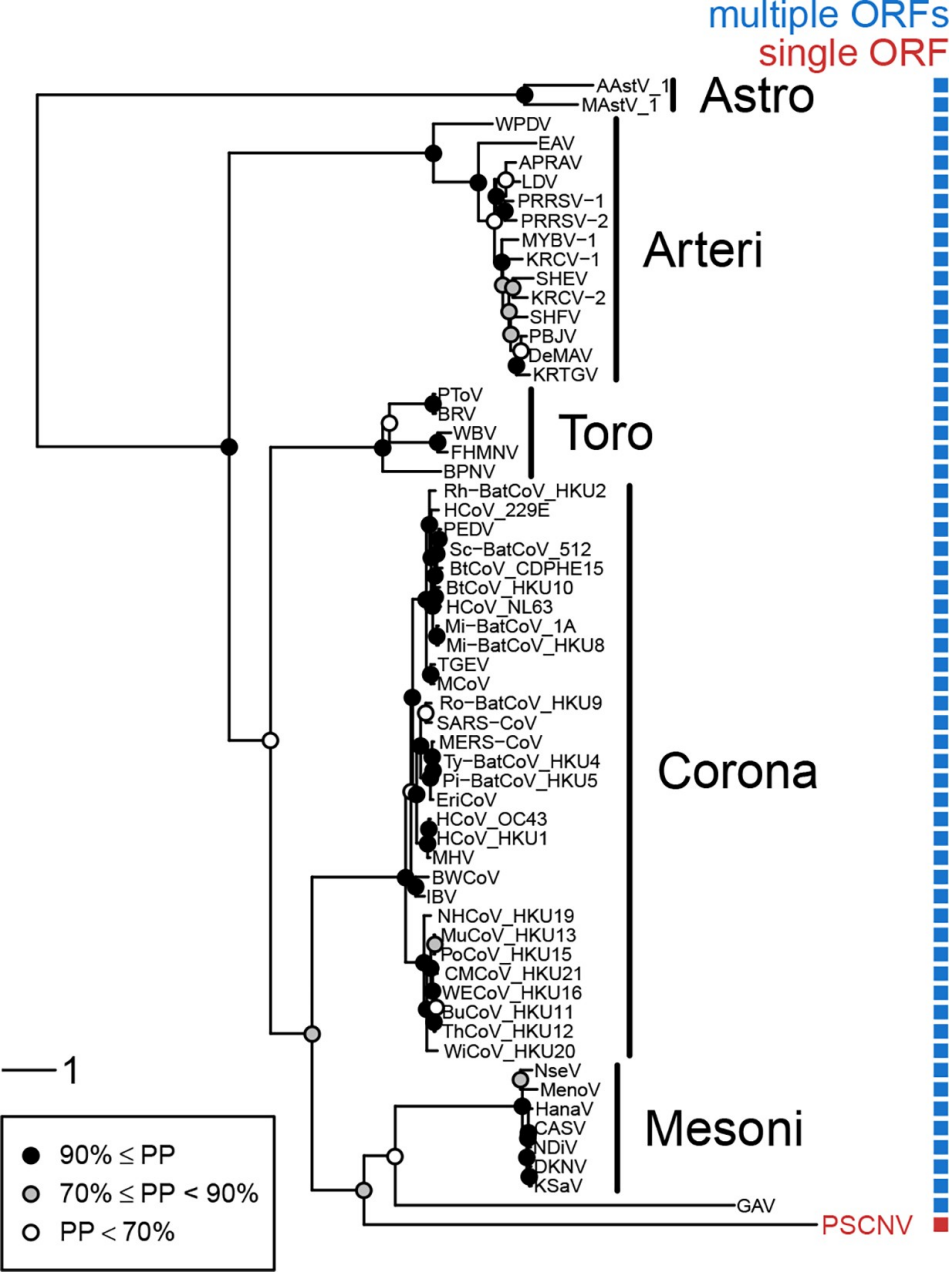
Phylogeny of PSCNV. RdRp-based Bayesian maximum clade credibility tree and the genomic ORF organization (character state) for PSCNV, a representative set of nidoviruses, and astroviruses (outgroup). PP, posterior probability of clades. For virus names, see S1 Table.

We performed phylogenetic analysis of the RdRp core region by Bayesian inference (BEAST software, LG+I+G4 model, relaxed clock with uncorrelated log-normal rate distribution). Nidoviruses including PSCNV formed a monophyletic group in >90% of the trees in the analyzed Bayesian sample, with PSCNV being one of the basal branches in the cluster of invertebrate nidoviruses in 88.7% of the trees, basal to either mesoni- and roniviruses (54.7% of the trees), or roniviruses (20.6%), or mesoniviruses (13.4%) (Fig 7 and S17 Fig).

In addition, we built a nidovirus phylogeny without an outgroup (BEAST software, LG+I+G4 model, relaxed clock with uncorrelated log-normal rate distribution), based on a concatenated alignment of five domains conserved in all nidoviruses (3CLpro, NiRAN, RdRp, ZDB, HEL1). Again, PSCNV belonged to the cluster of invertebrate nidoviruses in the majority of trees and was basal to either mesoni- and roniviruses (11.8% of the trees), or roniviruses (83.0%), or mesoniviruses (3.6%).

### Origin of single-ORF genome organization

Is the unique single-ORF genomic organization of PSCNV an ancestral characteristic of nidoviruses, or has it evolved from an ancestral multi-ORF organization? To choose between these alternative scenarios, we need to reconstruct a genomic ORF organization of the most recent common ancestor (MRCA) of nidoviruses. Such reconstruction by orthology, which was used for RdRp-based phylogeny, is not feasible with the current dataset, as none of the open reading frames or their overlaps (with the exception of the ORF1a/ORF1b junction) are conserved in all known multi-ORF nidoviruses.

To address this challenge, we noted that nidoviruses with multi-ORF organization, unlike PSCNV, recurrently use initiation and termination codons to delimit ORF-specific proteins in the 3’ORFs region, indicative of pervasive selection forces that operate in all recognized nidovirus species. Therefore, we reasoned that multi- and single-ORF organizations in nidoviruses could be treated as two alternative discrete states of a single trait (ORF organization), regardless of the complexity of their actual evolutionary relations in the 3’ORFs region and assuming the rate of transition between any two multi-ORF organizations to be extremely high compared to that between single- and multi-ORF organizations. This reasoning allows us to reformulate the question in the framework of ancestral state reconstruction analysis: if each extant nidovirus is characterized by one of the two states of a trait (ORF organization), which state of the trait existed in their MRCA?

To conduct this analysis, we applied the BayesTraits [117] program to the RdRp-based Bayesian sample of phylogenetic trees including the outgroup, which accounts for uncertainty in the phylogeny inference of nidoviruses. The results strongly favored multi-ORF organization of the ancestral nidovirus (Log Bayes Factor (BF) 6.06 and 6.16, when multi-ORF genome organization, or no information about genome organization, were specified as states of the trait for astroviruses, respectively) (S17 Fig). Similarly, strong support (Log BF 4.79) for multi-ORF ancestral organization was obtained when the analysis was conducted based on a phylogeny without an outgroup, reconstructed using five nidovirus-wide conserved domains.

### PSCNV expanded disproportionately in the ORF1b-like region

**Fig 8:**
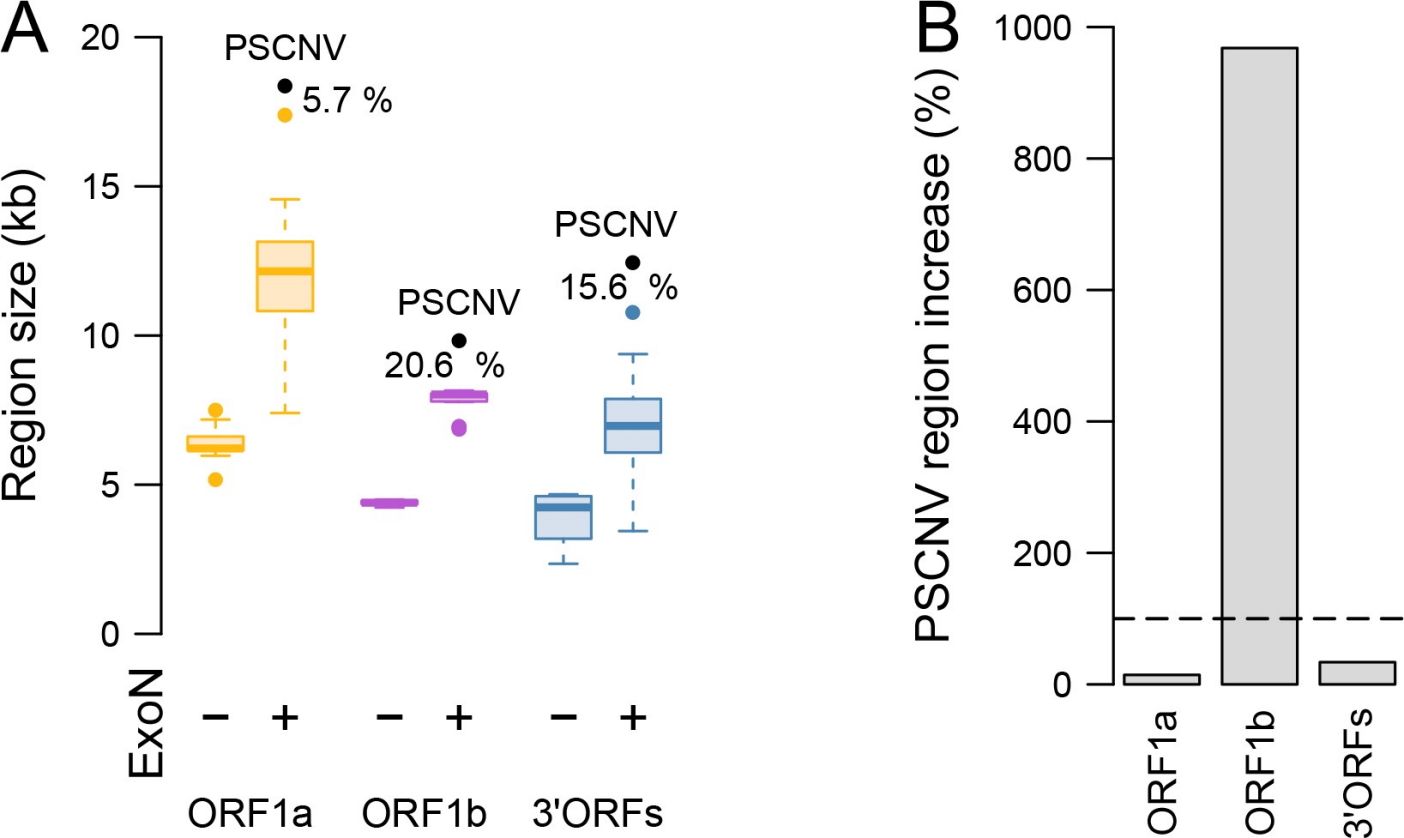
Nidovirus genome and region size differences. (***A***) Sizes of three nidovirus ORF regions. Percentage indicates the difference between a genome region’s size in PSCNV, and that of the next-largest entity. Color scheme as in Fig 2. (***B***) Size increase of the three genome regions in PSCNV (grey bars) relative to the increase expected if all regions had expanded evenly (broken line); calculated using formula D_3_, see text and S6 Table.

Each of the three main regions of the PSCNV genome is larger than its counterparts in all other nidoviruses (Fig 8A, S1 and S6 Tables). However, the size differences between PSCNV and the next largest nidovirus in each of these regions are smaller than those observed for complete genomes (Fig 8A: 5.7%, 20.6% and 15.6% for ORF1a, ORF1b and 3’ORFs, respectively, vs 22.9% for the genome). This paradoxical observation is due to profound differences in regional size variation among nidoviruses [66] such that different nidoviruses are the next largest to PSCNV for each of the three main regions (S1 Table).

To account for these and other differences in sizes of the three regions while assessing the regional size increases of PSCNV, we employed two measures in addition to the percentage size increase between PSCNV and the next largest nidovirus (see Materials and Methods, formulas D_2_ and D_3_ versus formula D_1_). First, for each genome region, we normalized the size difference between PSCNV and the next largest virus against the difference between the latter and the median-sized virus for that region (formula D_2_). Second, we checked how much the deviation calculated with formula D_2_ differs from that expected under a hypothesis that size changes are uniform across the three genome regions, and therefore proportional to genome-wide changes (formula D_3_). These measures show that, relative to the size variation among known ExoN-positive nidoviruses, the size increase in the ORF1b region was extraordinarily large (D_2_ = 1270.5% and D_3_ = 968.1%), while the corresponding increases in the two other regions were modest and smaller than could be expected (18.9% and 14.4% for ORF1a, and 44.3% and 33.7% for 3’ORFs) (Fig 8B, S6 Table).

### PSCNV genome features suggest mechanisms to regulate the stoichiometry of proteins encoded by a single-ORF genome

Virus reproduction requires different viral protein stoichiometries at distinct replicative cycle stages, a challenge for a single-ORF genome theoretically producing equimolar quantities of encoded polypeptides. To this end, all previously described nidoviruses employ -1 PRF to translate ORF1a+ORF1b, in addition to ORF1a alone, to produce two polyproteins from a genomic template: pp1ab and pp1a, respectively [40, 41]. The net result of this mechanism is relatively high expression of the ORF1a- compared to ORF1b-encoded proteins, since PRF occurs at the ORF1a/1b junction in 15–60% of ORF1a translation events. In contrast, proteins encoded in the 3’ORFs region are produced by translation of subgenomic (sg) mRNAs, synthesized on specific minus-strand templates [51–53], which are in turn produced by discontinuous RNA synthesis on genomic templates. Discontinuous minus-strand template synthesis relies on lTRS and bTRS, which are nearly identical, short repeats at sites where RNA synthesis pauses (upstream of 3’ORFs) and resumes (in the 5’-UTR), respectively. Templates of some sg mRNAs may be terminated at bTRS. Both transcription and translation of sg mRNAs provide a means to produce relatively large quantities of structural proteins, compared to non-structural (replicative) proteins, late in the replicative cycle, and to regulate production of accessory proteins. We analysed the PSCNV genome for evidence of such mechanisms.

#### Genome translation and frameshifting

**Fig 9:**
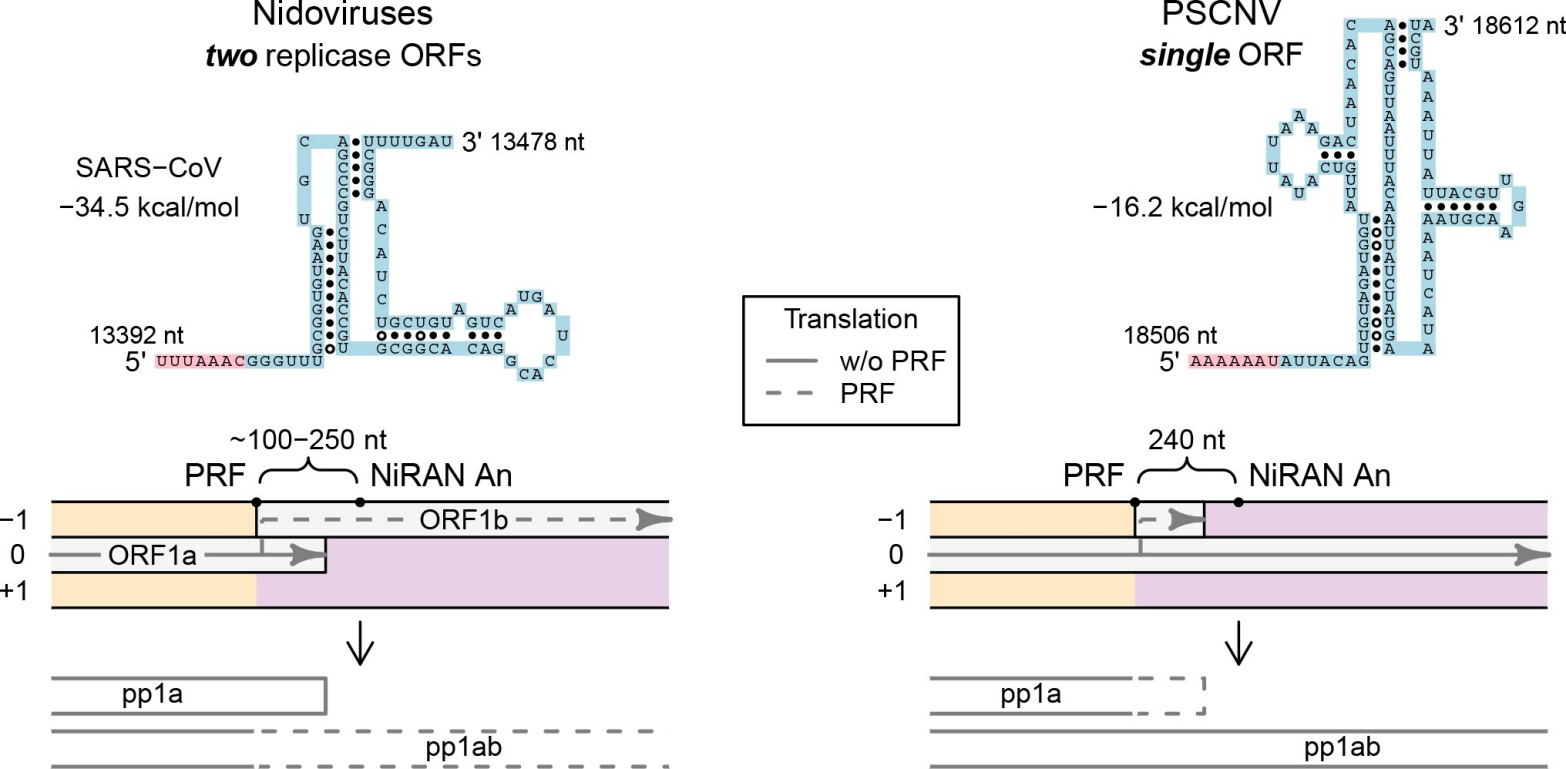
Genome translation. Comparison of mechanisms by which ORFs 1a and 1b are translated in previously described nidoviruses (left) and PSCNV (right, hypothetical). On the top, RNA structure of the PRF sites, predicted by KnotInFrame, is presented: slippery sequence, pink; pseudoknot, blue.

ORF1a/1b -1 PRF in nidoviruses is facilitated by a pseudoknot preceded by a slippery sequence, which lies ~100–250 nt upstream of the region encoding the A_N_ motif of the NiRAN domain. To check whether an analogous structure is present in the PSCNV genome, KnotInFrame was applied to the 1000-nt genome fragment immediately upstream of the region encoding the NiRAN A_N_ motif. The top prediction identified nucleotide 18512 as a putative PRF site. This nucleotide is positioned 240 nt upstream of the region encoding the NiRAN A_N_ motif, and the free energy of the downstream pseudoknot is -16.2 kcal/mol (Fig 9, right). Notably, when the identical procedure was applied to SARS-CoV, the top prediction (Fig 9, left) correctly identified the experimentally verified PRF site with only minor deviations between the predicted and experimentally verified structure of the downstream pseudoknot [118]. As a result of -1 PRF at the identified PSCNV site, translation would shift from the main PSCNV ORF to a small 39-nt ORF. If -1 PRF at this site indeed occurs in a fraction of translation events of the ORF1a-like region, translation of the ORF1b-like region (and also 3’ORFs-like region) will be attenuated, with a net result that should be similar to that of other nidoviruses: proteins encoded in the ORF1a-like region will be expressed in higher quantities than proteins encoded in the ORF1b-like region.

#### Discontinuous genome synthesis (transcription)

**Fig 10:**
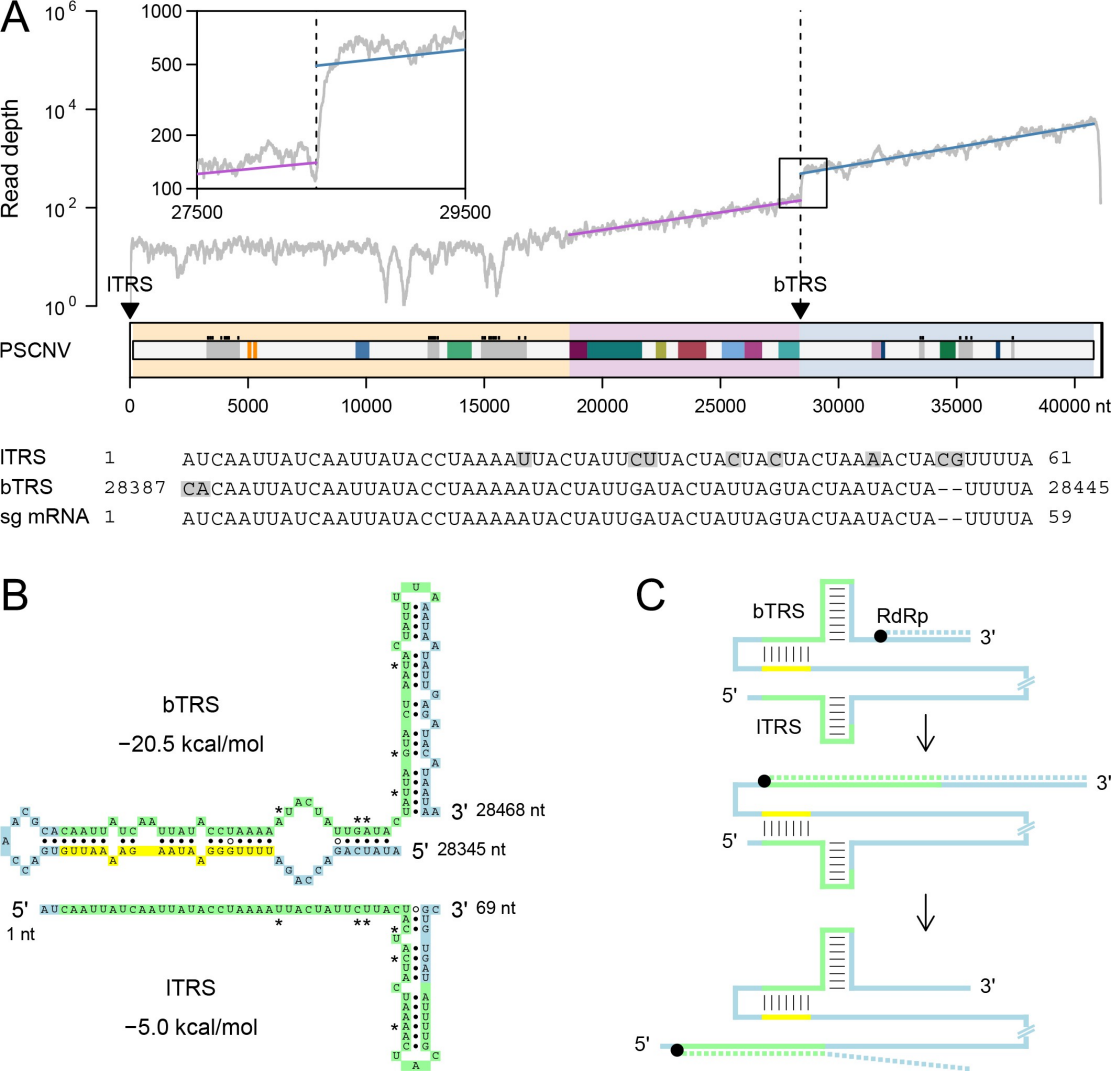
Genome transcription. (***A***) Mean depth of RNA-seq coverage along the PSCNV genome (approximated by exponential regression in ORF1b-like and 3’ORFs-like regions) calculated based on five datasets used to assemble the transcriptomes in which PSCNV was found [67]. Indicated on the genome map (colored as in Fig 2) are the positions of oligonucleotide repeats (leader and body TRSs) in the genome, and below is their alignment with a sg mRNA 5’-terminus identified by 5’-RACE (nucleotide mismatches between sg mRNA and TRSs are shown with grey backgrounds). (***B***) Predicted secondary structure of TRSs. TRSs are highlighted in green, region upstream of bTRS, interacting with its 5’-terminus–in yellow, asterisks indicate mismatching nucleotides of TRSs. (***C***) Model of discontinuous RNA synthesis mediated by TRSs and their secondary structure. The genome is represented by a solid line, and the nascent minus strand by a dashed line. Color code matches that of panel *B*.

To search for TRSs in the PSCNV genome, its 5’-UTR was compared with the whole genome sequence using nucleotide BLAST. A pair of highly similar sequences (86% identity, E-value 2E-14) was identified in the 5’-UTR (3–61 nt) and immediately upstream of the 3’ORFs-like region (28389–28445 nt) (Fig 10A). If these repeats are indeed utilized as TRSs in discontinuous RNA synthesis, a template for a 12717 nt sg mRNA (excluding the polyA tail) would be produced. Indeed, we observed a ~3x rise in transcriptomic read coverage beginning at the bTRS genome position, and confirmed the presence of the expected template-switching junction in a sg RNA by 5’-RACE conducted on infected planarians (Fig 10A). That sg mRNA contains a 12327 nt ORF identical to the 3’-terminus of the main PSCNV ORF (28473–40799 nt in genome coordinates), if its translation starts from the 5’-most Met codon of the sg mRNA.

To explore a mechanistic basis for RNA strand translocation during the postulated discontinuous transcription, we predicted RNA secondary structure for the PSCNV genome in the vicinity of the TRS signals (Fig 10B). According to the prediction, 3’-terminal nucleotides of both TRSs, starting from the 36th TRS nucleotide, form hairpins involving nucleotides of the downstream region. In contrast, 5’-terminal parts of the TRSs may be folded differently: the first 35 nucleotides of the lTRS remain unstructured, while the first 35 nucleotides of the bTRS form a hairpin involving the upstream sequence. Two parts, tip and basal, could be recognized in this hairpin. The tip part includes 22 nucleotides of bTRS that seems to form 17 canonical base pairs with a genome region just 11 nucleotides upstream (yellow in Fig 10B). Since these 22 nucleotides of bTRS are identical to those of the lTRS, the latter might alternatively form a stable secondary structure with the yellow region (upstream of bTRS; Fig 10C). The basal part of the hairpin is much smaller and may not be conserved in the possible interaction involving lTRS.

### Identification of partial genome sequences of putative planarian viruses related to PSCNV

Finally, we used the PSCNV polyprotein as a query sequence to survey several flatworm species’ transcriptomes in the PlanMine database [119] for the presence of other nidoviruses related to PSCNV. We identified six contig sequences with highly significant similarity to PSCNV indicative of at least two nidoviruses (S18 Fig). These contigs originate from transcriptomes of *S*. *mediterranea* (uc_Smed_v2 and ox_Smed_v2 assemblies, two and one contigs, respectively; the latter contig was excluded from consideration due to being almost identical to one of the former contigs) and another planarian species, *Planaria torva* (dd_Ptor_v3 assembly, three contigs). Translations of the two uc_Smed_v2 contigs of 814 nt and 1839 nt gave hits of >99% aa identity to the very C-terminus of PSCNV polyprotein, indicative of a variant of PSCNV circulating in the same host species (see section above). In contrast, the dd_Ptor_v3 transcriptome included two short contigs (283 nt and 289 nt) with hits to the PSCNV RdRp domain (38 and 48% aa identity) as well as an 8811-nt contig, whose translation in the +1 frame gave 3 discontinuous hits, one to the O-MT domain of the ORF1b-like region (37% aa identity) and two to the 3’ORFs-like region and its FN2b domain (25% and 37% aa identity). These domains are separated by different distances in PSCNV and the 8811-nt contig. It is notable that all three hits from the *P*. *torva* contig correspond to its translation in the same frame, uninterrupted by stop-codons, suggesting that ORF1b-like and 3’ORFs-like regions of this putative and divergent virus could also be expressed from a single ORF.

## Discussion

The advent of metagenomics and transcriptomics has greatly accelerated the pace of virus discovery, leading to studies reporting genome sequences of dozens to thousands of new RNA viruses in poorly characterized hosts [35, 36, 79, 120–126]. These developments have substantially advanced our appreciation of RNA virus diversity, and improved our understanding of the mechanisms of its generation [127, 128]. Notwithstanding that sea change, the largest known RNA genomes continue to belong to nidoviruses, as has been the case for 30 years, since the first coronavirus genome of 27 kb was sequenced [14, 21, 78] (Fig 1A).

This study’s transcriptomics-based discovery of PSCNV in planarians reinforces the status of nidoviruses as relative giants among RNA viruses, and also demonstrates that RNA genomes may be substantially larger than previously understood. The discovery of a virus with this large 41.1-kb RNA genome was unexpected in the context of accumulating genomic data on viruses and emerging concepts in the field. Below, we discuss the implications of PSCNV’s distinctive features, and future directions of research.

### PSCNV is distantly related to previously described nidoviruses

The PSCNV polyprotein includes distant homologs of all ten domains common to invertebrate nidoviruses, as well as the vertebrate *Coronavirinae* subfamily [14, 45]. These were identified with high statistical confidence, using an iterative bioinformatics procedure with profile searches at its core. These domains include the definitive nidovirus markers NiRAN and ZBD, and all ten are syntenic between PSCNV and other nidoviruses. Most are located in the ORF1b-like (replicase) region, which also includes four subregions left unannotated (Fig 2). Of these unannotated subregions, one flanked by ZBD and HEL1 may correspond to the regulatory domain 1B, which is uniformly present but poorly conserved in helicases of nidoviruses [48, 49], while the other three may represent domains uniquely acquired by a PSCNV ancestor. Like all characterized invertebrate nidoviruses, but unlike most vertebrate nidoviruses [14, 129], PSCNV does not encode a homolog of an uridylate-specific endonuclease (NendoU) [31]. Accordingly, our rooted RdRp-based phylogenetic analysis assigned PSCNV to a monophyletic clade of invertebrate nidoviruses. Another topologically similar tree was inferred using five nidovirus-wide conserved domains with a dataset that did not include an outgroup. The observed tree topology is also broadly compatible with other observations of this study (see below), and with RdRp-based trees of known nidoviruses produced in other studies [14, 21, 35]. Given that PSCNV infects planarian hosts, consistent placement of this virus in the invertebrate nidovirus clade by different analyses makes biological sense. On the other hand, the precise position of PSCNV in the invertebrate nidovirus clade remains poorly resolved for several reasons, including the highly skewed host representation in the analyzed small sample of 57 nidoviruses, and the large divergence of invertebrate nidoviruses from each other.

The dominant tree topology placed PSCNV in a very long and deeply rooted branch, which has been recognized as a suborder in the pending taxonomic proposal [130]. This is further supported by the presence of the GDD tripeptide in the RdRp C motif (S9 Fig), most common in ssRNA+ viruses other than nidoviruses, which typically (except for the arterivirus Wobbly possum disease virus, WPDV, [81]) have an SDD signature instead [131]. The pronounced divergence of PSCNV is also evident in other conserved protein domains, 3CLpro, NiRAN and ExoN, each of which carries substitutions not observed in other invertebrate or all nidoviruses.

Two prominent replacements in PSCNV 3CLpro are functionally meaningful (S7 Fig). The replacement of the otherwise invariant His by Val in the putative substrate pocket is indicative of a modified P1 substrate specificity for this enzyme, which exhibits a strong preference for Glu or Gln residues in P1 position in most other ssRNA+ viruses, including vertebrate nidoviruses [42, 88–91]. Accordingly, we were unable to identify typical 3CLpro cleavage sites at the expected inter-domain borders in the portion of the PSCNV polyprotein that must be processed by 3CLpro. Furthermore, the nucleophilic catalytic residue of PSCNV’s 3CLpro is Ser, while its counterpart in other characterized invertebrate nidoviruses is Cys. Similar variation of this residue has been described among vertebrate arteri- and toroviruses versus coronaviruses [42, 88–91], with distinct variants being associated with deeply separated virus lineages at the rank of (sub)family. Diversification of the nucleophile residue was also observed in other ssRNA+ viruses that employ 3C(L) proteases [132, 133]. This recurrent Ser-Cys toggling of the catalytic nucleophile in other well-established viral families argues against independent origins of 3CLpros in PSCNV and other nidoviruses, despite their weak sequence similarity.

Besides its exceptionally large genome size, the single-ORF organization of the PSCNV genome is unprecedented for nidoviruses. This single-ORF organization was unexpected, given that multi-ORF organization is conserved across the vast diversity of nidoviruses separated by large evolutionary distances, and infecting vertebrate or invertebrate hosts. In contrast, other large monophyletic groups of ssRNA+ viruses with comparable host ranges (e.g., the order *Picornavirales* or Flavi-like viruses), include many viruses with either single- or multi-ORF organizations, which intertwine phylogenetically [79, 132, 133].

### The PSCNV single-ORF genome may be expressed in a manner similar to that of multi-ORF nidoviruses

The use of 3CLpro as the main protease responsible for the release of key RTC subunits from polyproteins would be anticipated to remain essential in the single-ORF PSCNV. In contrast, two other conserved mechanisms of genome expression, ORF1a/1b -1 PRF and discontinuous transcription, might not be expected to operate in this virus, since they are associated with the use of multiple ORFs in nidoviruses. We reasoned otherwise, however, on the grounds that these mechanisms allow differential expression of three functionally different regions of the nidovirus genome, which are also conserved in PSCNV. We located a potential -1 PRF signal in the PSCNV genome. This signal is located at the canonical position observed in other nidoviruses, and could potentially attenuate in-frame translation downstream of the ORF1a-like region in a manner different from a mechanism used by other characterized nidoviruses, but with similar end-products (Fig 9). Such a postulated mechanism is used by encephalomyocarditis virus to attenuate the expression of replicase components in favor of capsid proteins from its main long ORF [134].

Likewise, we obtained several lines of evidence for upregulated transcription of the 3’ORFs-like region as a subgenomic RNA (Fig 10). The products of this region may also be derived from the polyprotein, but are likely required in greater abundance toward the end of the viral replication cycle, and separate expression from sg mRNA would more efficiently address this need. Importantly, no evidence, either bioinformatic or experimental, was obtained for other sg mRNAs, although we cannot exclude their existence. PSCNV’s putative TRSs are exceptionally long for nidoviruses (59 and 57 nt versus typically a dozen nt), perhaps because smaller repeats might emerge in its extraordinarily long genome by chance, interfering with transcription accuracy. Other unknown factors may also contribute to this large TRS repeat size.

The putative leader TRS (lTRS) and body TRS (bTRS), along with their predicted RNA secondary structures, suggest a model for transcriptional regulation of the PSCNV genome. We postulate that during anti-genomic RNA synthesis, the virus RTC unwinds two bTRS hairpins (Fig 10C, top). As a result, the region immediately upstream of the bTRS (yellow in the figure) becomes available for base-pairing with the 5’-terminus of the lTRS (Fig 10C, middle). This interaction will bring the two distant regions of the genome in close proximity, facilitating translocation of the nascent minus-strand from body to leader TRS (Fig 10C, bottom). The latter step is considered routine in the current model of sg RNA synthesis in well-characterized arteriviruses and coronaviruses [51, 135]. However, its mechanistic details are poorly understood and may operate differently among nidovirus families.

Although we cannot exclude the possibility that smaller ORFs are expressed by PSCNV, it seems unlikely that they would contribute substantially to the virus proteome, in line with the apparent inverse relationship between genome size and gene overlap [136]. Rather, such ORFs could be used for regulatory purposes, as in the case of the very small ORF at the border of ORF1a- and ORF1b-like regions, through the PRF mechanism proposed above.

The combined genomic and proteomic characteristics of PSCNV defy the central role of multiple ORFs in the life cycle and evolution of nidoviruses, despite their universal presence in all other nidoviruses [26, 60]. Contrary to conventional wisdom, single-ORF genome expression can involve the synthesis of subgenomic mRNAs. Rather than multi-ORF genome organization, functional constraints linked to the synteny of key replicative enzymes may be the hallmark characteristic of nidoviruses [137].

### PSCNV has acquired novel proteins with potential functions in host-virus interactions

Most of the domains that we annotated in the PSCNV giant polyprotein are homologs of canonical nidovirus domains. However, we also mapped several unique domains. Below, we discuss possible functions of five small domains, all of which plausibly modulate different aspects of virus-host interaction.

PSCNV encodes a ribonuclease T2 homolog upstream of the putative 3CLpro in the ORF1a-like region (Fig 2). Ribonucleases of the T2 family (RNase T2) are ubiquitous cellular enzymes that non-specifically cleave ssRNA in acidic environments [138]. DNA polydnaviruses and RNA pestiviruses are the only two other virus groups that are known to encode related enzymes [139, 140]. In pestiviruses, the RNase T2 homolog is a domain of secreted glycoprotein E^rns^ found in virions, but dispensable for virus entry [141]. The E^rns^ structure is supported by four disulfide bridges that are formed by eight conserved Cys residues [139]. None of these residues were found in the PSCNV RNase T2 homolog, consistent with its location in the polyprotein region that produces cytoplasmic proteins in other nidoviruses. In polydnaviruses and pestiviruses, the RNase T2 homolog modulates cell toxicity and immunity [139, 140], and a similar role could be considered for the PSCNV RNase T2 homolog. The origin of this domain in PSCNV remains uncertain due to the lack of close homologs in either its host, *S*. *mediterranea*, or other cellular and viral species.

Two other unique domains of PSCNV are fibronectin type II (FN2) homologs, protein modules of approximately 40 aa with two conserved disulfide bonds, which are ubiquitous in extracellular proteins of both vertebrates and invertebrates [142, 143]. Because of the low similarity of FN2a and FN2b to each other and other homologs, it is not clear whether they emerged by duplication or were acquired independently. No other known virus encodes an FN2 homolog (although the putative nidovirus identified in *P*. *torva* may include an ortholog of FN2b, S18 Fig), suggesting that PSCNV’s FN2 domains function in a unique aspect of its replication cycle. FN2 domains are known to possess collagen-binding activity, and are found in a variety of proteins that bind to and remodel the extracellular matrix [144, 145]. Thus, it is conceivable that these domains might play a role in the shedding or transmission of PSCNV virions. This hypothesis is compatible with the accumulation of PSCNV RNA and particles, presumably virions, in the planarian mucus-secreting cells. Besides FN2 domains, this process might also involve the Thr/Ser-rich region adjacent to FN2a in polyprotein, since Thr-rich and Thr/Ser-rich regions have been implicated in mediating adherence of fungal and bacterial extracellular (glyco) proteins to various substrates [146, 147].

The identification of the ankyrin repeats domain (ANK) in PSCNV is unprecedented and intriguing. In proteins of other origins, the ANK domain is a tandem array of ankyrin repeat motifs (~33 residues each) of variable number and divergence that fold together to form a protein-binding interface [148]. Ankyrin-containing proteins are involved in a wide range of functions in all three domains of cellular life. In viruses described to date, they have been identified exclusively in large DNA viruses with genome sizes ranging from ~100 kb to 2474 kb, the latter of *Pandoravirus salinus*, the largest viral genome described so far [38, 148–150]. Acquisition of this domain, likely from a planarian host, might have provided a PSCNV ancestor with a mechanism to evade host innate immunity. Notably, according to SmedGB [102] annotation, host proteins SMU15016868 and SMU15005918, whose C-terminal domains are the closest homologs of PSCNV ANK (Fig 6), contain a Rel homology domain (RHD) at their N-termini. This N-RHD-ANK-C domain architecture is typical of the NF-ĸB protein, a precursor of a cellular transcription factor that triggers inflammatory immune responses upon virus infection or other cell stimulation [151]. NF-ĸB is activated for translocation to the nucleus by degradation of its inhibitor, C-terminal ANK domain of NF-ĸB protein or its closely related paralog, IĸB protein [148, 152, 153]. Several large DNA viruses have been shown to encode IĸB-mimicking proteins that prevent NF-ĸB from entering the nucleus in response to the infection, and thus downregulate the host immune response [154, 155]. PSCNV ANK may represent the first example of an IĸB-mimicking protein in RNA viruses, although RNA viruses including nidoviruses can target NF-ĸB protein using other mechanisms [156]. This striking parallel between PSCNV and large DNA viruses blurs the distinction between these viruses regarding how they adapt to hosts [157]. It further highlights the exceptional coding capacity of PSCNV genome among RNA viruses.

### Emergence and evolution of the PSCNV genome: implications for the viability of large RNA genomes

The single-ORF organization of PSCNV’s exceptionally large genome is intriguing, but we cannot determine whether this association between genome size and organization is causal or coincidental from observation of a single species. In this respect, determining whether the putative nidovirus we identified in *P*. *torva* also employs a single-ORF organization could be illuminating. An evolutionary switch between multi- and single-ORF organizations, regardless of its direction, must be a multi-step process, since it affects many translation regulatory signals. In our study, we used a simple model of this process with two character states within a Bayesian phylogenetic framework, to obtain support for the single-ORF organization of PSCNV emerging from the multi-ORF organization. This approach is apparently not sensitive to the choice of domains used for phylogeny reconstruction, or inclusion of an outgroup. However, given the deep position of the PSCNV lineage in the nidovirus tree, the ambiguous rooting of PSCNV relative to other invertebrate nidovirus families, and PSCNV being the only single-ORF nidovirus known, further analysis of this transition using improved sampling of nidoviruses and their sister clades [35, 36], and more sophisticated models is warranted.

In the few experimentally characterized coronaviruses with genomes of 27–31 kb, the mutation rate is low by RNA virus standards, due to ExoN proofreading activity [34, 158, 159]. This observation is in line with the inverse relationship between genome size and mutation rate in viruses and prokaryotes [160, 161]. Accordingly, we may expect mutation rates to differ among ExoN-containing nidoviruses with different genome sizes, with PSCNV having a particularly low mutation rate. While characterization of mutation rates of PSCNV and other nidoviruses must await future studies, we already note a distinctive similarity between cellular proofreading exonucleases and ExoN of PSCNV, which separates it from its orthologs in other ExoN-positive nidoviruses. Specifically, there is a correlation between the presence of the Zn-finger motif in the exonuclease active site [33, 92] and the genome size of the biological entity encoding the exonuclease: non-PSCNV nidoviruses with genome sizes in the range of 20–34 kb include a Zn-finger embedding catalytic His, while PSCNV and DNA-based entities with genome sizes >41 kb do not (S12 Fig) [162]. Based on these observations, it is plausible that this Zn-finger might limit ExoN's capacity to improve replication fidelity while providing other benefits, and its loss in the PSCNV lineage could have been a factor promoting genome expansion.

Besides the lack of the Zn-finger in ExoN, the reported size increase of the ORF1b-like region in PSCNV relative to other nidoviruses (about 10-fold greater than expected under an assumption of uniform expansion in all genome subregions) is particularly notable in the context of the theoretical framework presented in the introduction. Briefly, expansion of RNA genomes requires escape from the so-called Eigen trap (or Eigen paradox): such genomes are confined to a low-size state, in which low replication fidelity prevents the evolution of larger genomes, which in turn prevents the evolution of greater complexity, which could introduce tools to increase replication fidelity [15]. The three-wave model of genome expansion in nidoviruses notes that the ORF1b region, which encodes the core replicative machinery, appears to play a central role in such constraints. It proposes that a wave of expansion in the ORF1b region of a common ancestor precedes and permits subsequent lineage-specific waves in the ORF1a and 3’ORFs subregions. The wave of expansion in ORF1b involved the acquisition of the ExoN proofreading exonuclease, which permitted further expansion of other subregions due to a reduced mutation rate. Until now, however, the genomes of large nidoviruses (the 20-to-34 kb size range) appeared to have reached a plateau at the low-30 kb range, associated with very little variability in the size of ORF1b among members of this group (6.9-to-8.2 kb). The three-wave model predicts that further genome expansion far beyond 34 kb would require a second cycle of waves, beginning again with ORF1b [66]. The disproportionate increase in PSCNV’s ORF1b-like region is consistent with this prediction. The acquisition of additional, still-uncharacterized domains in this region of the PSCNV genome, as well as the distinctive features of its ExoN domain, may help to explain this “second escape” from the Eigen trap. Further characterization of the PSCNV ExoN and novel ORF1b domains are required, to assess their contribution to replication fidelity and other characteristics that may be critical for faithful replication and expression of exceptionally large RNA genomes.

Our discovery of PSCNV, and analysis of its genome, show that nidoviruses can overcome the ORF1b-size barrier and adopt divergent ORF organizations. If the multi-cycle three-wave model of genome expansion in RNA viruses holds, one would expect that a large expansion of ORF1b, as evident in PSCNV, would permit yet greater expansion of the ORF1a and 3’ORFs regions in other viruses of the PSCNV lineage. Thus, nidoviruses of yet-to-be-sampled hosts might prove to have evolved even larger RNA genomes than that reported here, further decreasing the gap between virus RNA and host DNA genome sizes.

## Materials and methods

Bioinformatics Materials and Methods are described in S1 Materials and Methods in detail.

### PSCNV genome and its variants in *S*. *mediterranea* RNA-seq data

The genome sequence of human coronavirus OC43 (GenBank KY014282.1) was used to query two in-house *de novo-*assembled *Schmidtea mediterranea* transcriptomes (transcripts assembled from multiple asexual and sexual planarian stocks, designated with txv3.1 and txv3.2 prefixes, respectively) [67] using tblastx (BLAST+ v2.2.29 [163]). With E-value cut-off 10, 25 *S*. *mediterranea* transcripts were identified and used in reciprocal BLAST searches against the NCBI NR database. Two nested transcripts, txv3.2-contig_1447 (assembled from sexual planarians, GenBank BK010449) and txv3.1-contig_12746 (assembled from asexual planarians, GenBank BK010448), showed statistically significant similarity to other nidoviruses, which exceeded its similarity to other entries. Sequences of these two transcripts overlap by 23,529 nt with only 7 nt mismatches (0.03%). The larger transcript, txv3.1-contig_12746, was used to search in planarian EST clones [69, 164], which found the following overlapping clones showing >99% nucleotide identity: PL06016B2F06, PL06005B2C04. PL06007A2B12, PL06008B2B03 PL08002B1C07, and PL08001B2B04 (GenBank DN313906.1, DN309834.1, DN310382.1, DN310925.1, HO005314.1, and HO005110.1, respectively). Transcripts txv3.1-contig_12746 and txv3.2-contig_1447, and the six EST clones were assembled into an incomplete putative genome. Conflicts between overlapping sequences were always resolved in favor of the txv3.1-contig_12746 sequence. Fifteen 3’-terminal nt of the reverse complement of txv3.1-contig_12746 (“TATTATGTGATACAC”) and two 3’-terminal nt of HO005314.1 and HO005110.1 (“TG”) were discarded due to their likely technical origin. The assembled sequence contains a stop codon followed by a short untranslated region and a polyadenylated (polyA) tail. The planarian transcriptomes were surveyed again for transcripts with >50 nt overlap at the 5’-end of the incomplete genome by consecutive rounds of nucleotide BLAST. This identified txv3.1-contig_349344 (from asexual planarians; 11,647 nt; 100-nt overlap with txv3.1-contig_12746 with no mismatches; GenBank BK010447) upstream of the original transcripts, and no further extension was achieved with more BLAST iterations. The 5’-end of the genome was then extended using 5’-RACE followed by Sanger sequencing (primers in S2 Table).

Reads from planarian RNA-seq datasets (used to assemble the two transcriptomes described above, and those available from EBI ENA [165]) were mapped to the PSCNV genome sequence by either CLC Genomics Workbench 7, or Bowtie2 version 2.1.0 [166]. Read counts and coverage were estimated using SAMtools 0.1.19 [167], and genome sequence variants were called by BCFtools 1.4 [168].

### Reverse transcription, PCR, and 5’-RACE

Freshly prepared RNA from mature sexual planarians was used for cDNA synthesis (iScript, Bio-Rad) or 5’-RACE (RLM-RACE, Ambion) according to manufacturer instructions. Large overlapping amplicons across the PSCNV genome (primers in S2 Table) were amplified by standard Phusion® High-Fidelity DNA polymerase reactions, with 65°C primer annealing temperature and 10 min extension steps.

### In situ hybridization

Colorimetric and fluorescent in situ hybridizations were done following published methods [169]. Digoxigenin (DIG)-labelled PSCNV probes were generated by antisense transcription of the planarian EST clone PL06016B2F06 (GenBank DN313906.1) [69]. Following color development, all samples were cleared in 80% (v/v) glycerol and imaged on a Leica M205A microscope (colorimetric) or a Carl Zeiss LSM710 confocal microscope (fluorescent).

### Histology and transmission electron microscopy

Sexual and asexual planarians originating from the Newmark laboratory were fixed and processed for epoxy (Epon-Araldite) embedding as previously described [170]. For light-microscopic histology, 0.5 μm sections were stained with 1% (w/v) toluidine blue O in 1% (w/v) borax for 30 s at 100°C, and imaged on a Zeiss Axio Observer. For transmission electron microscopy, 50–70 nm sections were collected on copper grids, stained with lead citrate [171] and imaged with an AMT 1600 M CCD camera on a Hitachi H-7000 STEM at 75 kV. Putative virions were seen by TEM in sections from a single worm, which led us to re-examine a collection of 1697 electron micrographs, drawn from 16 additional worms (12 sexuals, four asexuals) from cultures known to harbor PSCNV. All images that included some portion of a mucus cell were chosen for further examination (n = 165); the total number of cells represented cannot be determined without three-dimensional reconstruction from serial sections, which is not practical for such large and irregularly shaped cells. No additional examples of putative viral structures were found among the specimens included in these samples.

### Genome and protein databases

For various analyses we used the following databases: PlanMine [119], Smed Unigene [102], scop70_1.75, pdb70_06Sep14 and pfamA_28.0 supplemented with profiles of conserved nidovirus domains [172–174], Uniprot [175], genome sequences representing the current 57 nidovirus species that were delineated by DEmARC [176] and recognized by ICTV on year 2016 [177], NCBI Viral Genomes Resource [178], GenBank [179] and RefSeq [180].

### Computational RNA sequence analysis

To predict RNA secondary structure and PRF sites we used Mfold web server [181] and Knot-InFrame [182], respectively. Blastn (BLAST+ v2.2.29) [163] was used to identify RNA repeats.

### Computational protein analyses

Virus protein sequences were analyzed to predict disordered regions (DisEMBL 1.5 [183]), transmembrane regions (TMHMM v.2.0), secondary structure (Jpred4 [184]), signal peptides (SignalP 4.1 [185]), N-glycosylation sites (NetNGlyc 1.0) and furin cleavage sites (ProP 1.0 [186]). Multiple sequence alignments of RNA virus proteins were generated by the Viralis platform [187]. Protein homology profile-based analyses were assisted with HMMER 3.1 [188], and HH-suite 2.0.16 [189]. To identify sites enriched with amino acid residue, distribution of each residue along polyprotein sequence was assessed using permutation test executed with a custom R script.

To establish homology for ZBD, ExoN, and N-MT, for which top HHsearch hits were under the 95% Probability threshold, we considered several criteria about the source hits: 1) being among the top three for the respective query of a database; 2) being similar to several homologous profiles in two or three databases; 3) residing in the polyprotein position conserved in nidoviruses for the respective domain (S3 Fig, S5 Table); and 4) including most residues that are critical for function of the respective domain. For ZBD, we also observed a statistically significant enrichment in cysteine (Cys) residues (S4 Fig), in line with the coordination of three Zn^2+^ ions by characterized ZBDs, which involves predominantly Cys and His residues [48, 49].

### Genome region size comparison between PSCNV and nidoviruses

Size differences between genome regions of PSCNV and nidoviruses (S1 Table) were estimated using three measures, D_1_, D_2_, and D_3_, that accounted for: 1) the region size, D_1_(region) = (p-M)/M*100%; 2) the region size variation, D_2_(region) = (p-M)/(M-m)*100%; and 3) the region size variation and genome size increase, D_3_(region) = D_2_(region)/D_2_(genome)*100%, where m and M are median and maximum sizes of the region in ExoN-containing nidoviruses, respectively, and p is the region’s size in PSCNV.

### Evolutionary analyses

Phylogeny was reconstructed by a Bayesian approach using a set of tools including BEAST 1.8.2 package [190] and ProtTest 3.4 [191] as described in [81]. BayesTraits V2 [117] was used to perform ancestral state reconstruction. Preference for a state at a node was considered statistically significant only if Log BF exceeded 2 [192].

### Visualization of results

Protein alignments were visualized with the help of ESPript 2.1 [193]. To visualize Bayesian samples of trees, DensiTree.v2.2.1 was used [194]. R was used for visualization [195].

## Supporting information

S1 Materials and Methods Detailed description of materials & methods used. (PDF)

S1 Fig PSCNV genome assembly and its verification. Contigs and 5’-RACE amplicons used to assemble the PSCNV genome sequence are shown above the PSCNV genome map (see Fig 2 for designations) by dark grey lines, with coordinates of the corresponding PSCNV genome regions specified on top of each line. The genome sequence was verified by obtaining products of expected sizes in seven RT-PCR reactions with pairs of primers that were designed to amplify large overlapping PSCNV genome regions (shown by light grey lines below the PSCNV genome map).
(TIF)

S2 Fig Characteristics of mucus cells in *S*. *mediterranea*. (*A*) Transmission electron micrograph of typical mucus cell [196]; n = nucleus. Cell bodies of such cells are filled with rough endoplasmic reticulum (RER). Distinctive mottled structures indicated by arrowheads are mucus granules. Extensions of other cells filled with these granules are also visible (mg). Inset shows a light micrograph of such a cell, stained with toluidine blue O. Mucus-rich regions of cytoplasm stain metachromatically (reddish-purple), while RER is a more-uniform blue. (*B*) Region of RER from mucus-cell cytoplasm (different cell from panel *A*) showing dilated ER lumens, and nascent mucus granules. (*C*) Higher magnification of RER in boxed region from panel *B*. (*D*) Light micrograph of cross section through ventral parenchyma (par) and epidermis (epi) stained with toluidine blue O. Reddish-purple patches indicated by arrows are fields of mucus granules (mg). (*E*) Transmission electron micrograph of ventral epithelium, showing mucus granules (mg, tinted red) just under the external surface. Scale bars: *A*, 2 μm (inset, 10 μm); *B*, 1 μm; *C*, 200 nm; *D*, 20 μm; *E*, 5 μm.
(TIF)

S3 Fig Outline of iterative HHsearch-based procedure to annotate PSCNV polyprotein. Grey bars on the top and bottom represent the PSCNV polyprotein with annotation available prior to the procedure and obtained as a result of the procedure (see S5 Table), respectively. Outline of the procedure (see S1 Materials and Methods) is presented on blue background. Iterations of the procedure are designated by Latin numbers on the left. Grey bars represent regions of PSCNV polyprotein that served as HHsearch queries to three profile databases. Basis used to split polyprotein into regions during each iteration is indicated on the right. Locations of clusters of hits with Probability (P) >95% are depicted in dark blue, with numeric indices that reflect their relative position in polyprotein, from the N- to C-terminus. Locations of accepted hits with Probability ≤95% are depicted in red, with letter indices that reflect their relative position in polyprotein, from the N- to C-terminus.
(TIF)

S4 Fig Density distribution of twenty amino acid residues and predicted functional sites of PSCNV polyprotein. Top: first derivative of cumulative amino acid residue content is plotted for each of the 20 residues with residue-specific colors; values corresponding to the N- and C- terminal 100 residues were excluded from consideration to avoid artefacts and are shown in grey. Sites enriched with a particular residue at statistically significant level are highlighted by pink background. Bottom: polyprotein location of predicted intrinsically disordered regions (D/O), N-glycosylation sites (N-glyc), signal peptidase (SPase ↓) and furin (Furin ↓) cleavage sites are shown by grey boxes, green dots, blue and red triangles, respectively (see Fig 2 for PSCNV genome map designations). Single-letter abbreviations for the amino acid residues are as follows: G, Gly; A, Ala; V, Val; L, Leu; I, Ile; M, Met; F, Phe; W, Trp; P, Pro; S, Ser; T, Thr; Y, Tyr; N, Asn; Q, Gln; C, Cys; D, Asp; E, Glu; K, Lys; R, Arg; H, His.
(TIF)

S5 Fig Alignment of PSCNV tandem repeats. Absolutely conserved residues are shown on red background and partially conserved residues in red font. Secondary structure is shown in blue. Residue numbering on top of the alignment refers to the first repeat.
(TIF)

S6 Fig MSA of RNase T2 domains of diverse origins, including PSCNV. CAS I and CAS II motifs are underlined in cyan, and catalytic histidine residues are denoted with black stars. Absolutely conserved residues are shown on red background and partially conserved residues in red font. Secondary structure is shown in blue. Residue numbering above of the alignment refers to the top sequence.
(TIF)

S7 Fig MSA of 3CLpro domains from four distantly related ExoN-positive nidoviruses and PSCNV (4438–4664 aa). The aligned proteases employ catalytic residues that include as the principal nucleophile either a Cys residue (TGEV, NDiV, GAV) or a Ser residue (BRV, PSCNV). Columns containing TGEV 3CLpro catalytic dyad residues are marked by black stars. TGEV 3CLpro Val84 residue that is spatially equivalent to the catalytic acidic residue of serine proteases is marked with empty circle. Residues of the TGEV 3CLpro substrate-binding pocket are underlined with green bars [87]. Absolutely conserved residues are shown on red background and partially conserved residues in red font. Secondary structure is shown in blue. Residue numbering on top of the alignment refers to TGEV nsp5.
(TIF)

S8 Fig MSA of NiRAN domains from five distantly related nidoviruses and PSCNV (6181–6410 aa). Conserved motifs are underlined in green. Absolutely conserved residues are shown on red background and partially conserved residues in red font. Secondary structure is shown in blue. Residue numbering on top of the alignment refers to EAV nsp9.
(TIF)

S9 Fig MSA of RdRp domains from five distantly related nidoviruses and PSCNV (6632–7125 aa). Conserved motifs are underlined in green. Absolutely conserved residues are shown on red background and partially conserved residues in red font. Secondary structure is shown in blue. Residue numbering on top of the alignment refers to EAV nsp9.
(TIF)

S10 Fig MSA of ZBD domains from four distantly related ExoN-positive nidoviruses and PSCNV (7379–7484 aa). Residues of three zinc fingers coordinating zinc ions (delineated according to the solved EAV ZBD structure [48]) are marked by red, blue and green triangles, respectively. Absolutely conserved residues are shown on red background and partially conserved residues in red font. Secondary structure is shown in blue. Residue numbering on top of the alignment refers to SARS-CoV nsp13.
(TIF)

S11 Fig MSA of HEL1 domains from four distantly related ExoN-positive nidoviruses and PSCNV (7718–8056 aa). Conserved motifs are highlighted by color indicating their predominant function [47]: NTP binding and hydrolysis, green; nucleic acid binding, blue; coupling of NTP and nucleic acid binding, purple. Absolutely conserved residues are shown on red background and partially conserved residues in red font. Secondary structure is shown in blue. Residue numbering on top of the alignment refers to SARS-CoV nsp13.
(TIF)

S12 Fig MSA of ExoN domains from four distantly related nidoviruses and PSCNV (8342–8629 aa). Columns containing SARS-CoV ExoN catalytic residues and Asp243 residue, essential for nuclease activity, are marked by black stars and circle, respectively. Green and orange triangles mark columns that contain residues of two SARS-CoV ExoN zinc fingers; empty circles indicate columns that contain SARS-CoV ExoN residues interacting with nsp10 (the majority of such residues are not depicted, as they belong to the N-terminal 1–76 aa region of SARS-CoV nsp14) [92]. Absolutely conserved residues are shown on red background and partially conserved residues in red font. Secondary structure is shown in blue. Residue numbering on top of the alignment refers to SARS-CoV nsp14.
(TIF)

S13 Fig MSA of N-MT domains from three distantly related ExoN-positive nidoviruses and PSCNV (8630–8874 aa). Columns containing SARS-CoV SAH- and GpppA-binding residues, such that their mutation significantly reduced N7-MTase activity, are marked by black and empty circles, respectively. Residues of SARS-CoV N-MT involved in formation of zinc-finger are marked by green triangles [92]. Absolutely conserved residues are shown on red background and partially conserved residues in red font. Secondary structure is shown in blue. Residue numbering on top of the alignment refers to SARS-CoV nsp14.
(TIF)

S14 Fig MSA of O-MT domains from four distantly related ExoN-positive nidoviruses and PSCNV (9110–9406 aa). Columns containing SARS-CoV O-MT catalytic tetrad residues are marked by black stars. SARS-CoV O-MT residues involved in interaction with nsp10 are marked by empty circles. Loops constituting SAM-binding cleft and cap-binding groove of SARS-CoV O-MT are underlined in orange and green, respectively [98]. Absolutely conserved residues are shown on red background and partially conserved residues in red font. Secondary structure is shown in blue. Residue numbering on top of the alignment refers to SARS-CoV nsp16.
(TIF)

S15 Fig Comparison of FN2 domains from human matrix metalloproteinase-2 and PSCNV. Shown is the MSA of the third FN2 domain of human matrix metalloproteinase-2 (MMP2) and FN2a (10555–10613 aa) and FN2b (12186–12233 aa) of PSCNV. Pairs of cysteine residues, predicted to form disulfide bridges, are designated by blue bars (first pair) and stars (second pair). Absolutely conserved residues are shown on red background and partially conserved residues in red font. Secondary structures, derived from MMP2 1J7M and predicted for PSCNV domains, is shown in blue. Residue numbering above the alignment refers to the top sequence.
(TIF)

S16 Fig Comparison of PSCNV ANK domain with most closely related flatworm proteins. A representative set of three proteins of flatworms most closely related to the PSCNV ANK domain (Fig 6) was included in the presented MSA. Individual ankyrin repeats in PSCNV polyprotein are underlined by black dashed lines. Signature motifs of individual ankyrin repeats are highlighted in green and orange. Absolutely conserved residues are shown on red background and partially conserved residues in red font. Predicted secondary structure is shown in blue. Residue numbering above the alignment refers to the top sequence.
(TIF)

S17 Fig Phylogeny reconstructed by BEAST based on the alignment of RdRp core of PSCNV, nidoviruses and astroviruses. Bayesian sample of trees is shown in green, consensus tree with the highest clade support is shown in blue. Support for multiple ORFs vs single ORF in the genome of MRCA of nidoviruses as calculated using BayesTraits V2 is indicated. Short arrows show three most frequently observed (percentages of trees in the sample indicated) positions of the PSCNV branch, which collectively account for 88.7% of PSCNV topologies in the tree sample analyzed. Position of the PSCNV branch in the depicted consensus tree is the one that is most frequently observed (54.7% of trees in the sample).
(TIF)

S18 Fig Statistically significant BLAST hits between translated contigs of PlanMine database and PSCNV polyprotein. Contigs from two assemblies, dd_Ptor_v3 and uc_Smed_v2, are shown as white rectangles. For each hit, depicted as a grey band, a frame in which the contig was translated (“F” stands for forward), E-value, and percentage of amino acid identity are specified. Contig ox_Smed_v2_19364 was also identified but is not depicted due to being identical (with the exception of four 3’-terminal nt) to uc_Smed_v2_Contig50508. See Fig 2 for PSCNV genome map designations.
(TIF)

S1 Table Genome sequences and size characteristics of representatives of nidovirus species used in bioinformatics analyses. (PDF)

S2 Table Primers used for viral genome detection, 5’-RACE, and genome-wide overlapping amplification. (PDF)

S3 Table *S*. *mediterranea* RNA-seq datasets screened for presence of PSCNV reads. (PDF)

S4 Table PSCNV genome sequence variants in the 28389–41000 nt region. (PDF)

S5 Table Domain identification in PSCNV polyprotein through comparison with various protein databases using HHsearch. (PDF)

S6 Table Genome region size increases in PSCNV compared to ExoN-containing nidoviruses. (PDF)
