# Supplementary material for: A planarian nidovirus expands the limits of RNA genome size
Source: PLoS Pathog. 2018 Nov 1;14(11):e1007314. doi: 10.1371/journal.ppat.1007314 (PMC6211748; doi:10.1371/journal.ppat.1007314)
Supplement: S1 Materials and Methods — (PDF) [file ppat.1007314.s001.pdf]

S1 Materials and Methods

Search for nido-like viruses in transcriptomes of *S. mediterranea*. Two *de novo* transcriptomes of planarian *S. mediterranea* [1] were searched for sequences similar to human coronavirus OC43 (GenBank KY014282.1) by the tblastx application in BLAST+ v2.2.29 [2] using BLOSUM80 matrix, word size 2, and E-value cut-off 10. The resulting hits were translated in six frames by EMBOSS:6.6.0.0 transeq [3] and used to search for similar domains in the NCBI non-redundant protein database (NR) by deltablast (BLAST+ v2.2.29) [4] with the same parameters, except using an E-value cut-off of 1.

Assessment of PSCNV genome coverage by RNA-seq reads. Reads from five independent in-house *S. mediterranea* RNA-seq datasets, previously used to assemble the transcriptomes in which PSCNV was found [1], were mapped to the PSCNV genome sequence (1–41103 nt) using either CLC Genomics Workbench 7 (alignment criteria: mismatch cost 2, insertion/deletion cost 3, length fraction > 0.9, similarity fraction > 0.9), or Bowtie2 version 2.1.0 with default parameters [5]. PSCNV genome coverage by reads from each dataset was estimated using SAMtools 0.1.19 [6].

Search for viruses related to PSCNV in planarian RNA database. The PlanMine database [7] was downloaded from http://planmine.mpi-cbg.de/planmine/ on 2017.10.06, contigs were translated in six frames by EMBOSS:6.6.0.0 transeq [3], and compared with PSCNV polyprotein by blastp (BLAST+ v2.2.29) [2]. Only hits with E-value < 0.001 were considered with the exception of those that involved PSCNV HEL1 or ANK domains. For these domains, whose homologs are common in many proteomes, an additional condition for consideration was to have one or more extra hits between the particular contig translation and other regions of PSCNV polyprotein.

Identification of PSCNV variants in *S. mediterranea* RNA-seq data. RNA-seq data from fourteen *S. mediterranea* BioProjects (Table S3) were downloaded from the EBI ENA [8] and aligned to PSCNV genome sequence (1–41103 nt) using Bowtie2 version 2.1.0 with default parameters [5]. Read counts and coverage were estimated using SAMtools 0.1.19 [6]. Genome sequence variants were called by BCFtools 1.4 [9] with the following parameters: maximum per-file depth 100000 (including for INDEL calling), the original variants calling method, *p*-value threshold 0.5, ploidy 1.

Nidoviral species and their genomes and proteomes. One representative genome sequence per nidovirus species [10] (in total 57 sequences) was selected for this study (Table S1). Their proteomes, including polyprotein sizes (Fig. 5) and protein domain locations (Fig. 2), were defined using respective entries in the RefSeq database [11] (where available), the literature, and comparative sequence analysis. Boundaries of genome regions were defined as follows: ORF1a region, from the first nucleotide (nt) of the ORF1a start codon to the last nt of the last in-frame codon translated before ORF1a/1b programmed ribosomal frameshifting (PRF); ORF1b region, from the first nt of the first ORF1b codon translated after ORF1a/1b PRF to the last nt of the ORF1b stop codon; 3’ORFs region, from the first nt following ORF1b stop codon to the last nt of the stop codon of the most 3’-terminal ORF.

The single-ORF genome organization of PSCNV presents a distinctive challenge for defining boundaries of three genome regions evident in the multi-ORF nidoviruses. We defined two boundaries, tentatively equivalent to the ORF1a/ORF1b and ORF1b/3’ORFs, in vicinity of the protein motifs universally conserved in all nidoviruses and PSCNV. As result, three regions were defined as follows: ORF1a-like, from the first nt of the start codon of the main ORF to the 18512 nt, the predicted -1PRF site 240 nt upstream of the codon encoding absolutely conserved lysine residue of the NiRAN An motif; ORF1b-like, from the 18513 nt to the 28346 nt, which is 260 nt downstream of the codon encoding catalytic glutamate residue of O-MT; 3’ORFs-like, from the 28347 nt to the last nt of the main ORF stop codon.

RNA virus proteins. For the purpose of this study (Fig. 5), we compiled a list of RNA virus proteins larger than 1000 amino acids and expressed without PRF, based on the information available from the NCBI Viral Genomes Resource on 2017.04.13 [12] and RefSeq entries [11] specified there.

Virus discovery and genome sequencing timelines. The number of viral genomes that were sequenced each year, starting from 1982, was estimated using NCBI Entrez query [13], as the number of GenBank Nucleotide database (2018.01.02) entries belonging to the "Viral sequences" division and containing the phrase "complete cds" in the title, with publication dates within the year of interest [14]. To plot timelines of discovery of viruses with largest RNA and DNA genomes, those viruses were identified and associated information was retrieved for each year using NCBI Viral Genomes Resource on 2017.04.13 [12] and the relevant literature. We used poliovirus (PV), and nidoviruses avian bronchitis virus (IBV), mouse hepatitis virus (MHV), beluga whale coronavirus SW1 (BWCoV), and ball python nidovirus (BPNV) to highlight the longest RNA virus genome at 1981 and from 1987 onward, respectively, in Fig. 1A (see Table S1 for the genome sizes of the above nidoviruses).

Multiple sequence alignments of proteins. Multiple sequence alignments (MSAs) of 3CLpro, NiRAN, RdRp, ZBD, HEL1, ExoN, N-MT and O-MT protein domains were prepared for individual nidovirus families using the Viralis platform [15] and assisted by the HMMER 3.1 [16], Muscle 3.8.31 [17] and ClustalW 2.012 [18] programs in default modes. For each domain, MSAs of different nidovirus families and PSCNV were later combined using ClustalW in the profile mode, with subsequent manual local refinement. MSAs of RNase T2, FN2, and ANK domains and PSCNV tandem repeats were prepared using MAFFT v7.123b [19].

Host proteome. Proteome of *S. mediterranea*, Smed Unigene 2015.02.17 [20], was obtained from http://smedgd.stowers.org/.

Identification of ORFs. PSCNV genome was scanned for ORFs in six reading frames by ORFfinder (https://www.ncbi.nlm.nih.gov/orffinder/) using the standard genetic code and minimal ORF length of 150 nt.

Protein secondary structure retrieval and prediction. Secondary structure was retrieved from PDB structures using the DSSP database [21] via the MRS system [22] for the following proteins: TGEV 3CLpro, 1LVO [23]; SARS-CoV ExoN and N-MT, 5C8T [24]; SARS-CoV O-MT, 3R24 [25]; POLG_BVDVC, 4DW3 [26]; RNT2_HUMAN, 3T0O [27]; MMP2_HUMAN, 1J7M [28]. In all other cases, secondary structure was predicted for individual sequences using Jpred4 [29] in the MSA mode.

Identification of PSCNV polyprotein sequence regions enriched in particular amino acid residues. To identify polyprotein regions enriched in a given amino acid residue, we calculated the distribution of that residue along the polyprotein and compared it to that of permuted sequences within a statistical framework that was applied to each residue type separately. Specifically, we calculated the cumulative count of a particular residue type within the ever expanding [1, *i*] window, where 1 is the first position and *i* is each position from the 1st to the last 13,556th in the polyprotein. The produced discrete data were approximated by R function "smooth.spline" with default parameters, and the first derivative of the approximation was obtained for each *i* value [30]. The procedure was then applied to 100 random permutations of the polyprotein sequence, and mean μ and standard deviation (SD) σ of the resulting derivative values were used to define significance threshold $T=\mu+Z(1-0.05/L)*\sigma=\mu+4.5*\sigma$, where Z( ) is a quantile function of the standard normal distribution and L is the polyprotein sequence length. Protein sequence regions with derivative values larger than the threshold (4.5 SD above the mean) were considered enriched in the amino acid residue. To avoid artefacts of the approximation, we excluded data corresponding to the N- and C- terminal 100 amino acids of the polyprotein.

Prediction of disordered protein regions. Intrinsically disordered regions of the PSCNV polyprotein were predicted by DisEMBL 1.5 using Remark465 predictor with default parameters [31].

Prediction of transmembrane regions. Transmembrane (TM) regions of proteins were predicted using TMHMM Server v.2.0 (http://www.cbs.dtu.dk/services/TMHMM/) with default parameters. To conform to the input sequence length limitation (8000 aa), PSCNV polyprotein sequence was split into consecutive 8000 and 6556 aa fragments, with a 1000 aa overlap; predictions belonging to the overlap region were accepted even if supported only for one of the fragments.

Prediction of signal peptides. To predict signal peptides, SignalP 4.1 [32] was used. Prediction was made for all PSCNV polyprotein sequence fragments of length 70 aa with default parameters. A D-score threshold of 0.75 was applied to predictions; when predicted signal peptides overlapped, the one with the highest D-score was selected.

Prediction of N-glycosylation sites. N-glycosylation sites were predicted using NetNGlyc 1.0 Server (http://www.cbs.dtu.dk/services/NetNGlyc/) with default parameters. Only predictions with potential above 0.75, supported by all nine networks were accepted. Predictions where potentially glycosylated asparagine is followed by proline, and predictions overlapping with TM helices were discarded. To conform to the input sequence length limitation (4000 aa), PSCNV polyprotein sequence was split into 4000 aa fragments, with 1000 aa overlaps starting from the N-terminus (the most C-terminal fragment was 1556 aa long; 5 fragments in total); predictions belonging to the overlaps were accepted even if supported only for one of the fragments.

Prediction of furin cleavage sites. Furin cleavage sites were predicted by ProP 1.0 Server [33] in default mode and with the PSCNV polyprotein sequence submitted as overlapping fragments as described for the N-glycosylation sites prediction.

Identification of protein sequence repeats. To search for repeats in PSCNV polyprotein, its sequence was compared to itself using an in-house version of HHalign 2.0.16 with the following parameters: SMIN score threshold 5, E-value threshold 10, local alignment mode, realignment by the MAC algorithm not applied, up to 1000 alternative alignments allowed to be shown [34].

Identification of protein domains conserved in PSCNV and other viruses or hosts. We used HHsearch 2.0.16 [35] to query databases scop70_1.75, pdb70_06Sep14 and modified pfamA_28.0 [36-38] with the PSCNV polyprotein fragments using iterative procedure. The modified pfamA_28.0 included original pfamA_28.0 and Hidden Markov Model (HMM) profiles of the most conserved nidovirus domains 3CLpro, NiRAN, RdRp, ZBD, HEL1, ExoN, N-MT, and O-MT, composed of sequences representing *Coronaviridae*, *Mesoniviridae* and *Roniviridae* species (Table S1). This modification facilitates statistical evaluation of similarity between the PSCNV polyprotein and the nidovirus conserved domains within a framework that is used for the pfamA domains. During the first iteration of the procedure, polyprotein was split into fragments by TM clusters (TM helices separated by less than 300 aa), tandem repeats and Thr-rich region. Overlapping hits characterized by Probability above 95% were clustered, clusters were used to split polyprotein into smaller regions that served as HHsearch queries on subsequent iteration. Procedure was repeated until iteration during which no hits satisfying the 95% Probability threshold were detected. Finally, regions of polyprotein without hits were split into successive fragments of 300 aa length starting from N- and C-termini (shorter regions were discarded), which were again scanned for hits by HHsearch. To evaluate the statistical significance of HHsearch hits, we used two measures, E-value and Probability (estimates probability of the query being homologous to the target). We considered homology to be established for PSCNV regions and a database entry that were connected by hits with Probability >95%, and made additional considerations when evaluating hits with Probability ≤95%, as advised in the HH-suite User Guide [35]. In this subsequent analysis, we considered rank, size, and E-value of hits, and conservation of key functionally important residues in the query.

Search for the closest homologs of PSCNV protein domains not previously described in nidoviruses. PSCNV protein domains that were *not* previously described in nidoviruses (RNase T2, FN2, ANK) were compared with Uniprot (2017.01.16) [39] and Smed Unigene (2015.02.17) [20] databases using blastp (BLAST+ v2.2.29) [2]. Domains were extended by 100 amino acids at N- and

C-termini in order to capture homology extending beyond that identified by HHsearch. The FN2a domain was not extended at the N-terminus because of the low-complexity Thr-rich domain located immediately upstream. For searches in Smed Unigene database, effective length of the search space was made equal to that of the search in Uniprot with the same query, in order to make E-values comparable. Domain composition of Smed Unigene hits was obtained from this database, while that of Uniprot hits – from InterPro database [40].

Identification of individual ankyrin repeats. Full alignments corresponding to Ank and Ank_3 families of Pfam 28.0 [38], each representing individual ankyrin repeat, were combined. The resulting alignment was converted to HMM profile by HHmake 2.0.16. The HMM profile had a consensus "xxxGxTpLHxAxxxxxxxxivxxLlxxGadxnxxd", with positions 6–9 and 20–25 corresponding to two conserved ankyrin repeat motifs: TPLH and V/I-V-x-L/V-L-L [41]. It was compared to the PSCNV Ankyrin domain (11360–11570 aa) using in-house version of HHalign 2.0.16 (parameters as detailed for comparison of PSCNV polyprotein sequence with itself). Hits to the PSCNV polyprotein were regarded as individual ankyrin repeats if the alignment included 6–25 positions of the HMM profile.

Phylogeny reconstruction. Phylogeny was reconstructed based on the MSA of the conserved core of RdRp domain (517 columns, 1958–2356 aa in the EAV pp1ab CAC42775.2 of X53459.3), including one representative of each nidovirus species (Table S1) and PSCNV, as well as an outgroup consisting of viruses of two species prototyping the astrovirus genera (*Avastrovirus 1*, Y15936.2; *Mamastrovirus 1*, L23513.1) [42]. Phylogeny was reconstructed using BEAST 1.8.2 package [43] with the model of amino acid replacement selected by ProtTest 3.4 [44] (Akaike information criterion and Bayesian information criterion employed for model selection; maximum likelihood (ML) tree topology optimization strategy utilizing subtree pruning and regrafting moves). Both strict clock and relaxed clock with uncorrelated log-normal rate distribution were tested, and a better-fitting model was selected based on Bayes factor estimate. Markov Chain Monte Carlo (MCMC) chains were run for 10 million iterations and sampled every 1000 iterations; the first 10% iterations were discarded as burn-in. Mixing and convergence were verified with the help of Tracer 1.5 (http://beast.bio.ed.ac.uk/Tracer). Results were summarized as maximum clade credibility (MCC) tree. R package APE 3.5 was used to calculate percentage of trees in the Bayesian sample, characterized by various phylogenetic positions of PSCNV [45]. The same procedure was used to reconstruct 1.) a phylogeny based on the MSA of five nidovirus-wide conserved domains (3CLpro, NiRAN, RdRp, ZBD, HEL1; 1569 columns, 1065–1227, 1740–1881, 1958–2356, 2373–2427, 2520–2774 aa in the EAV pp1ab CAC42775.2 of X53459.3) including one representative of each nidovirus species (Table S1) and PSCNV; 2.) a phylogeny based on the MSA of PSCNV ANK and its closest cellular homologs (Fig. S16, from first to last column without gaps).

Ancestral state reconstruction. BayesTraits V2, MCMC method was used to test support for one ancestral state over the other at a given node [46]. A sample of phylogenetic trees, reconstructed by BEAST as detailed above, was utilized. State "1", single ORF, was assigned to PSCNV, while state "0", multiple ORFs, was assigned to all other viruses in the phylogeny. We also run a version of the analysis where state "-", that is the lack of information about genome organization, was assigned to astroviruses. To derive prior distributions for the rate parameters of the model, we calculated a ML estimate of the rate parameters on each tree in our sample, and set mean and variance of the gamma priors to conform to those of the obtained distributions. MCMC chains (10 million iterations, first 1% iterations discarded as burn-in) were run with the node of interest fossilized in both states. The Harmonic Mean value was recorded at the final iteration of each chain. Log Bayes Factor (BF) was calculated as twice the difference between Harmonic Mean values of the better and the worse fitting models. The procedure was repeated three times and, if the same model was favored every time, only the smallest value of the Log BF was reported. Preference for a state at a node was considered statistically significant only if Log BF exceeded 2 [47].

Identification of putative transcription-regulating sequences (TRSs). Nidoviruses utilize non-adjacent nucleotide repeats (conserved signals) in the 5'-UTR and the second half of the genome to regulate synthesis of subgenomic (sg) mRNAs (transcription). These repeats are known as leader and

body transcription-regulating sequences, lTRS and bTRS, respectively. To search for potential TRSs, the 5'-UTR sequence was compared with the PSCNV genome using blastn (BLAST+ v2.2.29) [2].

RNA secondary structure prediction. RNA secondary structure prediction for PSCNV genome regions encompassing lTRS and bTRS (1–9000 nt and 20441–29440 nt, respectively) was assisted by the Mfold web server [48]. Only the top-ranking predictions with the lowest free energy were considered. Maximal distance between paired bases was set to 150 nt. Free energy for fragments of the prediction was calculated using http://unafold.rna.albany.edu/?q=mfold/Structure-display-and-free-energy-determination.

PRF site prediction. Analysis was conducted for PSCNV and SARS-CoV. KnotInFrame [49] was applied to a 1000 nt genome region immediately upstream of the region encoding the NiRAN An motif. Only the top prediction for each virus was considered.

Visualization of the results. Protein alignments were visualized by ESPript 2.1 [50] using the Risler similarity matrix [51] and similarity global score 0.7. To visualize Bayesian samples of trees, DensiTree.v2.2.1 was used [52]. R was used extensively for visualization [30].

1. Saberi A, Jamal A, Beets I, Schoofs L, Newmark PA. GPCRs Direct Germline Development and Somatic Gonad Function in Planarians. PLoS Biol. 2016;14(5):e1002457. doi: 10.1371/journal.pbio.1002457. PubMed PMID: 27163480; PubMed Central PMCID: PMCPMC4862687.
2. Altschul SF, Gish W, Miller W, Myers EW, Lipman DJ. Basic local alignment search tool. J Mol Biol. 1990;215(3):403-10. doi: 10.1016/S0022-2836(05)80360-2. PubMed PMID: 2231712.
3. Rice P, Longden I, Bleasby A. EMBOSS: the European Molecular Biology Open Software Suite. Trends Genet. 2000;16(6):276-7. PubMed PMID: 10827456.
4. Boratyn GM, Schaffer AA, Agarwala R, Altschul SF, Lipman DJ, Madden TL. Domain enhanced lookup time accelerated BLAST. Biol Direct. 2012;7:12. doi: 10.1186/1745-6150-7-12. PubMed PMID: 22510480; PubMed Central PMCID: PMCPMC3438057.
5. Langmead B, Salzberg SL. Fast gapped-read alignment with Bowtie 2. Nat Methods. 2012;9(4):357-9. doi: 10.1038/nmeth.1923. PubMed PMID: 22388286; PubMed Central PMCID: PMCPMC3322381.
6. Li H, Handsaker B, Wysoker A, Fennell T, Ruan J, Homer N, et al. The Sequence Alignment/Map format and SAMtools. Bioinformatics. 2009;25(16):2078-9. doi: 10.1093/bioinformatics/btp352. PubMed PMID: 19505943; PubMed Central PMCID: PMCPMC2723002.
7. Brandl H, Moon H, Vila-Farre M, Liu SY, Henry I, Rink JC. PlanMine--a mineable resource of planarian biology and biodiversity. Nucleic Acids Res. 2016;44(D1):D764-73. doi: 10.1093/nar/gkv1148. PubMed PMID: 26578570; PubMed Central PMCID: PMCPMC4702831.
8. Silvester N, Alako B, Amid C, Cerdeno-Tarraga A, Clarke L, Cleland I, et al. The European Nucleotide Archive in 2017. Nucleic Acids Res. 2017. doi: 10.1093/nar/gkx1125. PubMed PMID: 29140475.
9. Li H. A statistical framework for SNP calling, mutation discovery, association mapping and population genetical parameter estimation from sequencing data. Bioinformatics. 2011;27(21):2987-93. doi: 10.1093/bioinformatics/btr509. PubMed PMID: 21903627; PubMed Central PMCID: PMCPMC3198575.
10. Adams MJ, Lefkowitz EJ, King AM, Harrach B, Harrison RL, Knowles NJ, et al. Ratification vote on taxonomic proposals to the International Committee on Taxonomy of Viruses (2016). Arch Virol. 2016;161:2921-49. doi: 10.1007/s00705-016-2977-6. PubMed PMID: 27424026.
11. O'Leary NA, Wright MW, Brister JR, Ciufo S, Haddad D, McVeigh R, et al. Reference sequence (RefSeq) database at NCBI: current status, taxonomic expansion, and functional annotation. Nucleic Acids Res. 2016;44(D1):D733-45. doi: 10.1093/nar/gkv1189. PubMed PMID: 26553804; PubMed Central PMCID: PMCPMC4702849.
12. Brister JR, Ako-Adjei D, Bao Y, Blinkova O. NCBI viral genomes resource. Nucleic Acids Res. 2015;43(Database issue):D571-7. doi: 10.1093/nar/gku1207. PubMed PMID: 25428358; PubMed Central PMCID: PMCPMC4383986.
13. Gibney G, Baxevanis AD. Searching NCBI databases using Entrez. Curr Protoc Bioinformatics. 2011;Chapter 1:Unit 1 3. doi: 10.1002/0471250953.bi0103s34. PubMed PMID: 21633942.
14. Benson DA, Cavanaugh M, Clark K, Karsch-Mizrachi I, Ostell J, Pruitt KD, et al. GenBank. Nucleic Acids Res. 2017;45(D1):D37–D42. doi: 10.1093/nar/gkx1094. PubMed PMID: 29140468.
15. Gorbalenya AE, Lieutaud P, Harris MR, Coutard B, Canard B, Kleywegt GJ, et al. Practical application of bioinformatics by the multidisciplinary VIZIER consortium. Antiviral Res. 2010;87(2):95-110. doi: S0166-3542(10)00034-3 [pii];10.1016/j.antiviral.2010.02.005 [doi].
16. Eddy SR. A new generation of homology search tools based on probabilistic inference. Genome Inform. 2009;23(1):205-11. doi: 9781848165632_0019 [pii].
17. Edgar RC. MUSCLE: multiple sequence alignment with high accuracy and high throughput. Nucleic Acids Res. 2004;32(5):1792-7. doi: 10.1093/nar/gkh340 [doi];32/5/1792 [pii].

18. Larkin MA, Blackshields G, Brown NP, Chenna R, McGettigan PA, McWilliam H, et al. Clustal W and Clustal X version 2.0. Bioinformatics. 2007;23(21):2947-8. doi: btm404 [pii];10.1093/bioinformatics/btm404 [doi].
19. Katoh K, Standley DM. MAFFT multiple sequence alignment software version 7: improvements in performance and usability. Mol Biol Evol. 2013;30(4):772-80. doi: mst010 [pii];10.1093/molbev/mst010 [doi].
20. Robb SM, Gotting K, Ross E, Sanchez AA. SmedGD 2.0: The Schmidtea mediterranea genome database. Genesis. 2015;53(8):535-46. doi: 10.1002/dvg.22872 [doi].
21. Touw WG, Baakman C, Black J, te Beek TA, Krieger E, Joosten RP, et al. A series of PDB-related databanks for everyday needs. Nucleic Acids Res. 2015;43(Database issue):D364-8. doi: 10.1093/nar/gku1028. PubMed PMID: 25352545; PubMed Central PMCID: PMCPMC4383885.
22. Hekkelman ML, Vriend G. MRS: a fast and compact retrieval system for biological data. Nucleic Acids Res. 2005;33(Web Server issue):W766-9. doi: 10.1093/nar/gki422. PubMed PMID: 15980580; PubMed Central PMCID: PMCPMC1160183.
23. Anand K, Palm GJ, Mesters JR, Siddell SG, Ziebuhr J, Hilgenfeld R. Structure of coronavirus main proteinase reveals combination of a chymotrypsin fold with an extra alpha-helical domain. EMBO J. 2002;21(13):3213-24. doi: 10.1093/emboj/cdf327. PubMed PMID: 12093723; PubMed Central PMCID: PMCPMC126080.
24. Ma Y, Wu L, Shaw N, Gao Y, Wang J, Sun Y, et al. Structural basis and functional analysis of the SARS coronavirus nsp14-nsp10 complex. Proc Natl Acad Sci U S A. 2015;112(30):9436-41. doi: 1508686112 [pii];10.1073/pnas.1508686112 [doi].
25. Chen Y, Su C, Ke M, Jin X, Xu L, Zhang Z, et al. Biochemical and structural insights into the mechanisms of SARS coronavirus RNA ribose 2'-O-methylation by nsp16/nsp10 protein complex. PLoS Pathog. 2011;7(10):e1002294. doi: 10.1371/journal.ppat.1002294 [doi];PPATHOGENS-D-11-00791 [pii].
26. Krey T, Bontems F, Vonrhein C, Vaney MC, Bricogne G, Rumenapf T, et al. Crystal structure of the pestivirus envelope glycoprotein E(rns) and mechanistic analysis of its ribonuclease activity. Structure. 2012;20(5):862-73. doi: S0969-2126(12)00136-0 [pii];10.1016/j.str.2012.03.018 [doi].
27. Thorn A, Steinfeld R, Ziegenbein M, Grapp M, Hsiao HH, Urlaub H, et al. Structure and activity of the only human RNase T2. Nucleic Acids Res. 2012;40(17):8733-42. doi: 10.1093/nar/gks614. PubMed PMID: 22735700; PubMed Central PMCID: PMCPMC3458558.
28. Briknarova K, Gehrmann M, Banyai L, Tordai H, Patthy L, Llinas M. Gelatin-binding region of human matrix metalloproteinase-2: solution structure, dynamics, and function of the COL-23 two-domain construct. J Biol Chem. 2001;276(29):27613-21. doi: 10.1074/jbc.M101105200. PubMed PMID: 11320090.
29. Drozdetskiy A, Cole C, Procter J, Barton GJ. JPred4: a protein secondary structure prediction server. Nucleic Acids Res. 2015;43(W1):W389-94. doi: 10.1093/nar/gkv332. PubMed PMID: 25883141; PubMed Central PMCID: PMCPMC4489285.
30. R Core Team. R: A Language and Environment for Statistical Computing. Vienna, Austria: R Foundation for Statistical Computing; 2013.
31. Linding R, Jensen LJ, Diella F, Bork P, Gibson TJ, Russell RB. Protein disorder prediction: implications for structural proteomics. Structure. 2003;11(11):1453-9. PubMed PMID: 14604535.
32. Petersen TN, Brunak S, von HG, Nielsen H. SignalP 4.0: discriminating signal peptides from transmembrane regions. Nat Methods. 2011;8(10):785-6. doi: nmeth.1701 [pii];10.1038/nmeth.1701 [doi].
33. Duckert P, Brunak S, Blom N. Prediction of proprotein convertase cleavage sites. Protein Eng Des Sel. 2004;17(1):107-12. doi: 10.1093/protein/gzh013. PubMed PMID: 14985543.
34. Gulyaeva A, Dunowska M, Hoogendoorn E, Giles J, Samborskiy D, Gorbalenya AE. Domain organization and evolution of the highly divergent 5' coding region of genomes of arteriviruses including the novel possum nidovirus. J Virol. 2017;91(6). doi: 10.1128/JVI.02096-16. PubMed PMID: 28053107.
35. Soding J. Protein homology detection by HMM-HMM comparison. Bioinformatics. 2005;21(7):951-60. doi: bti125 [pii];10.1093/bioinformatics/bti125 [doi].

36. Andreeva A, Howorth D, Chandonia JM, Brenner SE, Hubbard TJ, Chothia C, et al. Data growth and its impact on the SCOP database: new developments. Nucleic Acids Res. 2008;36(Database issue):D419-25. doi: 10.1093/nar/gkm993. PubMed PMID: 18000004; PubMed Central PMCID: PMCPMC2238974.
37. Berman HM, Westbrook J, Feng Z, Gilliland G, Bhat TN, Weissig H, et al. The Protein Data Bank. Nucleic Acids Res. 2000;28(1):235-42. PubMed PMID: 10592235; PubMed Central PMCID: PMCPMC102472.
38. Finn RD, Bateman A, Clements J, Coggill P, Eberhardt RY, Eddy SR, et al. Pfam: the protein families database. Nucleic Acids Res. 2014;42(Database issue):D222-D30. doi: gkt1223 [pii];10.1093/nar/gkt1223 [doi].
39. UniProt Consortium. UniProt: a hub for protein information. Nucleic Acids Res. 2015;43(Database issue):D204-D12. doi: gku989 [pii];10.1093/nar/gku989 [doi].
40. Finn RD, Attwood TK, Babbitt PC, Bateman A, Bork P, Bridge AJ, et al. InterPro in 2017-beyond protein family and domain annotations. Nucleic Acids Res. 2017;45(D1):D190-D9. doi: 10.1093/nar/gkw1107. PubMed PMID: 27899635; PubMed Central PMCID: PMCPMC5210578.
41. Al-Khodor S, Price CT, Kalia A, Abu KY. Functional diversity of ankyrin repeats in microbial proteins. Trends Microbiol. 2010;18(3):132-9. doi: S0966-842X(09)00252-2 [pii];10.1016/j.tim.2009.11.004 [doi].
42. Adams MJ, Carstens EB. Ratification vote on taxonomic proposals to the International Committee on Taxonomy of Viruses (2012). Arch Virol. 2012;157(7):1411-22. doi: 10.1007/s00705-012-1299-6. PubMed PMID: 22481600.
43. Drummond AJ, Suchard MA, Xie D, Rambaut A. Bayesian phylogenetics with BEAUti and the BEAST 1.7. Mol Biol Evol. 2012;29(8):1969-73. doi: mss075 [pii];10.1093/molbev/mss075 [doi].
44. Darriba D, Taboada GL, Doallo R, Posada D. ProtTest 3: fast selection of best-fit models of protein evolution. Bioinformatics. 2011;27(8):1164-5. doi: 10.1093/bioinformatics/btr088. PubMed PMID: 21335321.
45. Paradis E, Claude J, Strimmer K. APE: Analyses of Phylogenetics and Evolution in R language. Bioinformatics. 2004;20(2):289-90. PubMed PMID: 14734327.
46. Pagel M, Meade A, Barker D. Bayesian estimation of ancestral character states on phylogenies. Syst Biol. 2004;53(5):673-84. doi: VCXNPJMTK6788EJF [pii];10.1080/10635150490522232 [doi].
47. Kass RE, Raftery AE. Bayes Factors. J Am Stat Assoc. 1995;90(430):773-95. doi: 10.1080/01621459.1995.10476572. PubMed PMID: WOS:A1995RA10400045.
48. Zuker M. Mfold web server for nucleic acid folding and hybridization prediction. Nucleic Acids Res. 2003;31(13):3406-15. PubMed PMID: 12824337; PubMed Central PMCID: PMCPMC169194.
49. Janssen S, Giegerich R. The RNA shapes studio. Bioinformatics. 2015;31(3):423-5. doi: 10.1093/bioinformatics/btu649. PubMed PMID: 25273103; PubMed Central PMCID: PMCPMC4308662.
50. Gouet P, Robert X, Courcelle E. ESPript/ENDscript: Extracting and rendering sequence and 3D information from atomic structures of proteins. Nucleic Acids Res. 2003;31(13):3320-3.
51. Risler JL, Delorme MO, Delacroix H, Henaut A. Amino acid substitutions in structurally related proteins. A pattern recognition approach. Determination of a new and efficient scoring matrix. J Mol Biol. 1988;204(4):1019-29. PubMed PMID: 3221397.
52. Heled J, Bouckaert RR. Looking for trees in the forest: summary tree from posterior samples. BMC Evol Biol. 2013;13:221. doi: 1471-2148-13-221 [pii];10.1186/1471-2148-13-221 [doi].
