# Supplementary figures and images for: A planarian nidovirus expands the limits of RNA genome size
Source: PLoS Pathog. 2018 Nov 1;14(11):e1007314. doi: 10.1371/journal.ppat.1007314 (PMC6211748; doi:10.1371/journal.ppat.1007314)

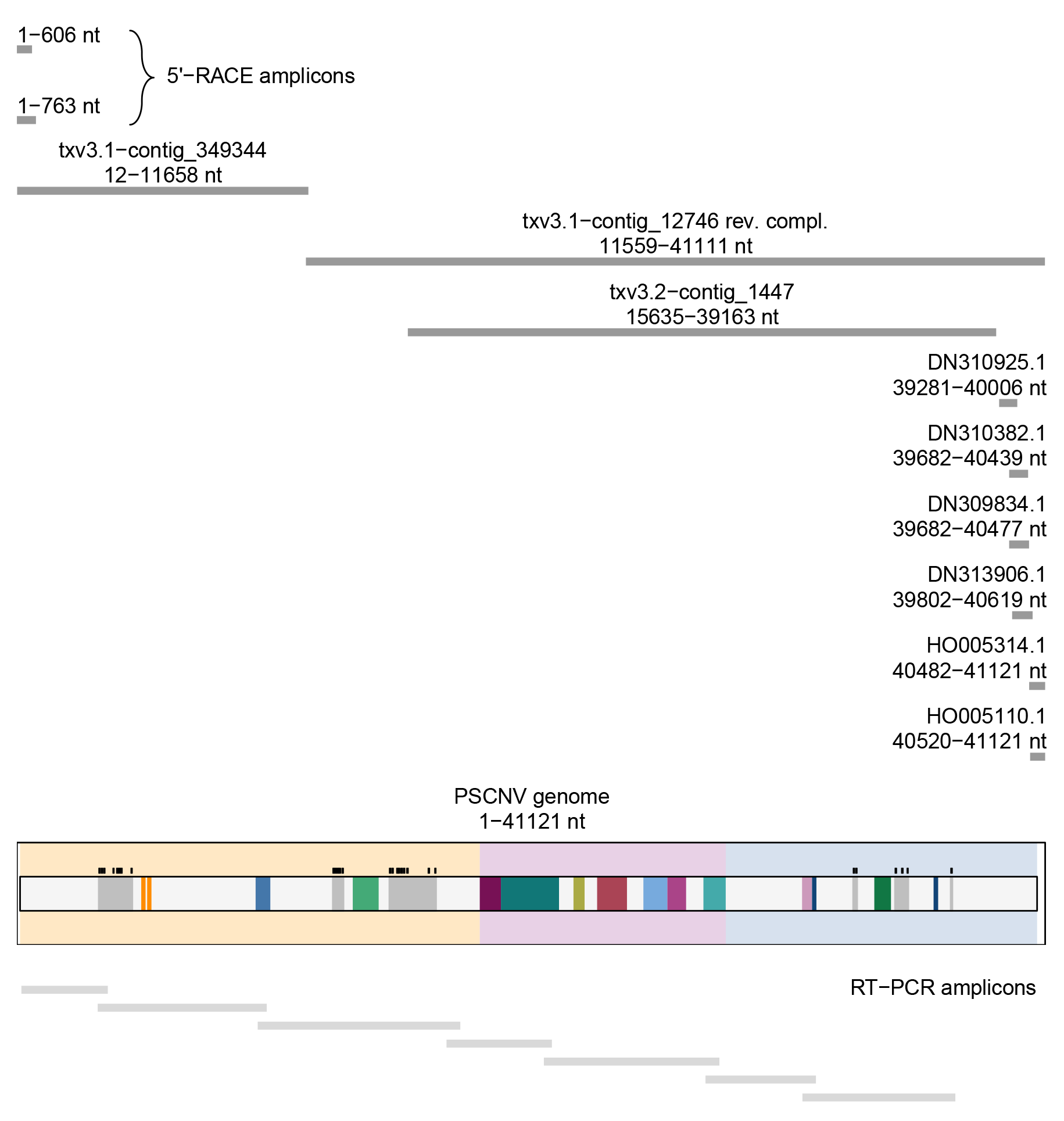

Supplement: S1 Fig — Contigs and 5’-RACE amplicons used to assemble the PSCNV genome sequence are shown above the PSCNV genome map (see Fig 2 for designations) by dark grey lines, with coordinates of the corresponding PSCNV genome regions specified on top of each line. The genome sequence was verified by obtaining products of expected sizes in seven RT-PCR reactions with pairs of primers that were designed to amplify large overlapping PSCNV genome regions (shown by light grey lines below the PSCNV genome map). (TIF) [file ppat.1007314.s002.tif]

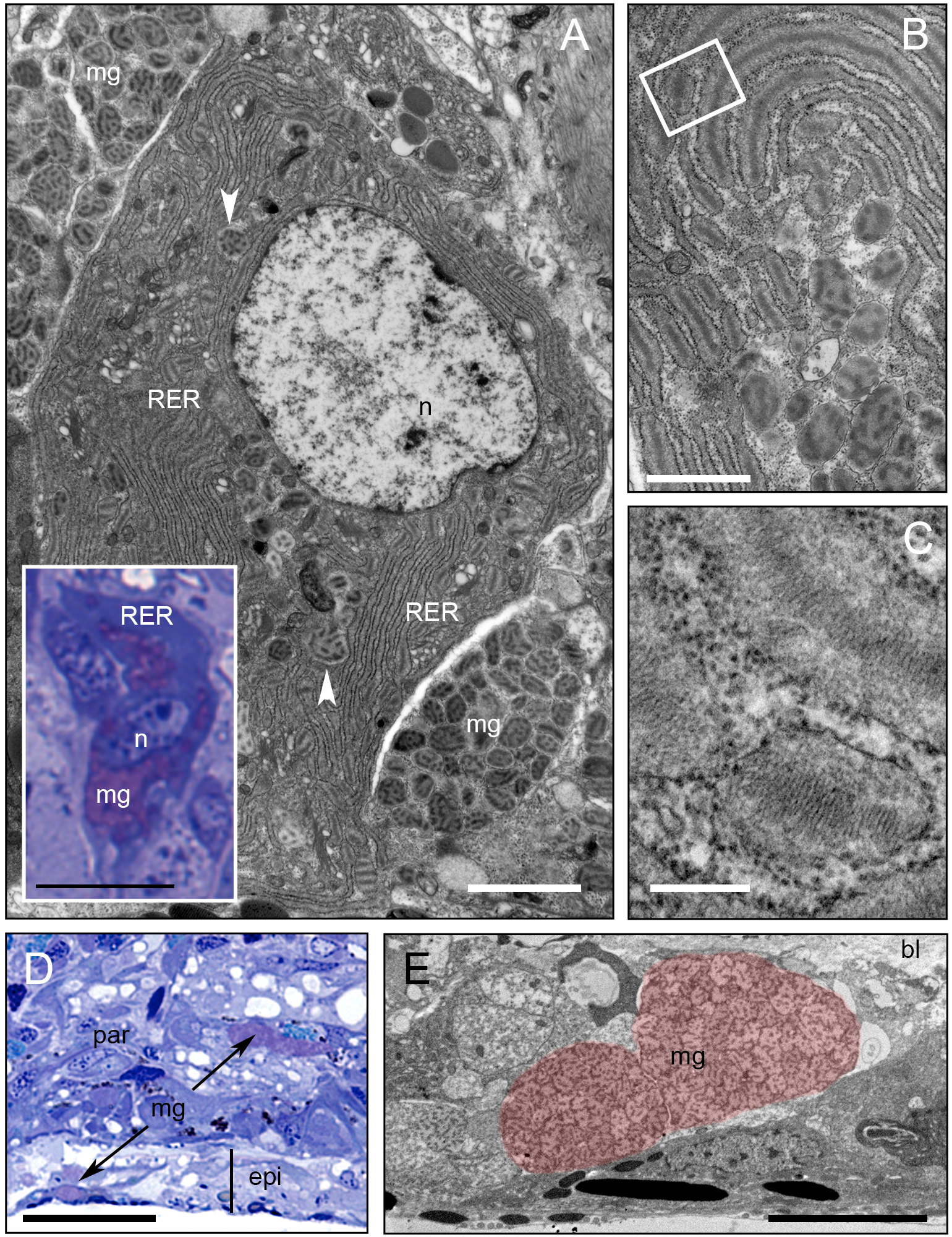

Supplement: S2 Fig — (A) Transmission electron micrograph of typical mucus cell [196]; n = nucleus. Cell bodies of such cells are filled with rough endoplasmic reticulum (RER). Distinctive mottled structures indicated by arrowheads are mucus granules. Extensions of other cells filled with these granules are also visible (mg). Inset shows a light micrograph of such a cell, stained with toluidine blue O. Mucus-rich regions of cytoplasm stain metachromatically (reddish-purple), while RER is a more-uniform blue. (B) Region of RER from mucus-cell cytoplasm (different cell from panel A) showing dilated ER lumens, and nascent mucus granules. (C) Higher magnification of RER in boxed region from panel B. (D) Light micrograph of cross section through ventral parenchyma (par) and epidermis (epi) stained with toluidine blue O. Reddish-purple patches indicated by arrows are fields of mucus granules (mg). (E) Transmission electron micrograph of ventral epithelium, showing mucus granules (mg, tinted red) just under the external surface. Scale bars: A, 2 μm (inset, 10 μm); B, 1 μm; C, 200 nm; D, 20 μm; E, 5 μm. (TIF) [file ppat.1007314.s003.tif]

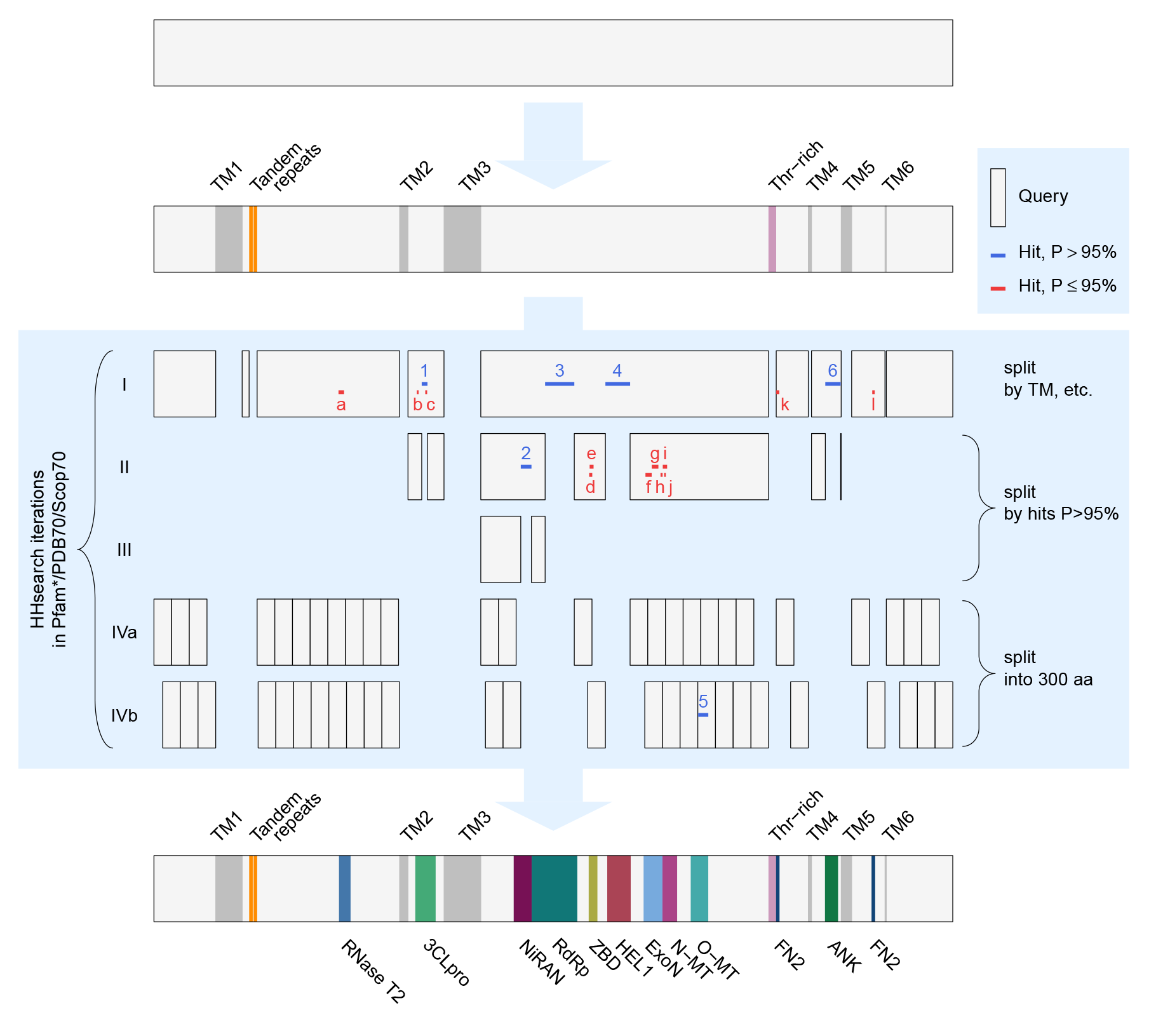

Supplement: S3 Fig — Grey bars on the top and bottom represent the PSCNV polyprotein with annotation available prior to the procedure and obtained as a result of the procedure (see S5 Table), respectively. Outline of the procedure (see S1 Materials and Methods) is presented on blue background. Iterations of the procedure are designated by Latin numbers on the left. Grey bars represent regions of PSCNV polyprotein that served as HHsearch queries to three profile databases. Basis used to split polyprotein into regions during each iteration is indicated on the right. Locations of clusters of hits with Probability (P) >95% are depicted in dark blue, with numeric indices that reflect their relative position in polyprotein, from the N- to C-terminus. Locations of accepted hits with Probability ≤95% are depicted in red, with letter indices that reflect their relative position in polyprotein, from the N- to C-terminus. (TIF) [file ppat.1007314.s004.tif]

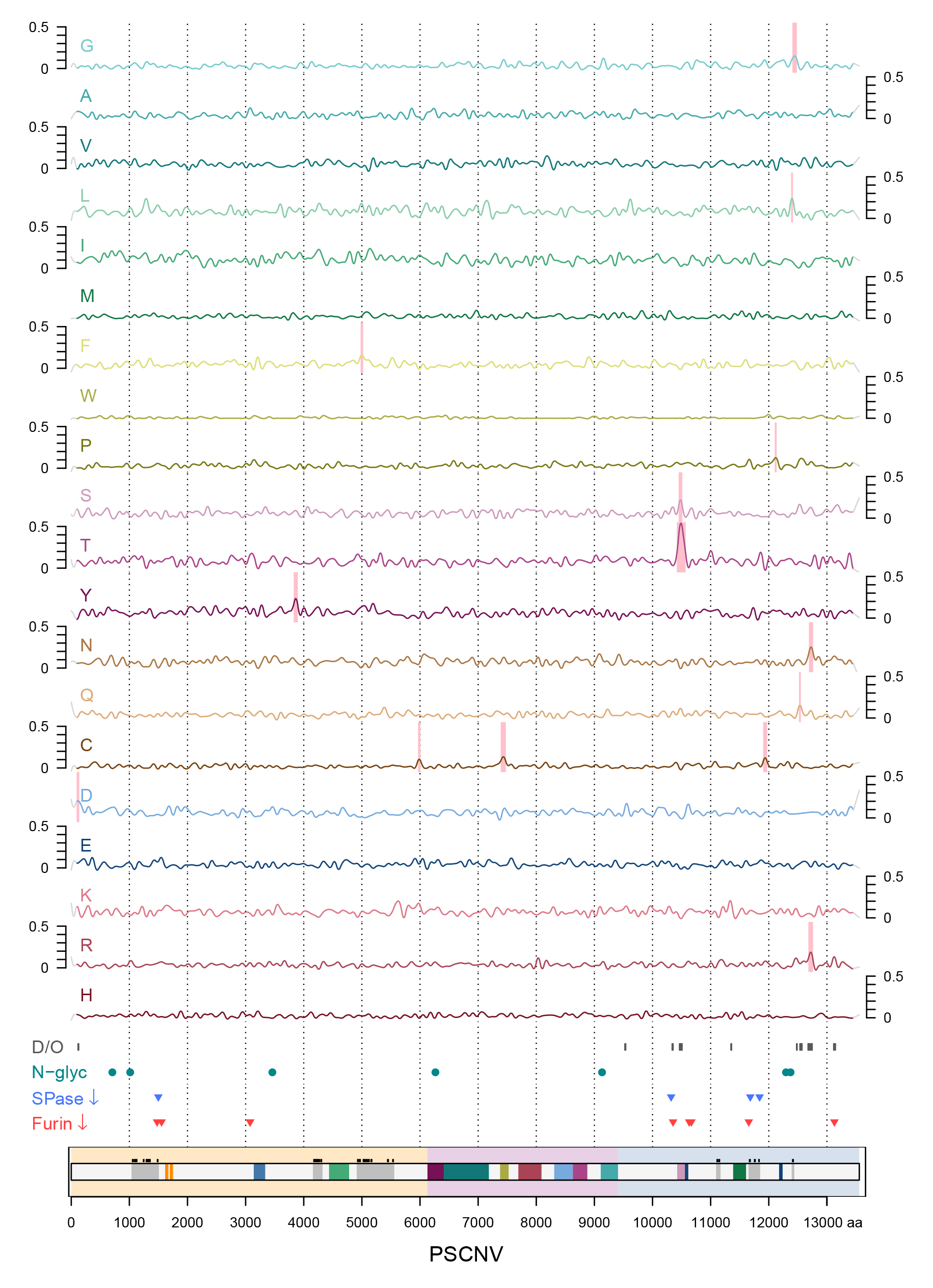

Supplement: S4 Fig — Top: first derivative of cumulative amino acid residue content is plotted for each of the 20 residues with residue-specific colors; values corresponding to the N- and C- terminal 100 residues were excluded from consideration to avoid artefacts and are shown in grey. Sites enriched with a particular residue at statistically significant level are highlighted by pink background. Bottom: polyprotein location of predicted intrinsically disordered regions (D/O), N-glycosylation sites (N-glyc), signal peptidase (SPase ↓) and furin (Furin ↓) cleavage sites are shown by grey boxes, green dots, blue and red triangles, respectively (see Fig 2 for PSCNV genome map designations). Single-letter abbreviations for the amino acid residues are as follows: G, Gly; A, Ala; V, Val; L, Leu; I, Ile; M, Met; F, Phe; W, Trp; P, Pro; S, Ser; T, Thr; Y, Tyr; N, Asn; Q, Gln; C, Cys; D, Asp; E, Glu; K, Lys; R, Arg; H, His. (TIF) [file ppat.1007314.s005.tif]

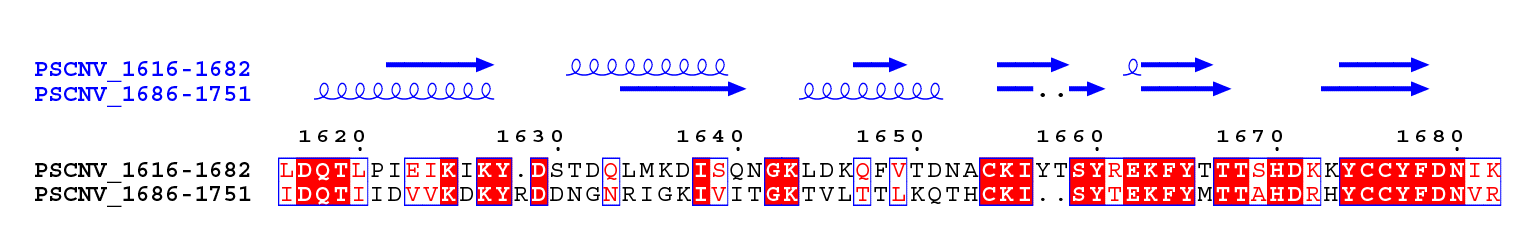

Supplement: S5 Fig — Absolutely conserved residues are shown on red background and partially conserved residues in red font. Secondary structure is shown in blue. Residue numbering on top of the alignment refers to the first repeat. (TIF) [file ppat.1007314.s006.tif]

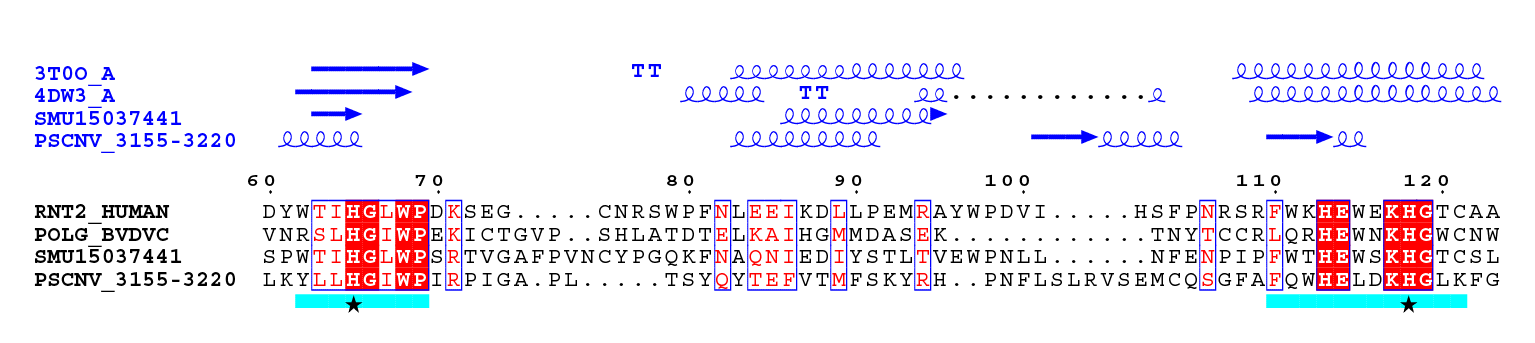

Supplement: S6 Fig — CAS I and CAS II motifs are underlined in cyan, and catalytic histidine residues are denoted with black stars. Absolutely conserved residues are shown on red background and partially conserved residues in red font. Secondary structure is shown in blue. Residue numbering above of the alignment refers to the top sequence. (TIF) [file ppat.1007314.s007.tif]

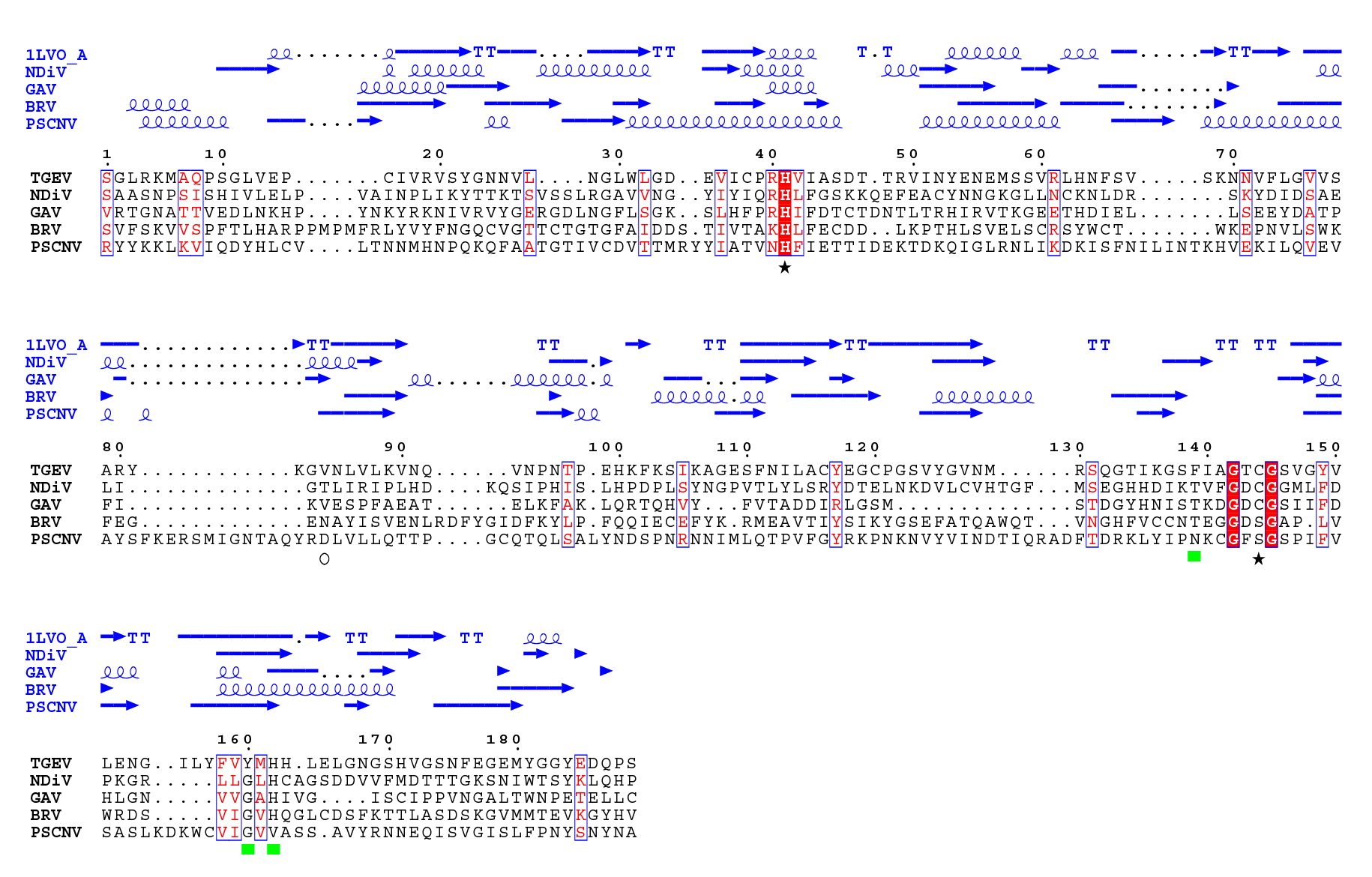

Supplement: S7 Fig — The aligned proteases employ catalytic residues that include as the principal nucleophile either a Cys residue (TGEV, NDiV, GAV) or a Ser residue (BRV, PSCNV). Columns containing TGEV 3CLpro catalytic dyad residues are marked by black stars. TGEV 3CLpro Val84 residue that is spatially equivalent to the catalytic acidic residue of serine proteases is marked with empty circle. Residues of the TGEV 3CLpro substrate-binding pocket are underlined with green bars [87]. Absolutely conserved residues are shown on red background and partially conserved residues in red font. Secondary structure is shown in blue. Residue numbering on top of the alignment refers to TGEV nsp5. (TIF) [file ppat.1007314.s008.tif]

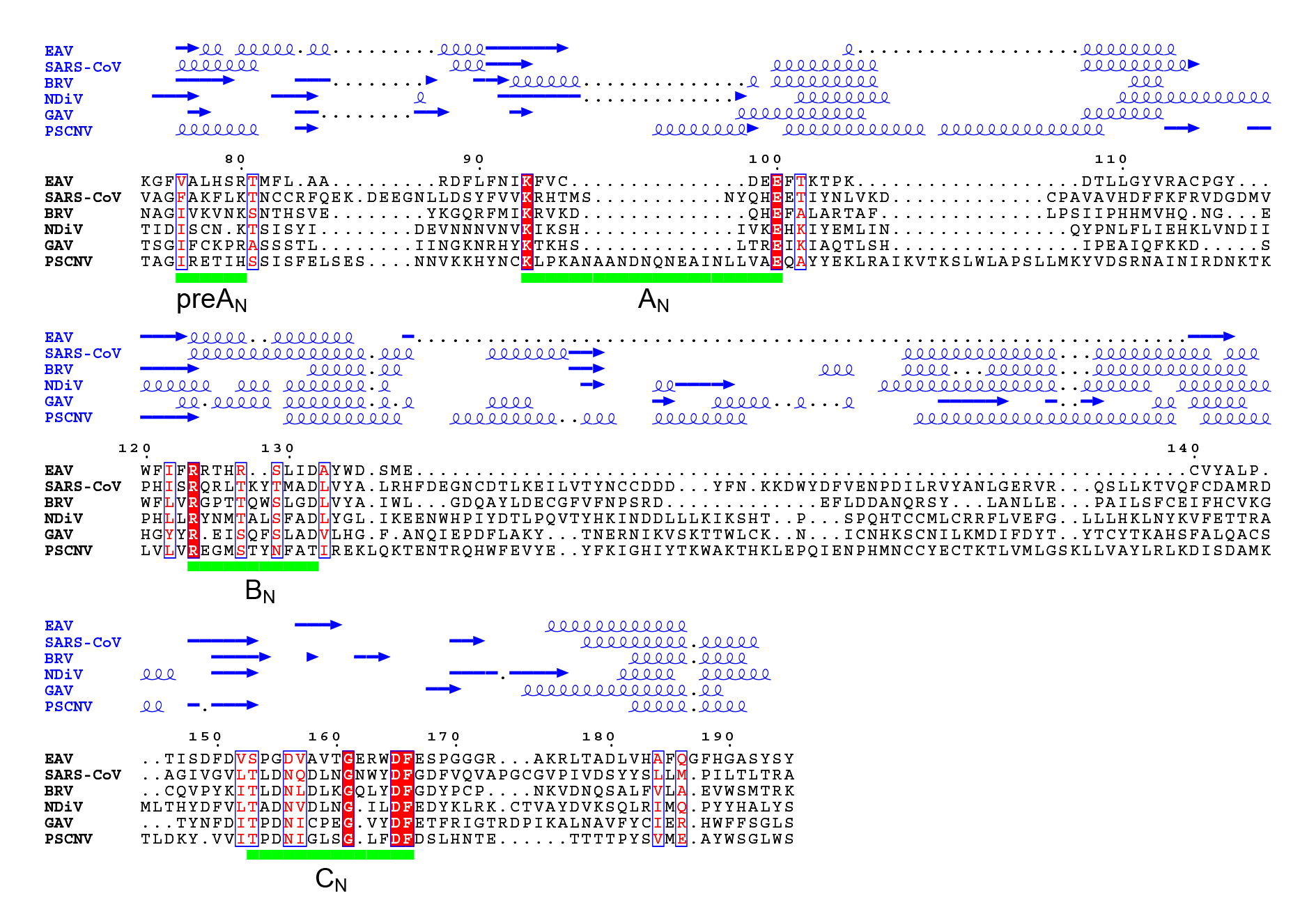

Supplement: S8 Fig — Conserved motifs are underlined in green. Absolutely conserved residues are shown on red background and partially conserved residues in red font. Secondary structure is shown in blue. Residue numbering on top of the alignment refers to EAV nsp9. (TIF) [file ppat.1007314.s009.tif]

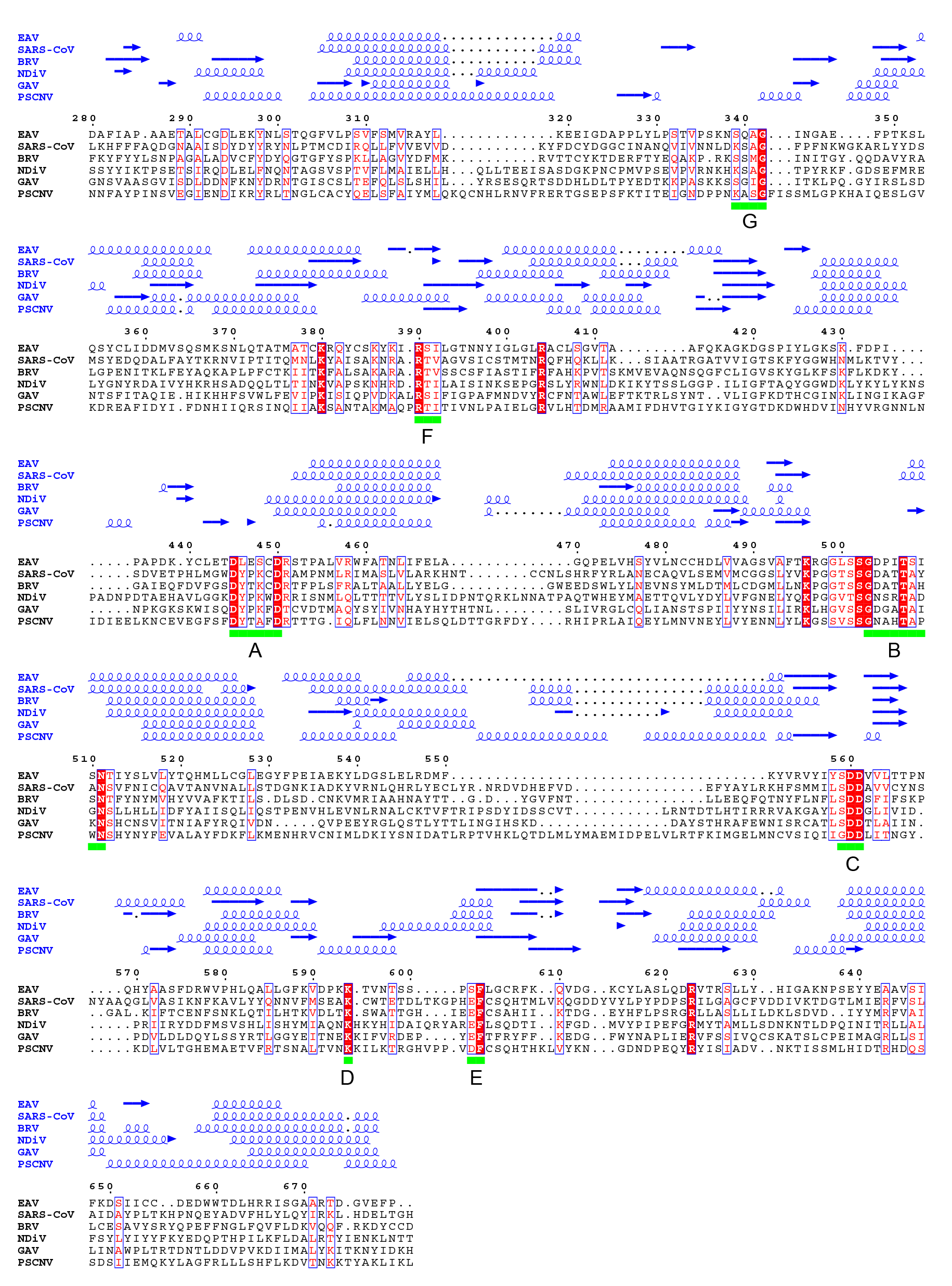

Supplement: S9 Fig — Conserved motifs are underlined in green. Absolutely conserved residues are shown on red background and partially conserved residues in red font. Secondary structure is shown in blue. Residue numbering on top of the alignment refers to EAV nsp9. (TIF) [file ppat.1007314.s010.tif]

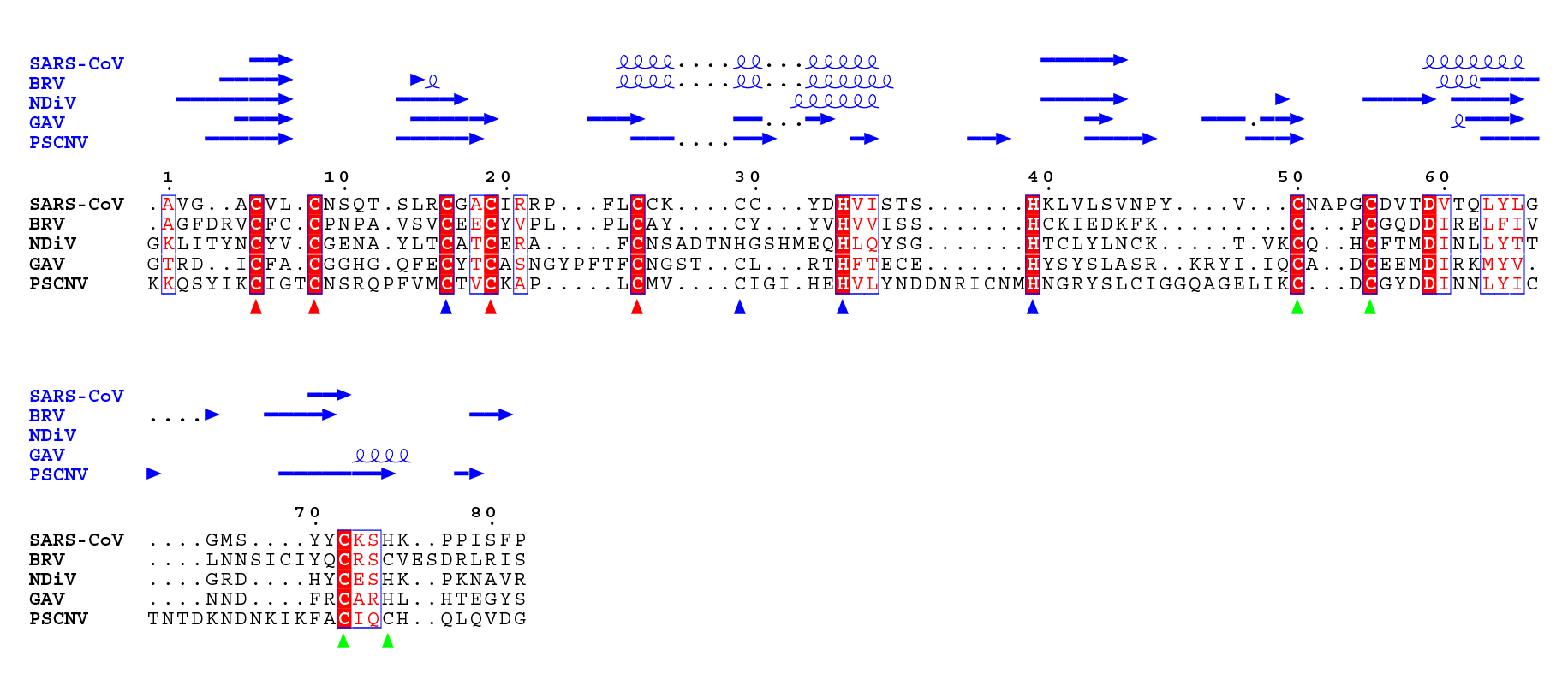

Supplement: S10 Fig — Residues of three zinc fingers coordinating zinc ions (delineated according to the solved EAV ZBD structure [48]) are marked by red, blue and green triangles, respectively. Absolutely conserved residues are shown on red background and partially conserved residues in red font. Secondary structure is shown in blue. Residue numbering on top of the alignment refers to SARS-CoV nsp13. (TIF) [file ppat.1007314.s011.tif]

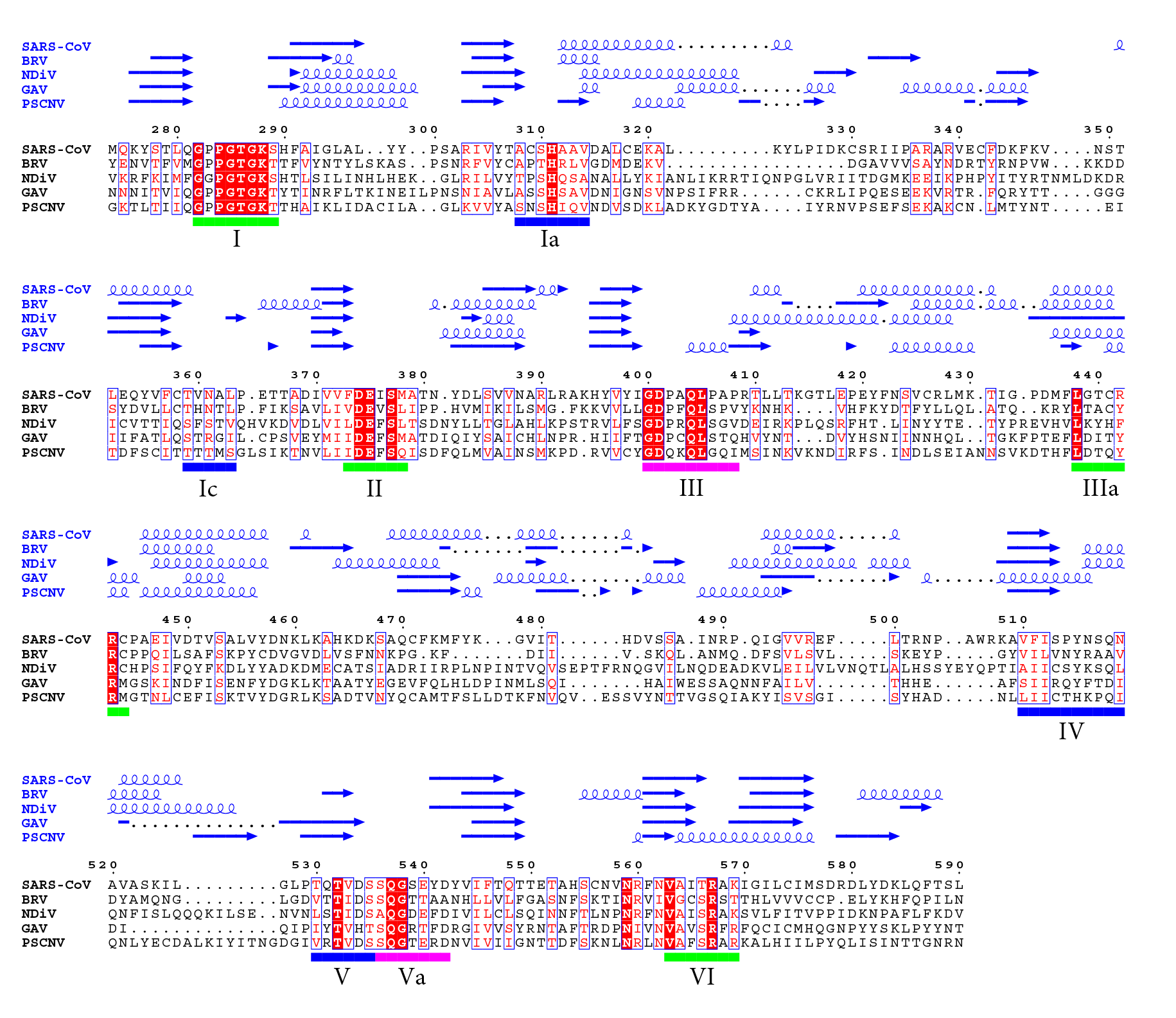

Supplement: S11 Fig — Conserved motifs are highlighted by color indicating their predominant function [47]: NTP binding and hydrolysis, green; nucleic acid binding, blue; coupling of NTP and nucleic acid binding, purple. Absolutely conserved residues are shown on red background and partially conserved residues in red font. Secondary structure is shown in blue. Residue numbering on top of the alignment refers to SARS-CoV nsp13. (TIF) [file ppat.1007314.s012.tif]

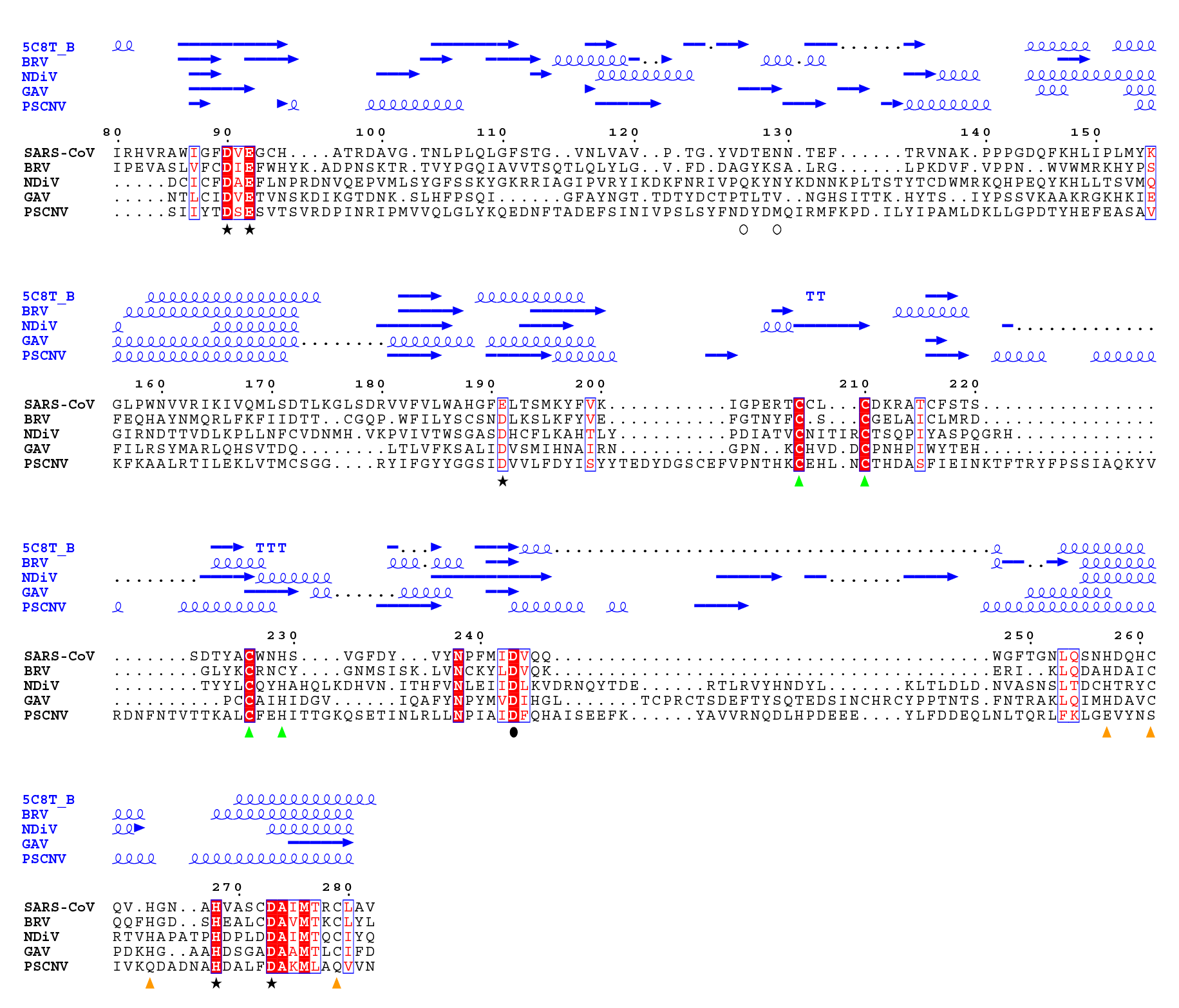

Supplement: S12 Fig — Columns containing SARS-CoV ExoN catalytic residues and Asp243 residue, essential for nuclease activity, are marked by black stars and circle, respectively. Green and orange triangles mark columns that contain residues of two SARS-CoV ExoN zinc fingers; empty circles indicate columns that contain SARS-CoV ExoN residues interacting with nsp10 (the majority of such residues are not depicted, as they belong to the N-terminal 1–76 aa region of SARS-CoV nsp14) [92]. Absolutely conserved residues are shown on red background and partially conserved residues in red font. Secondary structure is shown in blue. Residue numbering on top of the alignment refers to SARS-CoV nsp14. (TIF) [file ppat.1007314.s013.tif]

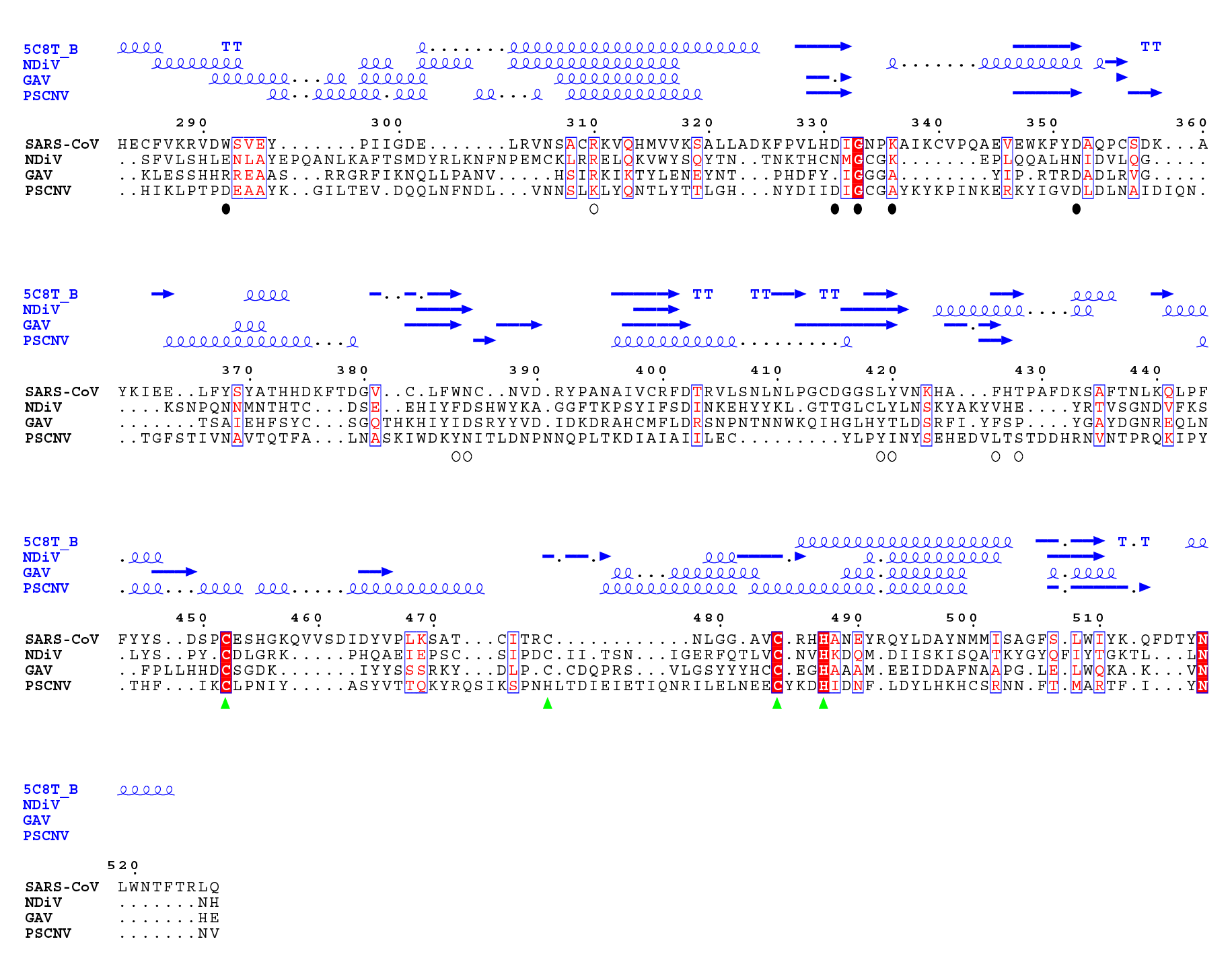

Supplement: S13 Fig — Columns containing SARS-CoV SAH- and GpppA-binding residues, such that their mutation significantly reduced N7-MTase activity, are marked by black and empty circles, respectively. Residues of SARS-CoV N-MT involved in formation of zinc-finger are marked by green triangles [92]. Absolutely conserved residues are shown on red background and partially conserved residues in red font. Secondary structure is shown in blue. Residue numbering on top of the alignment refers to SARS-CoV nsp14. (TIF) [file ppat.1007314.s014.tif]

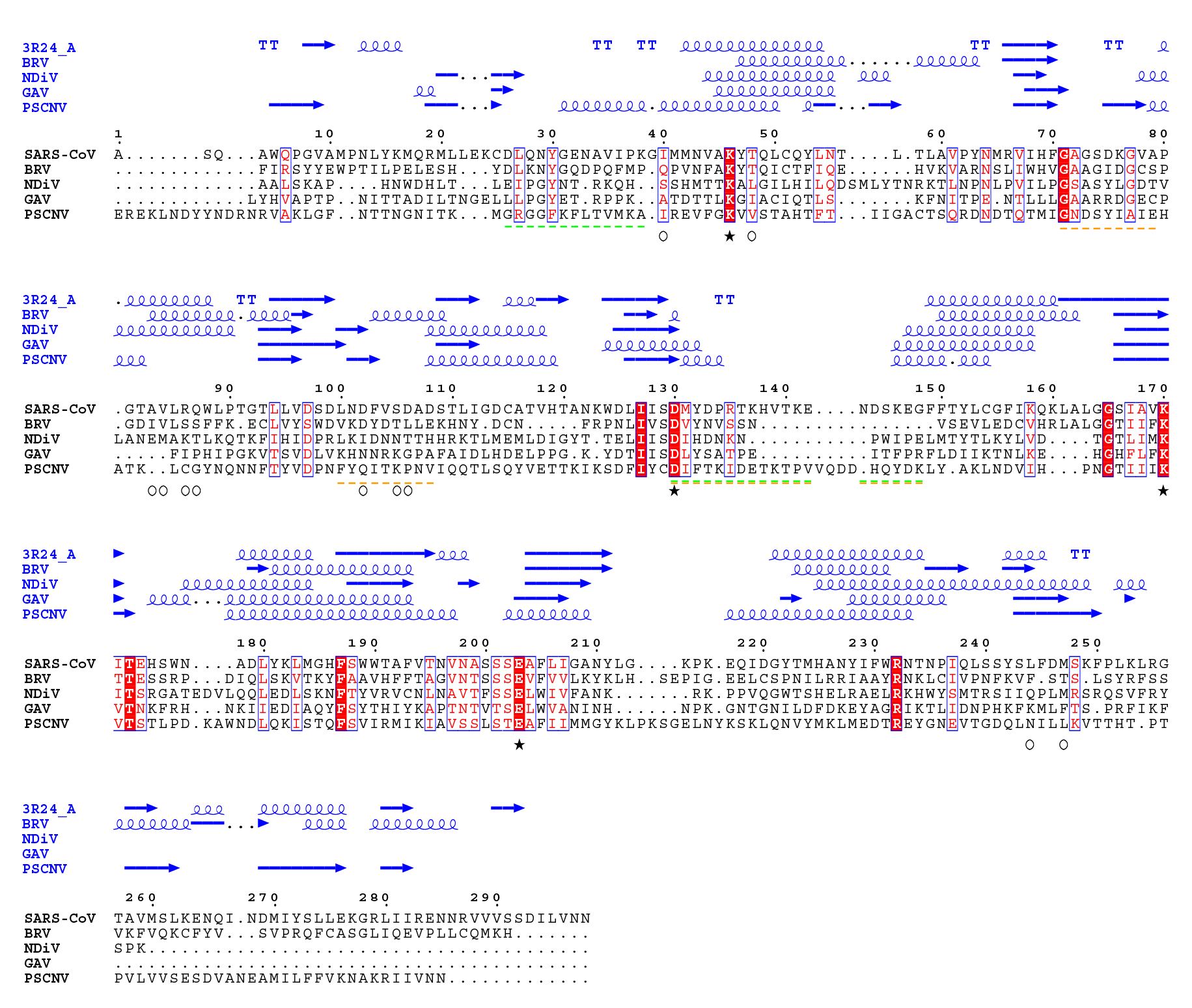

Supplement: S14 Fig — Columns containing SARS-CoV O-MT catalytic tetrad residues are marked by black stars. SARS-CoV O-MT residues involved in interaction with nsp10 are marked by empty circles. Loops constituting SAM-binding cleft and cap-binding groove of SARS-CoV O-MT are underlined in orange and green, respectively [98]. Absolutely conserved residues are shown on red background and partially conserved residues in red font. Secondary structure is shown in blue. Residue numbering on top of the alignment refers to SARS-CoV nsp16. (TIF) [file ppat.1007314.s015.tif]

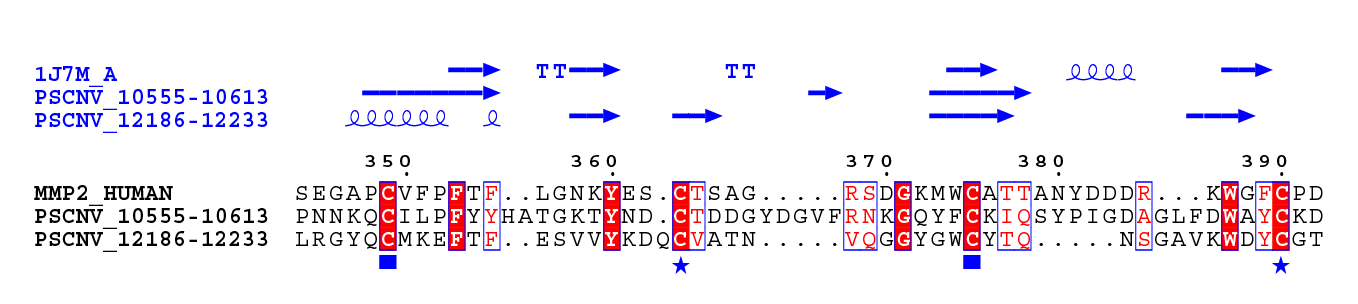

Supplement: S15 Fig — Shown is the MSA of the third FN2 domain of human matrix metalloproteinase-2 (MMP2) and FN2a (10555–10613 aa) and FN2b (12186–12233 aa) of PSCNV. Pairs of cysteine residues, predicted to form disulfide bridges, are designated by blue bars (first pair) and stars (second pair). Absolutely conserved residues are shown on red background and partially conserved residues in red font. Secondary structures, derived from MMP2 1J7M and predicted for PSCNV domains, is shown in blue. Residue numbering above the alignment refers to the top sequence. (TIF) [file ppat.1007314.s016.tif]

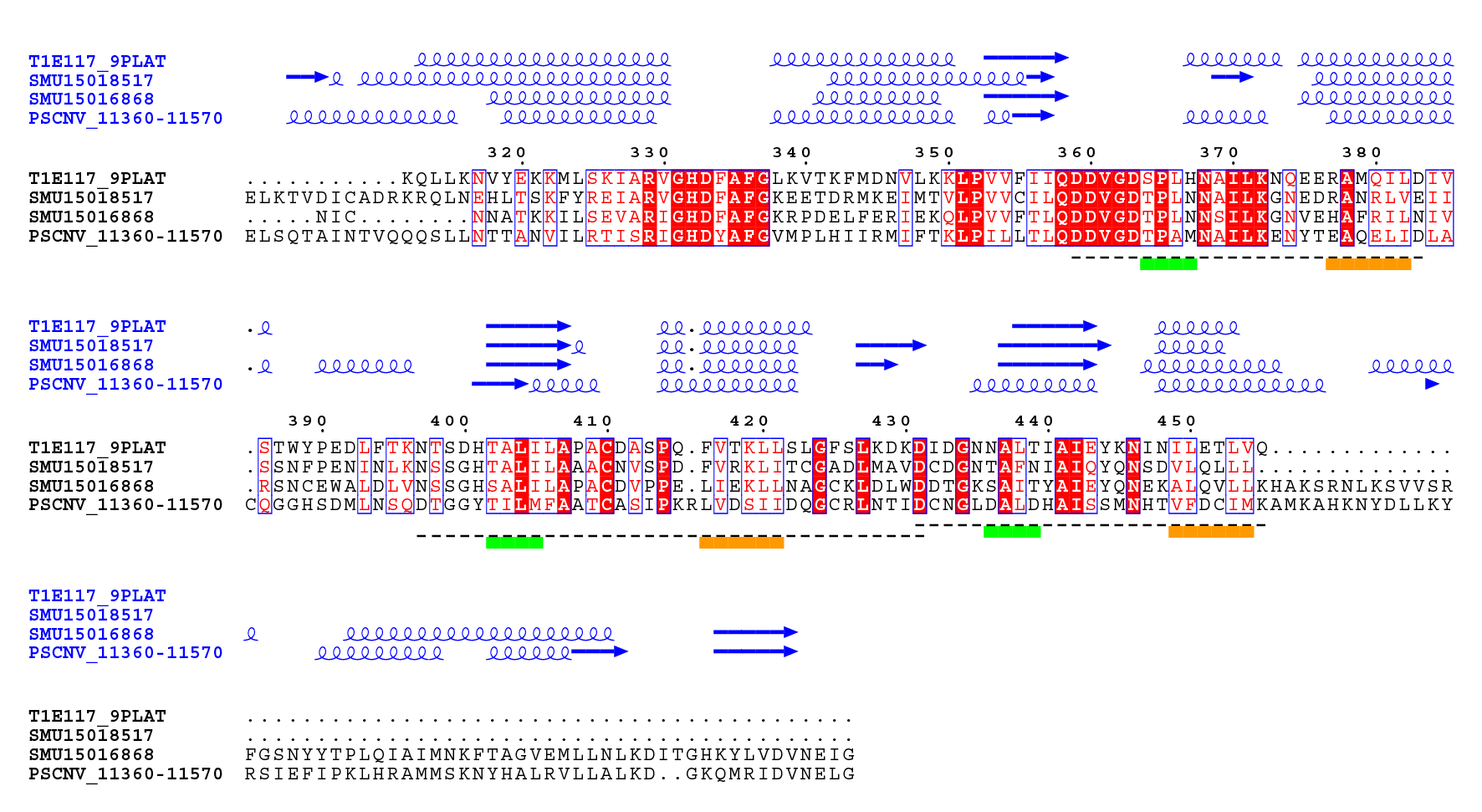

Supplement: S16 Fig — A representative set of three proteins of flatworms most closely related to the PSCNV ANK domain (Fig 6) was included in the presented MSA. Individual ankyrin repeats in PSCNV polyprotein are underlined by black dashed lines. Signature motifs of individual ankyrin repeats are highlighted in green and orange. Absolutely conserved residues are shown on red background and partially conserved residues in red font. Predicted secondary structure is shown in blue. Residue numbering above the alignment refers to the top sequence. (TIF) [file ppat.1007314.s017.tif]

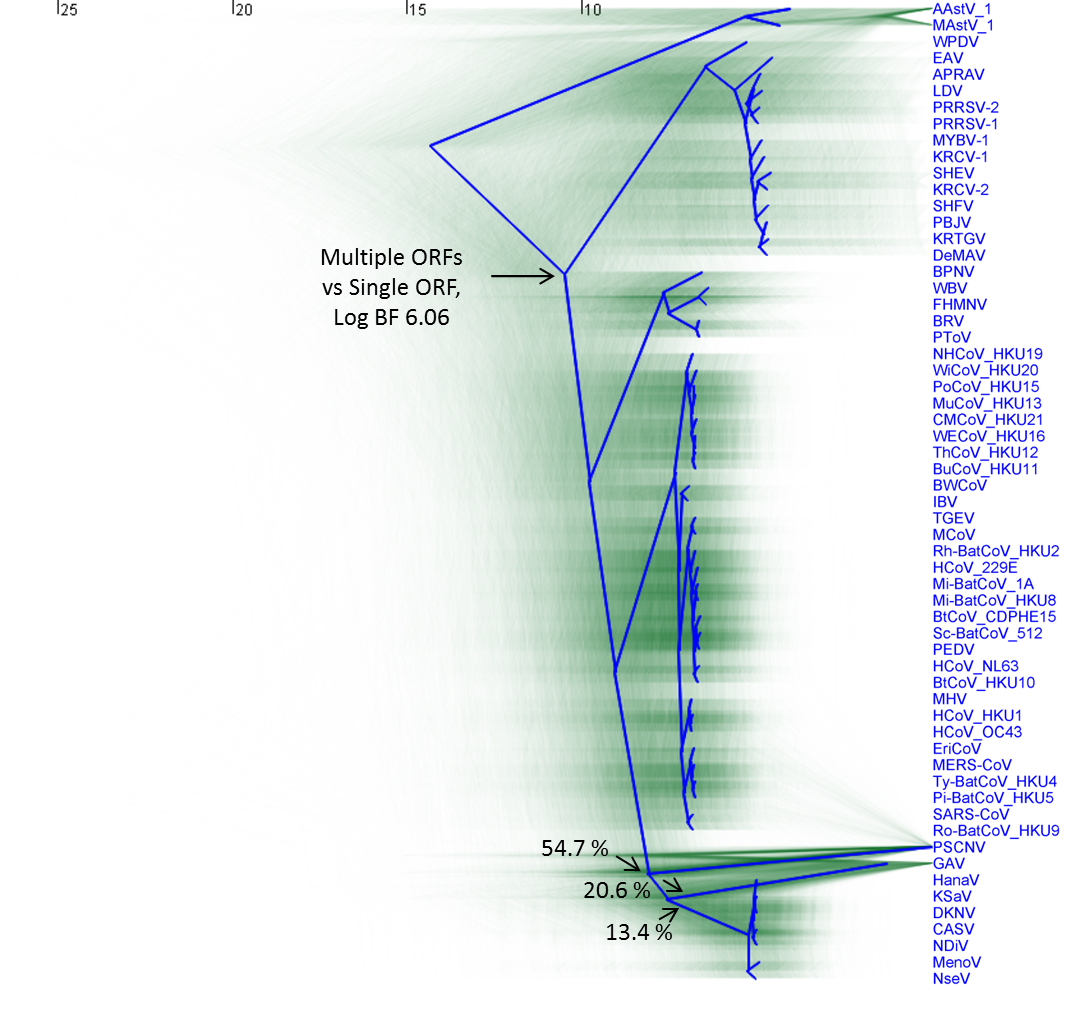

Supplement: S17 Fig — Bayesian sample of trees is shown in green, consensus tree with the highest clade support is shown in blue. Support for multiple ORFs vs single ORF in the genome of MRCA of nidoviruses as calculated using BayesTraits V2 is indicated. Short arrows show three most frequently observed (percentages of trees in the sample indicated) positions of the PSCNV branch, which collectively account for 88.7% of PSCNV topologies in the tree sample analyzed. Position of the PSCNV branch in the depicted consensus tree is the one that is most frequently observed (54.7% of trees in the sample). (TIF) [file ppat.1007314.s018.tif]

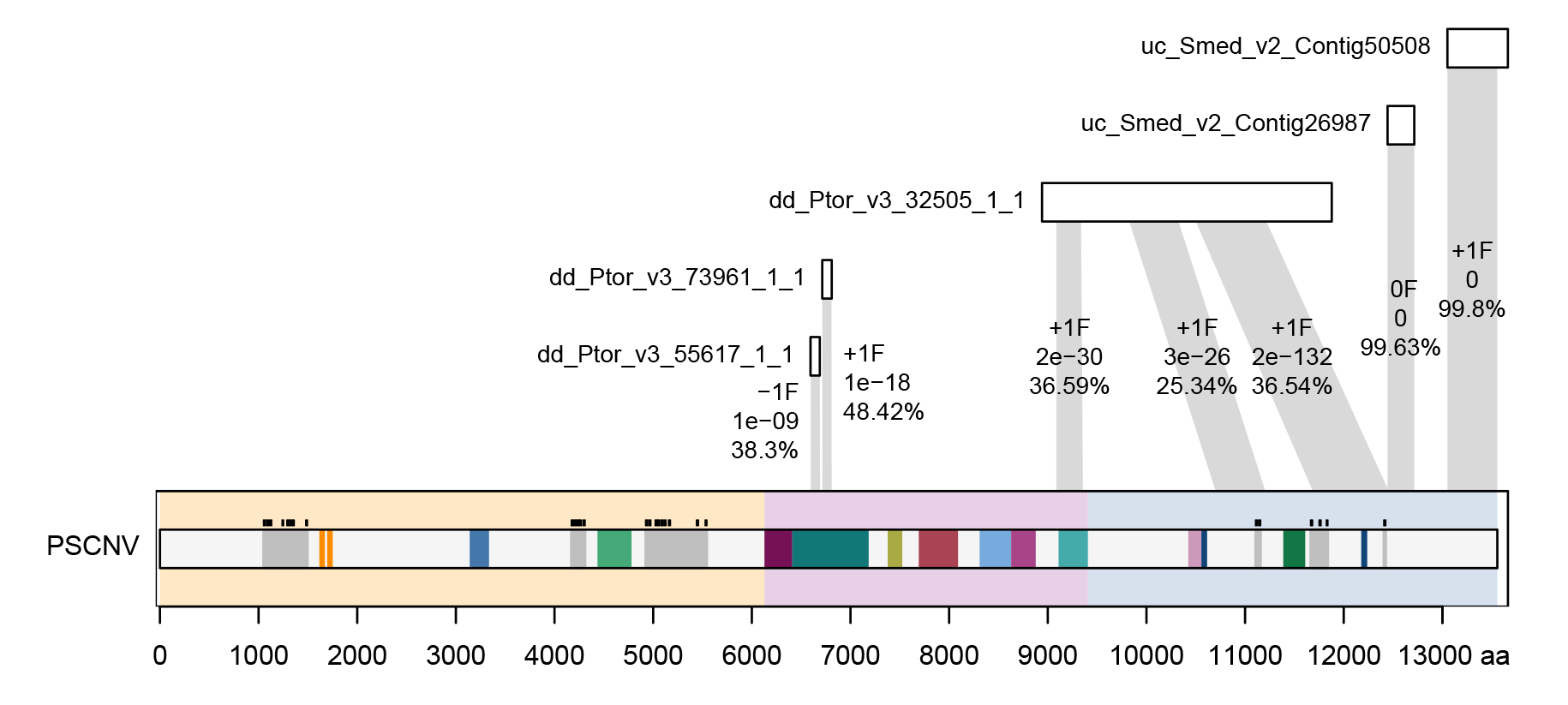

Supplement: S18 Fig — Contigs from two assemblies, dd_Ptor_v3 and uc_Smed_v2, are shown as white rectangles. For each hit, depicted as a grey band, a frame in which the contig was translated (“F” stands for forward), E-value, and percentage of amino acid identity are specified. Contig ox_Smed_v2_19364 was also identified but is not depicted due to being identical (with the exception of four 3’-terminal nt) to uc_Smed_v2_Contig50508. See Fig 2 for PSCNV genome map designations. (TIF) [file ppat.1007314.s019.tif]
