# Supplementary material for: A planarian nidovirus expands the limits of RNA genome size
Source: PLoS Pathog. 2018 Nov 1;14(11):e1007314. doi: 10.1371/journal.ppat.1007314 (PMC6211748; doi:10.1371/journal.ppat.1007314)
Supplement: S1 Table — (PDF) [file ppat.1007314.s020.pdf]

Table S1. Genome sequences and size characteristics of representatives of nidovirus species used in bioinformatics analyses.

(Sub)family	Species	Acronym	Accession number	Genome, nt	Genome region, nt		
					ORF1a	ORF1b	3'ORFs
Arteriviridae	*Equine arteritis virus*	EAV	X53459.3	12704	5181	4347	2894
Arteriviridae	*Lactate dehydrogenase-elevating virus*	LDV	U15146.1	14104	6615	4236	3018
Arteriviridae	*Porcine respiratory and reproductive syndrome virus 1*	PRRSV-1	M96262.2	15111	7185	4380	3199
Arteriviridae	*Porcine respiratory and reproductive syndrome virus 2*	PRRSV-2	U87392.3	15411	7506	4377	3189
Arteriviridae	*Simian hemorrhagic fever virus*	SHFV	AF180391.2	15717	6312	4476	4634
Arteriviridae	*Kibale red-tailed guenon virus 1*	KRTGV	JX473849.1	15264	6177	4476	4379
Arteriviridae	*Kibale red colobus virus 1*	KRCV-1	KC787630.1	15446	6141	4395	4678
Arteriviridae	*Kibale red colobus virus 2*	KRCV-2	KC787658.1	15596	6153	4458	4530
Arteriviridae	*Mikumi yellow baboon virus 1*	MYBV-1	KM110938.1	14927	6165	4461	4101
Arteriviridae	*Simian hemorrhagic encephalitis virus*	SHEV	KM677927.1	15370	6270	4401	4385
Arteriviridae	*DeBrazza's monkey arterivirus*	DeMAV	KP126831.1	15684	6249	4503	4622
Arteriviridae	*Pebjah virus*	PBJV	KR139839.1	15478	6183	4452	4615
Arteriviridae	*African pouched rat arterivirus*	APRAV	KP026921.1	14953	6717	4353	3400
Arteriviridae	*Wobbly possum disease virus*	WPDV	JN116253.3	12917	5973	4236	2351
Coronavirinae	*Alphacoronavirus 1*	TGEV	AJ271965.2	28586	12024	8031	7939
Coronavirinae	*Human coronavirus 229E*	HCoV_229E	AF304460.1	27317	12228	8049	6287
Coronavirinae	*Human coronavirus NL63*	HCoV_NL63	AY567487.1	27553	12153	8037	6791
Coronavirinae	*Miniopterus bat coronavirus 1*	Mi-BatCoV_1A	EU420138.1	28326	12777	8022	6970
Coronavirinae	*Miniopterus bat coronavirus HKU8*	Mi-BatCoV_HKU8	EU420139.1	28773	12666	8025	7575
Coronavirinae	*Porcine epidemic diarrhea virus*	PEDV	AF353511.1	28033	12324	8022	7169
Coronavirinae	*Rhinolophus bat coronavirus HKU2*	Rh-BatCoV_HKU2	EF203065.1	27164	12150	8034	6428
Coronavirinae	*Scotophilus bat coronavirus 512*	Sc-BatCoV_512	DQ648858.1	28203	12357	8025	7286
Coronavirinae	*Bat coronavirus HKU10*	BtCoV_HKU10	JQ989271.1	28489	12318	8028	7596
Coronavirinae	*Bat coronavirus CDPHE15*	BtCoV_CDPHE15	KF430219.1	28035	12453	8025	7109
Coronavirinae	*Mink coronavirus 1*	MCoV	HM245925.1	28941	12027	8022	8327
Coronavirinae	*Betacoronavirus 1*	HCoV_OC43	AY585228.1	30741	13131	8157	8929
Coronavirinae	*Human coronavirus HKU1*	HCoV_HKU1	AY597011.1	29942	13395	8154	7892
Coronavirinae	*Murine coronavirus*	MHV	AF201929.1	31276	13230	8145	9378
Coronavirinae	*Pipistrellus bat coronavirus HKU5*	Pi-BatCoV_HKU5	EF065509.1	30482	13425	8124	8353

(Sub)family	Species	Acronym	Accession number	Genome, nt	Genome region, nt		
					ORF1a	ORF1b	3’ORFs
Coronavirinae	*Rousettus bat coronavirus HKU9*	Ro-BatCoV_HKU9	EF065513.1	29114	12687	8070	7862
Coronavirinae	*Severe acute respiratory syndrome-related coronavirus*	SARS-CoV	AY274119.3[1]	29751	13134	8088	7903
Coronavirinae	*Tylonycteris bat coronavirus HKU4*	Ty-BatCoV_HKU4	EF065505.1	30286	13284	8076	8343
Coronavirinae	*Middle East respiratory syndrome-related coronavirus*	MERS-CoV	JX869059.2	30119	13155	8082	8293
Coronavirinae	*Hedgehog coronavirus 1*	EriCoV	KC545383.1	30148	13344	8109	8134
Coronavirinae	*Avian coronavirus*	IBV	M95169.1	27608	11826	8064	6685
Coronavirinae	*Beluga whale coronavirus SW1*	BWCoV	EU111742.1	31686	11865	8127	10771
Coronavirinae	*Bulbul coronavirus HKU11*	BuCoV_HKU11	FJ376619.2	26487	10746	8049	6867
Coronavirinae	*Thrush coronavirus HKU12*	ThCoV_HKU12	FJ376621.1	26396	10812	8049	6722
Coronavirinae	*Munia coronavirus HKU13*	MuCoV_HKU13	FJ376622.1	26552	10998	7926	6812
Coronavirinae	*Coronavirus HKU15*	PoCoV_HKU15	JQ065043.1	25432	10875	7929	5866
Coronavirinae	*White-eye coronavirus HKU16*	WECoV_HKU16	JQ065044.1	26041	10839	8049	6420
Coronavirinae	*Night heron coronavirus HKU19*	NHCoV_HKU19	JQ065047.1	26077	10830	8013	6553
Coronavirinae	*Wigeon coronavirus HKU20*	WiCoV_HKU20	JQ065048.1	26227	10704	7917	7114
Coronavirinae	*Common moorhen coronavirus HKU21*	CMCoV_HKU21	JQ065049.1	26223	10584	8043	6813
Torovirinae	*Bovine torovirus*	BRV	AY427798.1	28475	13332	6870	7219
Torovirinae	*Porcine torovirus*	PToV	JQ860350.1	28301	13248	6870	7199
Torovirinae	*White bream virus*	WBV	DQ898157.1	26660	13599	6969	4877
Torovirinae	*Fathead minnow nidovirus 1*	FHMNV	GU002364.2	27318	14565	6960	4813
Torovirinae	*Ball python nidovirus 1*	BPNV	KJ541759.1	33452	17394	6933	7170
Mesoniviridae	*Alphamesonivirus 1*	NDiV	DQ458789.2	20192	7491	7788	3466
Mesoniviridae	*Alphamesonivirus 2*	KSaV	KC807171.1	20795	8073	7788	3519
Mesoniviridae	*Alphamesonivirus 3*	DKNV	AB753015.2	20307	7644	7782	3493
Mesoniviridae	*Alphamesonivirus 4*	CASV	KJ125489.1	19917	7416	7782	3448
Mesoniviridae	*Alphamesonivirus 5*	HanaV	JQ957872.1	20070	7488	7776	3447
Mesoniviridae	*Mesonivirus 1*	NseV	JQ957874.1	20074	7482	7791	3488
Mesoniviridae	*Mesonivirus 2*	MenoV	JQ957873.1	19979	7404	7791	3463
Roniviridae	*Gill-associated virus*	GAV	AF227196.2	26253	12153	7869	5508

[1]To generate Fig. S12 and S13, GU553365.1 was used; to generate Fig. S14 – AY394850.2
