# Supplementary material for: A planarian nidovirus expands the limits of RNA genome size
Source: PLoS Pathog. 2018 Nov 1;14(11):e1007314. doi: 10.1371/journal.ppat.1007314 (PMC6211748; doi:10.1371/journal.ppat.1007314)
Supplement: S2 Table — (PDF) [file ppat.1007314.s021.pdf]

Table S2. Primers used for viral genome detection, 5'-RACE, and genome-wide overlapping amplification.

Primer name	Region	Sequence	Paired with	Amplicon size (bp)	Purpose
PSCNV-detect-fwd	36764..36782	AGGTGGTTATGGATGGTGT	PSCNV-detect-rev	1047	Genome detection
PSCNV-detect-rev	complement(37793..37810)	GGTGATTGATTGCGTGGT			
PSCNV-FPR-rev-606	complement(584..606)	AGACACCATCTCTTTCCATTTGT	RLM-RACE kit	606	Genomic 5'-RACE
PSCNV-FPR-rev-763	complement(744..763)	GCTATATCACCTTGGTCGCC	RLM-RACE kit	763	Genomic 5'-RACE
PSCNV-FPR-rev-28815	complement(28796..28815)	CCAAATCGGTCAAAATTCGT	RLM-RACE kit	429	Sg 5'-RACE
PSCNV-FPR-rev-29433	complement(29414..29433)	TGTCGCTTGGCATAAGTTCA	RLM-RACE kit	1047	Sg 5'-RACE
PSCNV-FPR-fwd-171	182..201	ACGAAAGGATGGCGTTCAAA	PSCNV-BlpI-rev	3456	Large amplicon 1
PSCNV-BlpI-rev	complement(3618..3637)	ACATGGGCATCTGTGAACAT			
PSCNV-BlpI-fwd	3234..3258	AGAATCCAATCATATCGACGAATTC	PSCNV-BglI-rev	6758	Large amplicon 2
PSCNV-BglI-rev	complement(9971..9991)	TCATCTGAACAACCTGTTGCT			
PSCNV-BglI-fwd	9633..9653	GGAGCACCGTTGACATCATAT	PSCNV-BstEII-rev	8101	Large amplicon 3
PSCNV-BstEII-rev	complement(17714..17733)	CGATAGCGGCAACAATCGAA			
PSCNV-BstEII-fwd	17182..17201	TAAACAGCCCACCACCAACA	PSCNV-MluI-rev	4194	Large amplicon 4
PSCNV-MluI-rev	complement(21375..21395)	AGAACTTTGGTCATGTCGTGT			
PSCNV-MluI-fwd	21076..21097	TGGGTGAGCTAATGAATTGTGT	PSCNV-AgeI-rev	7019	Large amplicon 5
PSCNV-AgeI-rev	complement(28072..28094)	AATAAAAGCCTCAGTGCTCAAAC			
PSCNV-AgeI-fwd	27539..27559	AAAGATGGGACGTGGTGGATT	PSCNV-StuI-rev	4416	Large amplicon 6
PSCNV-StuI-rev	complement(31935..31954)	GCCCAATCAAACAAGCCTGC			
PSCNV-StuI-fwd	31416..31436	CCAACAACACAACTTCGGACA	PSCNV-SacI-rev	6114	Large amplicon 7
PSCNV-SacI-rev	complement(37509..37529)	TCCACCACGGAAAAATACTCG			
