# Supplementary material for: A planarian nidovirus expands the limits of RNA genome size
Source: PLoS Pathog. 2018 Nov 1;14(11):e1007314. doi: 10.1371/journal.ppat.1007314 (PMC6211748; doi:10.1371/journal.ppat.1007314)
Supplement: S3 Table — (PDF) [file ppat.1007314.s022.pdf]

Table S3. *S. mediterranea* RNA-seq datasets screened for presence of PSCNV reads.

Laboratory	Strain	BioProject	Sequencing experiments		PSCNV reads, ppm[1]
			All	With PSCNV reads	
Aboobaker	Asexual	PRJNA79649	1[2]	0	0
Bartscherer	Asexual	PRJNA222859	8	0	0
Graveley	Asexual	PRJNA151483	3	2	10
Graveley	Sexual	PRJNA151483	6	6	69
Newmark	Asexual	PRJNA319973	15	15	19
Newmark	Sexual	PRJNA79031	4	4	1834
Pearson	Asexual	PRJNA205281	9	0	0
Pearson	Asexual	PRJNA415947	5	0	0
Rajewsky	Asexual	PRJNA79997	4	0	0
Reddien	Asexual	PRJNA320389	8	0	0
Rink	Asexual	PRJNA208294	8	0	0
Sanchez Alvarado	Asexual	PRJNA215411	1	0	0
Sanchez Alvarado	Sexual	PRJNA215411	1	0	0
Sanchez Alvarado	Sexual	PRJNA324545	40	0[3]	0
Sanchez Alvarado	Sexual	PRJNA421285	32	32	1258
Sanchez Alvarado	Sexual	PRJNA421831	15	0	0

[1]Number of reads mapped to the PSCNV reference genome sequence per million reads in the dataset.
[2]Data obtained using ABI SOLiD sequencing platform (5 runs) were not analyzed.
[3]A single read from SRR3629921 run mapped to the PSCNV genome and was considered an artefact.
