# Supplementary material for: A planarian nidovirus expands the limits of RNA genome size
Source: PLoS Pathog. 2018 Nov 1;14(11):e1007314. doi: 10.1371/journal.ppat.1007314 (PMC6211748; doi:10.1371/journal.ppat.1007314)
Supplement: S4 Table — (PDF) [file ppat.1007314.s023.pdf]

Table S4. PSCNV genome sequence variants in the 28389–41000 nt region[1].

Reference			PRJNA319973			PRJNA79031			PRJNA421285		
genome coordinate	nt	aa	p-value	nt	aa	p-value	nt	aa	p-value	nt	aa
31585	U	I	3.20E-23	C	T	3.20E-23	C	T	3.20E-23	C	T
31828	A	H		*	*		*	*	4.00E-19	G	R
35506	G	R		*	*		*	*	3.20E-23	A	K
35714	G	Q		*	*		*	*	3.20E-23	A	*
37558	G	R		*	*		*	*	0.031	A	H
37648	A	Q		*	*		*	*	3.20E-23	C	P
39112	U	I		*	*		*	*	3.20E-23	C	T
39185	U	F		*	*		*	*	3.20E-23	C	*
40748	C	Y		*	*		*	*	1.30E-20	U	*

[1]Asterisks indicate nt/aa identical to the reference.
