# Supplementary material for: A planarian nidovirus expands the limits of RNA genome size
Source: PLoS Pathog. 2018 Nov 1;14(11):e1007314. doi: 10.1371/journal.ppat.1007314 (PMC6211748; doi:10.1371/journal.ppat.1007314)
Supplement: S5 Table — (PDF) [file ppat.1007314.s024.pdf]

Table S5. Domain identification in PSCNV polyprotein through comparison with various protein databases using HHsearch (see Fig. S3 for outline).

Domain	Iteration[1]	Index[2]	Database[3]	Hit					
				Name[4]	Probability	E-value	PSCNV coo[5]	PSCNV len[6]	Template HMM[7]
RNase T2	I	a	pfam*	PF00445, Ribonuclease_T2	80	0.18	3133–3226	94	6–107 (178)
3CLpro	I	b	pdb	3k6y_A, Serine_protease	73.3	39	4462–4491	30	55–79 (237)
	I	1	scop	d2o8la1, V8 protease	95.5	0.032	4545–4641	97	90–188 (216)
	I	c	pfam*	3CLproCore_CoToMeRo	2.8	420	4605–4636	32	132–158 (187)
NiRAN	II	2	pfam*	NiRAN_CoToMeRo	95.1	0.0073	6226–6406	181	34–198 (202)
RdRp	I	3	pfam*	RdRpCore_CoToMeRo	99.1	1.00E-09	6639–7133	495	7–450 (457)
ZBD	II	d	pfam*	PF14569, Zinc-binding RING-finger	35	2.6	7387–7438	52	17–64 (77)
	II	e	pfam*	ZBD_CoToMeRo	22.7	39	7395–7460	66	13–64 (80)
HEL1	I	4	pfam*	HEL1_CoToMeRo	99.9	7.50E-28	7719–8044	326	2–307 (319)
ExoN	II	f	scop	d1w0ha, human DEDDh 3'-5'-exoribonuclease	26.2	12	8342–8446	105	7–95 (200)
	II	g	pfam*	ExoN_CoToMeRo	4.2	240	8449–8560	112	98–168 (205)
	II	h	pdb	3mxm_B, TREX1 3' Exonuclease	39.1	14	8598–8631	34	178–211 (242)
N-MT	II	i	pfam*	PF07091, Ribosomal RNA methyltransferase	80.8	0.19	8636–8708	73	46–134 (243)
	II	j	pfam*	NMT_CoMeRo	0.8	1200	8659–8686	28	24–54 (238)
O-MT	IVb	5	pfam*	OMT_CoToMeRo	96.6	0.00033	9237–9407	171	122–280 (305)
FN2a	I	k	pfam*	PF00040, Fibronectin type II domain	91.3	0.026	10561–10611	51	2–42 (42)
ANK	I	6	pdb	2rfa_A, ankyrin repeat domain of TRPV6	98.9	3.30E-08	11394–11555	162	35–218 (232)
FN2b	I	l	pfam*	PF00040, Fibronectin type II domain	78.5	0.35	12191–12231	41	1–42 (42)

[1]Iteration of HHsearch-based procedure during which hit was obtained.

[2]Index of cluster of significant hits (numeric, black font) or individual sub-significant hit (letter, grey font). For each cluster of significant hits, only the top hit is presented in the table.

[3]Databases: pfam*, pfamA_28.0 extended to include eight nidovirus domains; pdb, pdb70_06Sep14; scop, scop70_1.75.

[4]Names of nidoviral domains that were added to pfamA_28.0 have suffixes _CoToMeRo or _CoMeRo (each syllable designates a (sub)family of nidoviruses, included in the profile).

[5]Coordinates of hit in residues of PSCNV polyprotein.

[6]Length of hit in residues of PSCNV polyprotein.

[7]Coordinates of hit in match states of HMM profile from database. Number of match states in HMM profile is shown in parentheses.
