# Supplementary material for: A planarian nidovirus expands the limits of RNA genome size
Source: PLoS Pathog. 2018 Nov 1;14(11):e1007314. doi: 10.1371/journal.ppat.1007314 (PMC6211748; doi:10.1371/journal.ppat.1007314)
Supplement: S6 Table — (PDF) [file ppat.1007314.s025.pdf]

**Table S6.** Genome region size increases in PSCNV compared to ExoN-containing nidoviruses.

| Region | p, nt[1] | M, nt[2] | m, nt[3] | $D_1$, %[4] | $D_2$, %[5] | $D_3$, %[6] |
|---|---|---|---|---|---|---|
| genome | 41121 | 33452 | 27608 | 22.9 | 131.2 | 100 |
| ORF1a | 18384 | 17394 | 12153 | 5.7 | 18.9 | 14.4 |
| ORF1b | 9834 | 8157 | 8025 | 20.6 | 1270.5 | 968.1 |
| 3'ORFs | 12453 | 10771 | 6970 | 15.6 | 44.3 | 33.7 |

[1]Region size in PSCNV.
[2]Maximum region size in ExoN-containing nidoviruses.
[3]Median region size in ExoN-containing nidoviruses.
[4]$D_1$(region)=(p-M)/M*100%, PSCNV region size increase calculated accounting for the region size of ExoN-containing nidoviruses.
[5]$D_2$(region)=(p-M)/(M-m)*100%, PSCNV region size increase calculated accounting for the region size variation of ExoN-containing nidoviruses.
[6]$D_3$(region)=$D_2$(region)/$D_2$(genome)*100%, PSCNV region size increase calculated accounting for the region size variation of ExoN-containing nidoviruses and PSCNV genome size increase.
